# Quantitative detection of DNA methylation from nanopore sequencing data without raw signals

**DOI:** 10.1093/gigascience/giaf113

**Published:** 2025-10-31

**Authors:** Zhixing Feng, Chenxi Zhang, Shuo Jin, Jiale Niu, Huijuan Feng

**Affiliations:** Department of Clinical Genetics, Xinhua Hospital affiliated to Shanghai Jiao Tong University School of Medicine, Shanghai 200092, China; Department of Computational Biology, School of Life Sciences, Fudan University, Shanghai 200438, China; Department of Computational Biology, School of Life Sciences, Fudan University, Shanghai 200438, China; Department of Computational Biology, School of Life Sciences, Fudan University, Shanghai 200438, China; Department of Computational Biology, School of Life Sciences, Fudan University, Shanghai 200438, China

**Keywords:** nanopore sequencing, DNA methylation, computational methods, machine learning

## Abstract

**Background:**

Nanopore sequencing has revolutionized the field of epigenomics by enabling direct detection of DNA methylation without additional sample preprocessing such as bisulfite treatment. It is theoretically possible to reutilize any nanopore sequencing data to construct epigenomes. However, reutilizing the data in practice is challenging with existing methods because they rely on raw signals from nanopore sequencing, which are absent in more than 98% of public nanopore sequencing data. Moreover, storing raw signals for large-scale sequencing projects is impractical due to their enormous file sizes.

**Findings:**

To overcome these limitations, we propose a novel method, NanoFreeLunch, which can quantitatively detect DNA methylation without the need for raw signals by modeling base quality values and sequencing error patterns. Our results demonstrated a strong correlation between the DNA methylation levels estimated by NanoFreeLunch and those estimated by the benchmark methods, ranging from 0.87 to 0.94 for individual genomic loci and from 0.97 to 0.99 for average methylation levels of genomic regions.

**Conclusions:**

NanoFreeLunch enables detection of DNA methylation from nanopore sequencing data without raw signals. With the rapid accumulation of nanopore sequencing data, the development of NanoFreeLunch will enable the construction of epigenomes on an unprecedented scale, facilitating novel insights into the role of DNA methylation in health and disease.

## Introduction

DNA methylation plays important roles in many biological processes, such as regulating gene expression, maintaining genome stability, gene imprinting, and X chromosome inactivation [1–3]. It is also an important biomarker for diseases, including congenital disorders and cancer [4–6]. Each genomic locus can be methylated, unmethylated, or partially methylated. Although DNA methylation is a dynamic marker affected by both genetics and environment [7, 8], the methylation status of many genomic regions is precisely regulated and can cause a wide range of diseases if disturbed. For example, the 15q11-q13 region regulates the imprinting of multiple genes and is approximately 50% methylated, with only 1 of the 2 haplotypes fully methylated in healthy individuals. Full methylation of this region causes Angelman syndrome, while full unmethylation causes Prader–Willi syndrome [1]. Therefore, precise quantification of DNA methylation is critical for understanding the function of DNA methylation and determining its relationship with phenotypes and diseases.

Currently, the most widely used approaches for detecting DNA methylation quantitatively are based on next-generation sequencing (NGS) or microarrays. The sample is treated with bisulfite to mutate unmethylated cytosine to thymine while keeping 5-methylcytosine (5mC) and 5-hydroxymethylcytosine (5hmC) unchanged, and the DNA methylation level for each genomic locus can be quantified by the proportion of unmutated cytosine in NGS or the relative signal intensity in microarrays [9–11]. Although DNA methylation can be quantified with these methods, a major limitation is that they require the sample to be treated with bisulfite before sequencing or probe hybridization. Therefore, most of the existing NGS or microarray data cannot be reutilized to detect DNA methylation because many of them were designed for genotyping and not treated with bisulfite.

**Figure 1:**
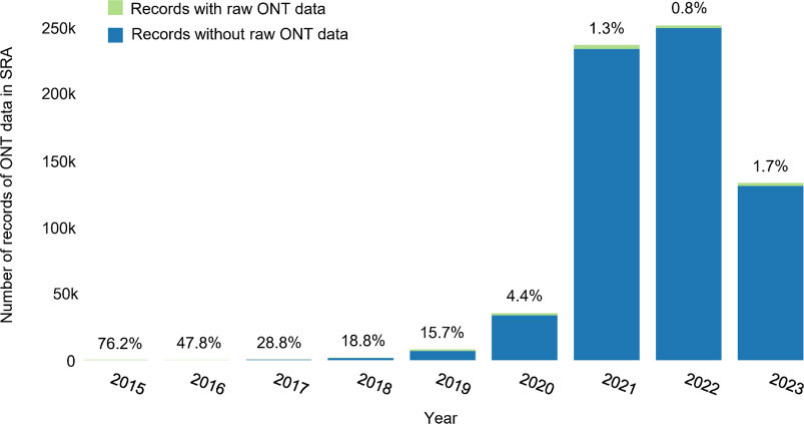
The number of records of nanopore sequencing data in the SRA database each year. The height of each bar represents the number of records. The records with or without raw signal files are represented using different colors. The percentage on the top of each bar is the ratio of records with raw signals. The bars for the years 2015 and 2016 appear barely visible due to the limited amount of released data during those periods.

Nanopore sequencing provides a revolutionary platform for generating genomes and epigenomes simultaneously since it can detect DNA methylation directly without sample preprocessing, such as bisulfite treatment or immunoprecipitation. DNA methylation is retained during the sequencing process and has an impact on the raw electrical signals detected by the sequencer [12]. A machine learning model can be built to predict DNA methylation by extracting features from the raw signals of nanopore sequencing [12]. Therefore, it is theoretically possible to reutilize any nanopore sequencing data to study DNA methylation even if the data are generated for other purposes, such as studying structural variation. However, achieving this goal is challenging in practice due to limitations in existing methods [12–19]. The existing tools, including Oxford Nanopore’s official software (e.g., Guppy and Dorado) and third-party solutions (e.g., Nanopolish, DeepMod, and DeepSignal), rely on raw signal data stored in FAST5/POD5 files for DNA methylation detection [12–19]. These raw signal files are very large, often exceeding 1 terabyte for a single ∼20× human genome dataset, making them expensive to store, difficult to transfer, and rarely shared in public repositories. For example, in the Sequence Read Archive (SRA) database [20], there are 742,566 records of Oxford Nanopore genome sequencing (ONT) data as the study was conducted, but only 1.5% of the data include raw signal files, and the percentage has decreased over the years (Fig. 1). This makes it impossible to harness large-scale DNA methylation information from most datasets with the existing methods. As large-scale nanopore sequencing data accumulate rapidly [21], it is also unsustainable to store all the raw signal files of nanopore sequencing because the size of 20,000 20× human genome datasets is approximately 20 petabytes, which is almost the total size of the SRA database [22]. More than 4,000 nanopore sequencing datasets of human genomes have been published in the past 3 years [21, 23], and the accumulation of such data has accelerated remarkably in recent years (Fig. 1). Therefore, alternative approaches for detecting DNA methylation without reliance on raw signal files are urgently needed.

To address the challenge of reutilizing nanopore sequencing data to construct epigenomes, we introduce NanoFreeLunch, a computational framework for quantitatively detecting DNA methylation from basecalled FASTQ files via a novel approach to model base quality value (QV) and sequencing error patterns. NanoFreeLunch has undergone extensive testing on 3 independent datasets of 16 nanopore sequencing experiments. The DNA methylation levels predicted by NanoFreeLunch are highly consistent with those predicted by benchmarking methods, including raw signal–based algorithms and conventional bisulfite sequencing. The correlation ranges from 0.87 to 0.94 for the DNA methylation level of each CpG site and from 0.97 to 0.99 for the average methylation level of the CpG islands. The results of NanoFreeLunch are also consistent with established epigenetic knowledge. The partial methylation of imprinting control regions (ICRs), hypomethylation of regions with H3k4me3 histone modification, and hypermethylation of regions with H3k9me3 histone modification can be reliably detected by NanoFreeLunch. As nanopore sequencing data accumulate rapidly, NanoFreeLunch represents a powerful tool enabling the construction of epigenomes on an unprecedented scale by reutilizing the existing data and establishing the relationships among DNA methylation, genotypes, and phenotypes.

## Results

### Detecting DNA methylation by modeling sequencing error patterns and base quality values

**Figure 2:**
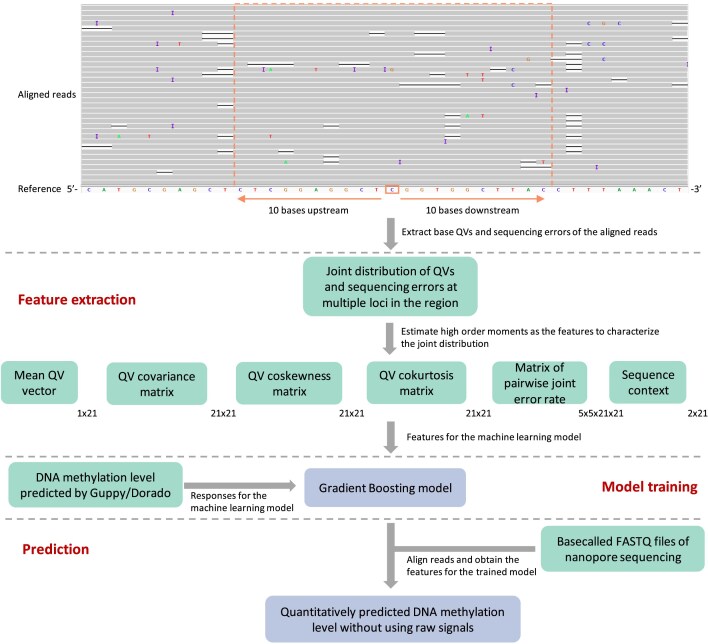
The workflow of NanoFreeLunch. There are 3 major components in NanoFreeLunch. The first component, feature extraction, constructs the features from aligned reads of potentially methylated loci. The second component, model training, utilizes the extracted features and DNA methylation levels predicted by Guppy or Dorado to train a boosting model. The third component, prediction, leverages the trained model to predict DNA methylation levels from the features extracted from the aligned reads. The aligned reads are displayed in an IGV snapshot. IGV stands for the Integrative Genomics Viewer [24].

NanoFreeLunch leverages the impact of DNA methylation on base QVs and error patterns to quantitatively detect DNA methylation. As we have previously reported, DNA methylation has an impact on the error patterns of nanopore sequencing data [23]. For each genomic locus of interest, we obtained the aligned reads covering the region from 10 bases upstream to 10 bases downstream and used the joint probability distribution of basecalling QVs and pairwise joint sequencing error rates in these 21 loci as features to predict the DNA methylation level (proportion of the methylated bases at the locus). Because it is difficult to use joint probability directly as the input of a machine learning model, we characterize the distribution by combining high-order moments (Fig. 2): (i) first-order moment, the mean QV of each locus; (ii) second-order moment, the covariance of QVs; (iii) third-order moment, the coskewness of QVs; and (iv) fourth-order moment, the cokurtosis of QVs. Since sequence context affects the QV and error rate, the sequences of the 21 loci are also included in the feature list. The details of feature extraction are described in the Methods section.

In this study, we used the DNA methylation level at each CpG locus predicted by Guppy 6.3.8 or Dorado 0.5.3 as the “known” methylation level and the features obtained from basecalling QVs and sequencing errors to train a gradient boosting regression model [25] (Fig. 2). The details of model training are described in the Methods section. Guppy [26] and Dorado [27] are toolsets provided by Oxford Nanopore for basecalling and base modification calling. They can be used as the benchmark since the predicted locus-level DNA methylation is highly consistent with that obtained by bisulfite sequencing (the Pearson correlation coefficient [PCC] is approximately 0.95 [28, 29]). By comparing hypermethylated and hypomethylated loci predicted by Guppy in the training data, all the features exhibit differences between them (Supplementary Fig. S1). By evaluating the accuracy of NanoFreeLunch with different features on human pangenome data, the results show that while each feature has some ability to predict DNA methylation levels, combining all the features yields the most accurate model (Supplementary Fig. S2).

### Comparing NanoFreeLunch with benchmark methods

In this study, we adopt 2 independent benchmark methods. The first is bisulfite sequencing, which is a traditional NGS-based method for detecting DNA methylation. The second one is Guppy/Dorado, the raw signal–based method provided by Oxford Nanopore, to detect base modifications from raw signals of nanopore sequencing data. NanoFreeLunch was evaluated on 3 independent datasets. The first dataset was obtained from the ONT open dataset released by Oxford Nanopore [29], which sequences the GM24385 (HG002) cell line by MinION with the R9.4.1 flowcell. The second dataset included 9 samples from the Human Pangenome Project (HPGP) [30] sequenced by PromethION with the R9.4.1 flowcell (a part of sample HG01109 was used as the training data and excluded from evaluation) [31]. The third dataset is the Ashkenazim trio (HG002, HG003, and HG004), sequenced by PromethION with the R10.4.1 flowcell. Each sample was sequenced at 4-kHz and 5-kHz sampling rates. The data are downloaded from [32] and [33].

**Figure 3:**
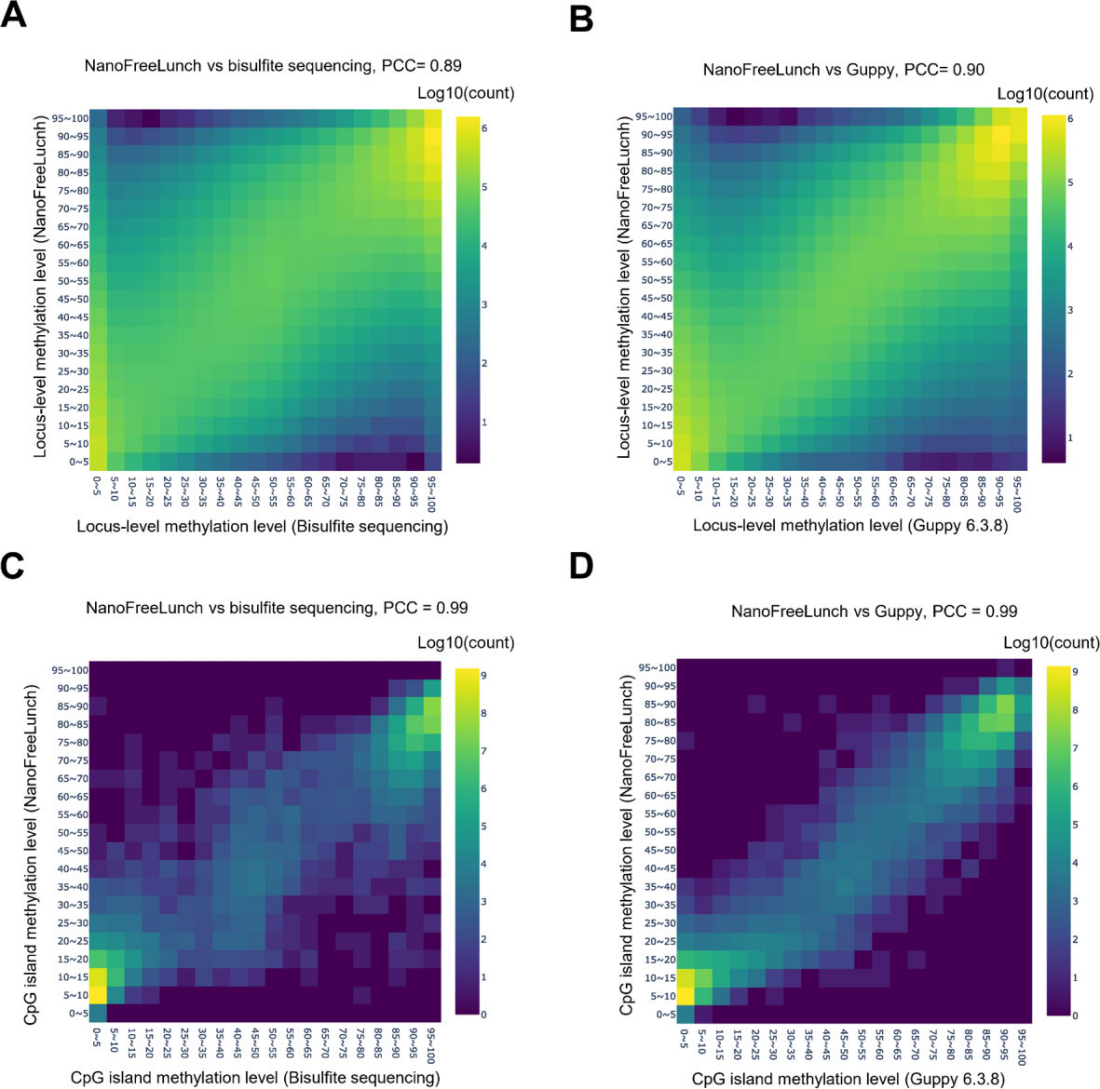
Performance evaluation of NanoFreeLunch. (A, B) Comparing the locus-level methylation levels estimated by NanoFreeLunch with bisulfite sequencing and Guppy 6.3.8 in the HG002 dataset. (C, D) Comparing the average methylation level of CpG islands estimated by NanoFreeLunch with bisulfite sequencing and Guppy 6.3.8 in the HG002 dataset. The predicted DNA methylation levels were segmented into 20 bins of equal size ranging from 0% to 100%. The color of each bin represents the base-10 logarithm transformation of the number of loci or regions within the bin. PCC denotes the Pearson correlation coefficient.

In the first dataset, we used Guppy 6.3.8 for basecalling from the raw electrical signals of the HG002 R9.4.1 data and used NanoFreeLunch to estimate the DNA methylation level of each CpG site from the basecalled data. The bisulfite-based and raw signal–based DNA methylation levels were downloaded from [29]. The results show that the PCCs between NanoFreeLunch and these benchmark methods are 0.89 and 0.90, respectively (Fig. 3A and Fig. 3B). In the second dataset, the basecalling results of multiple versions of Guppy (versions 2.3.5, 4.2.2, and 6.3.8) are used as the input of NanoFreeLunch because these major versions implement different basecalling algorithms. Raw signal–based DNA methylation calling was performed with Guppy 6.3.8 (Methods). The PCC between NanoFreeLunch and Guppy ranged from 0.87 to 0.94 (Supplementary Fig. S3, Supplementary Fig. S4, and Supplementary Fig. S5). We used the same basecaller version for the training and testing data. In the third dataset, we used Dorado 0.5.3 to convert the raw signals to basecalled DNA sequences and NanoFreeLunch to estimate the DNA methylation level of each CpG site. The raw signal–based DNA methylation levels were obtained using the modification calling mode of Dorado 0.5.3. The PCC between NanoFreeLunch and Dorado ranges from 0.89 to 0.93 (Supplementary Fig. S6).

Cytosine methylation is spatially correlated [33], and the overall methylation state of genomic regions is commonly used in studying the association between DNA methylation and diseases or phenotypes [34]. Therefore, we also evaluated the performance of NanoFreeLunch in estimating the methylation level of genomic regions. In this study, we used CpG islands for the evaluation. By averaging the methylation levels of the loci in each CpG island, the PCC between the regional average methylation levels predicted by NanoFreeLunch and bisulfite sequencing was 0.99 (Fig. 3C). By comparing NanoFreeLunch with Guppy/Dorado, the PCC ranged from 0.97 to 0.99 (Fig. 3D, Supplementary Fig. S7, Supplementary Fig. S8, Supplementary Fig. S9, and Supplementary Fig. S10). These results demonstrate that NanoFreeLunch can restore DNA methylation accurately without using raw signals.

### The impact of flowcell type and basecaller version on the accuracy of NanoFreeLunch

Flowcell type and basecaller version have an impact on the results of NanoFreeLunch since they might produce different errors and QV patterns. We compared the accuracy of NanoFreeLunch for different flowcell types (R9.4.1 and R10.4.1) and basecaller versions (Guppy 2.3.5, 4.2.2, and 6.3.8 for R9.4.1 and Dorado 0.5.3 for R10.4.1). The results show that the differences in accuracy are limited. The maximum accuracy difference is 0.04, according to a comparison of the Guppy 4.2.2 and Guppy 6.3.8 data (Supplementary Fig. S11). Therefore, despite the significant differences in the sequencing error rate, flowcell type and basecaller version have limited impacts on the accuracy of NanoFreeLunch.

### Estimating the DNA methylation level of imprinting control regions with NanoFreeLunch

**Figure 4:**
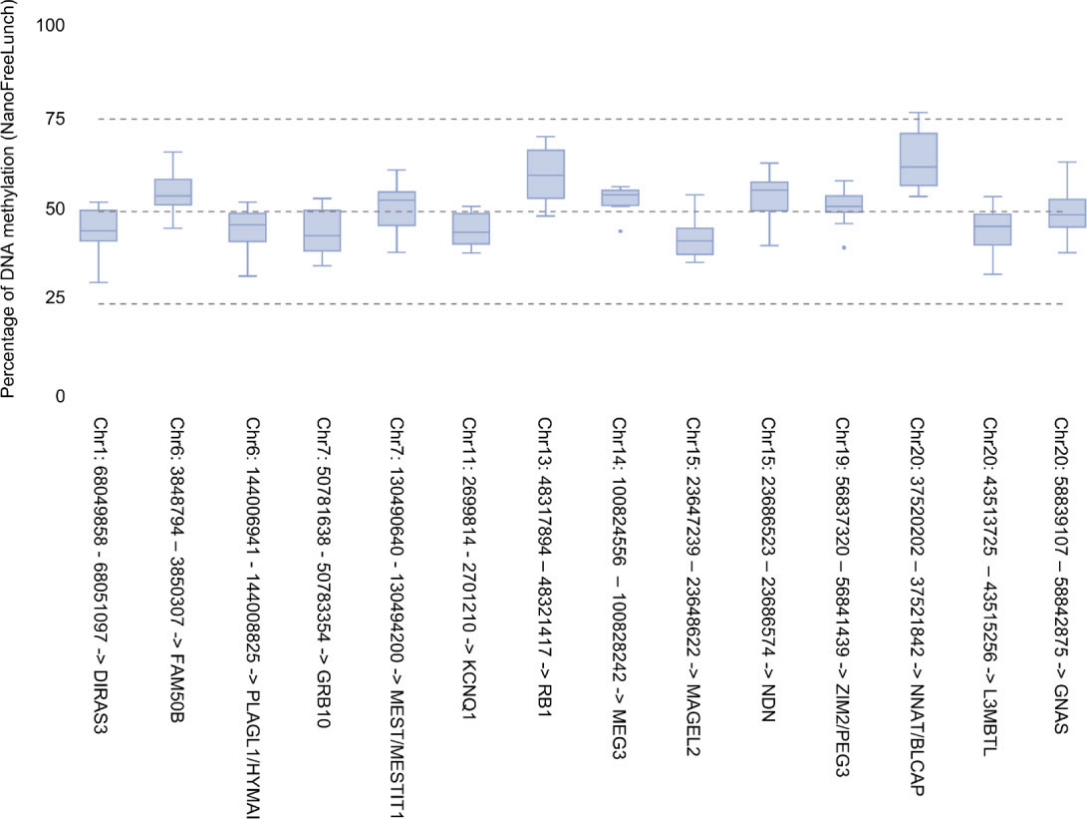
The average DNA methylation level estimated by NanoFreeLunch in ICRs. Each boxplot illustrates the distribution of the average DNA methylation levels of ICRs predicted by NanoFreeLunch using human pangenome data. The line in each box represents the median. The lower and upper bounds of the box correspond to the first (Q1) and third (Q3) quartiles, respectively. The lower fence is determined as the last sample point below 1.5 times the interquartile range (IQR), calculated as Q3 minus Q1. Similarly, the upper fence is identified as the last sample point above 1.5 times the IQR. On the x-axis, the region on the left of “→” is the genomic region of the ICR on GRCh38, and the gene symbol on the right is the putative gene associated with the ICR. The ICRs and their associated genes are obtained from previous publications [35, 36]. The basecalling results of Guppy 6.3.8 are used as the input of NanoFreeLunch.

A key feature of NanoFreeLunch is the quantitative detection of DNA methylation, which means that it can report the percentage of methylated bases for each genomic locus without using raw signals. Partially methylated regions such as ICRs that regulate gene imprinting play a critical role in human development and diseases [1]. To evaluate the performance of NanoFreeLunch in detecting partially methylated genomic regions, we used 14 ICRs with hypermethylated DNA from only 1 of the parents confirmed by multiple previous studies [35, 36] and calculated the average NanoFreeLunch-predicted methylation level in these regions using samples from the HGPG dataset (R9.4.1 flowcell and basecalling with Guppy) and Ashkenazim trio dataset (R10.4.1 flowcell and basecalling with Dorado). Utilizing basecalling outcomes from either Guppy 6.3.8 or Guppy 2.3.5 as input, all 14 ICRs exhibited a median methylation level within the range of 0.25 to 0.75, which encapsulates the middle 50% of the [0,1] range (Fig. 4 and Supplementary Fig. S12). Likewise, employing basecalling outcomes from Guppy 4.2.2 or Dorado 0.5.3 yields comparable results, with 13 of the 14 ICRs displaying a median methylation level within the 0.25 to 0.75 range (Supplementary Fig. S13 and Supplementary Fig. S14). When examining the trimmed means of predicted ICR methylation levels, NanoFreeLunch prediction with basecalling using Guppy 2.3.5, Guppy 4.2.2, Guppy 6.3.8, and Dorado 0.5.3 revealed values of 0.54, 0.59, 0.50, and 0.57, respectively. Correspondingly, the associated trimmed standard variances are 0.09, 0.08, 0.06, and 0.13 (Methods). As a benchmark, when considering methylation levels estimated by the raw signal–based methods Guppy 6.3.8 and Dorado 0.5.3, all 14 of 14 ICRs exhibited a median methylation level within the 25% to 75% range (Supplementary Fig. S15 and Supplementary Fig. S16). The trimmed means for the predicted ICR methylation levels were 0.54 for Guppy 6.3.8 and 0.50 for Dorado 0.5.3, with corresponding standard variances of 0.07 for Guppy 6.3.8 and 0.08 for Dorado 0.5.3. These results demonstrate that the DNA methylation levels of ICR predicted by NanoFreeLunch are concentrated in the middle segment of the [0,1] range, exhibiting mean and variance characteristics consistent with those of raw signal–based methodologies.

### DNA methylation levels estimated by NanoFreeLunch are consistent with histone modification and DNase sensitivity

**Figure 5:**
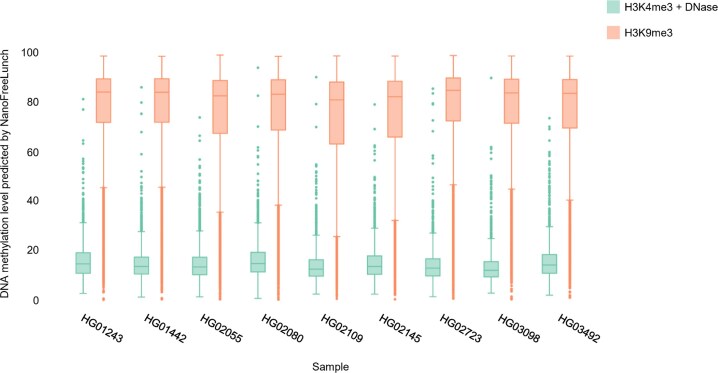
Comparing DNA methylation level predicted by NanoFreeLunch with other epigenetic markers. Each boxplot depicts the distribution of average DNA methylation levels in H3K9me3 regions or DNase-hypersensitive regions marked by H3K4me3, predicted by NanoFreeLunch using human pangenome data. Different colors represent distinct regions. The line in each box represents the median. The lower and upper bounds of the box correspond to the first (Q1) and third (Q3) quartiles, respectively. The lower fence is determined as the last sample point below 1.5 times the interquartile range (IQR), calculated as Q3 minus Q1. Similarly, the upper fence is identified as the last sample point above 1.5 times the IQR. The histone modification and DNase sensitivity are obtained from ENCODE [37, 38]. The basecalling results of Guppy 6.3.8 are used as the input of NanoFreeLunch.

To further evaluate the reliability of NanoFreeLunch, we calculated the consistency between the DNA methylation level predicted by NanoFreeLunch and other epigenomic markers, including histone modification and DNase sensitivity, obtained from the ENCODE project [37, 38] (Methods). We obtained the average CpG methylation level predicted by NanoFreeLunch for H3K9me3 regions and the overlaps between H3K4me3 regions and DNase-hypersensitive regions (Methods). H3K9me3 is the histone mark of regions with repressed transcription and DNA hypermethylation, while H3K4me3 and DNase hypersensitivity are associated with activated transcription and DNA hypomethylation [39]. In the HPGP dataset, which was sequenced using the R9.4.1 flowcell, the DNase-hypersensitive regions marked by H3K4me3 exhibited low methylation levels, as predicted by NanoFreeLunch. The average median methylation levels for these regions were 13.4%, 6.6%, and 8.6% when the data were basecalled by Guppy versions 6.3.8, 4.2.2, and 2.3.5, respectively. In contrast, the H3K9me3 regions exhibited high methylation levels. Specifically, the average median methylation levels for these regions were 83.7%, 86.1%, and 83.9%, respectively (Fig. 5, Supplementary Fig. S17, and Supplementary Fig. S18). NanoFreeLunch-predicted methylation levels are also consistent with those estimated by the raw signal–based method Guppy (Supplementary Fig. S19). According to the Ashkenazim trio data, which were sequenced by the R10.4.1 flowcell, the DNase-hypersensitive regions with H3K4me3 had an average median NanoFreeLunch-predicted methylation level of 2.5%, and the H3K9me3 regions had an average median methylation level of 88.3% (Supplementary Fig. S20), consistent with the Dorado-predicted methylation levels (Supplementary Fig. S21). These results demonstrate that DNA methylation levels estimated by NanoFreeLunch are consistent with histone modification and DNase sensitivity.

## Discussion

Nanopore sequencing provides an unprecedented opportunity for the data mining of DNA methylation data by reutilizing and integrating existing nanopore sequencing data. A major obstacle is that the existing methods for detecting DNA methylation require raw signals from nanopore sequencing as the input, but most data do not include raw signals due to the difficulty of storing, processing, and sharing the raw signal files. In this work, we address this challenge by developing a novel method termed NanoFreeLunch that can detect DNA methylation quantitatively from basecalled FASTQ files without raw signals. With the ability to leverage rapidly accumulating nanopore sequencing data, NanoFreeLunch provides unprecedented opportunities for large-scale construction of epigenomes, even from datasets not originally designed for DNA methylation studies.

This study demonstrated the significance and effectiveness of NanoFreeLunch for detecting hypermethylated, hypomethylated, and partially methylated regions whose methylation status is precisely regulated. These results show that the restored DNA methylation from basecalled FASTQ files when the raw signals are lost is accurate enough to provide biologically meaningful insights.

Major version changes in the basecaller or flowcell might have an impact on the results of NanoFreeLunch since they change the error pattern and QV distribution. NanoFreeLunch should be trained on the matched version to achieve the best accuracy. We provide pretrained models for versions 2.3.5, 4.2.2, and 6.3.8 of Guppy on flowcell R9.4.1 and Dorado 0.5.3 on the R10.4.1 flowcell in this work, but NanoFreeLunch is flexible and has a command line interface (CLI) for users to train their models.

In this study, we used HAC mode for basecalling, which is a commonly used and recommended mode since it has a good balance between speed and accuracy. However, basecalling mode might affect methylation detection of NanoFreeLunch since different modes might have different error and QV patterns. Using models trained on chromosome 10 (HG01109 for R9, HG002 for R10) and tested on chromosome 6 (HG01243 for R9, HG003 for R10), we found that FAST mode yielded slightly higher accuracy than HAC mode, while SUP mode yielded slightly lower accuracy for both R9 and R10 data (Supplementary Fig. S22). This observed dependency, coupled with the complex and nonmonotonic relationship between basecalling accuracy and NanoFreeLunch accuracy, indicates that the specific error and QV characteristics introduced by different basecalling modes may have complex and hard-to-interpret effects on the features used by NanoFreeLunch for methylation prediction.

The method presented in this study offers a versatile and adaptable framework with potential for expansion. While the focus of this work was on detecting 5mC in the CpG context, the same framework can be applied to other types of base modifications by using different training data. NanoFreeLunch provides a CLI for training models with customized data, allowing further study to extend its ability to detect various types of base modifications beyond 5mC.

## Conclusions

NanoFreeLunch offers a distinct solution to a significant challenge in the field: the reutilization of nanopore sequencing data for DNA methylation detection, particularly in the absence of raw signal files in public databases. By introducing a novel strategy that accounts for sequencing error and base QV, NanoFreeLunch enables reliable quantitative detection of DNA methylation. This new method opens avenues for uncovering novel biological insights through large-scale integration of nanopore sequencing for DNA methylation detection.

## Methods

### Extracting the features used by NanoFreeLunch

Assuming that there are *n* reads fully covering the [−10, 10] regions of a genomic locus of interest (Fig. 2), we denote ${R}_{ij}$ as the sequenced base of read $j$ at genomic locus $i$, ${T}_i$ as the reference genome base at locus $i$, and ${Q}_{ij}$ as the base QV of read $j$ at locus $i$, where $i = - 10,\ldots,10$, $j = 1,\ldots,n$. The mean QV vector is defined as


\begin{eqnarray*}
\textit{mean}\left( Q \right) = {\left[ {{M}_i} \right]}_{i\in\left[ { - 10,10} \right]}
\end{eqnarray*}

where ${M}_i = \frac{1}{n}\sum_{j = 1}^n {Q}_{ij}$, and $mean( Q )$ is an $1\ \times \ 21$ row vector. The QV covariance matrix is defined as


\begin{eqnarray*}
cov\left( Q \right) = {\left[ {{V}_{pq}} \right]}_{p,q\in\left[ { - 10,10} \right]}
\end{eqnarray*}

where ${V}_{pq} = \frac{1}{{n - 1}}\sum_{j = 1}^n ( {{Q}_{pj} - {M}_p} )( {{Q}_{qj} - {M}_q} )$, and $cov( Q )$ is an $21\ \times \ 21$ matrix. The QV coskewness matrix is defined as


\begin{eqnarray*}
\textit{coskewness}\left( Q \right) = {\left[ {{S}_{pq}} \right]}_{p,q\in\left[ { - 10,10} \right]}
\end{eqnarray*}

where ${S}_{pq} = \frac{{\sum_{j = 1}^n {{( {{Q}_{pj} - {M}_p} )}}^2( {{Q}_{qj} - {M}_q} )}}{{n\sigma _p^2{\sigma }_q}}$, and ${\sigma }_p$ and ${\sigma }_q$ are standard variances. $coskewness( Q )$ is an $21\ \times \ 21$ matrix. The QV cokurtosis matrix is defined as


\begin{eqnarray*}
\textit{coskurtosis}\left( Q \right) = {\left[ {{K}_{pq}} \right]}_{p,q\in\left[ { - 10,10} \right]}
\end{eqnarray*}

where ${K}_{pq} = \frac{{\sum_{j = 1}^n {{( {{Q}_{pj} - {M}_p} )}}^2{{( {{Q}_{qj} - {M}_q} )}}^2}}{{n\sigma _p^2\sigma _q^2}}$, ${\sigma }_p$ and ${\sigma }_q$ are standard variances. $coskurtosis( Q )$ is an $21\ \times \ 21$ matrix. The pairwise joint sequencing error rate is defined as


\begin{eqnarray*}
\textit{error}\left( R \right) = {\left[ {{E}_{\textit{stpq}}} \right]}_{s,t\in\left[ {A,C,G,T,D} \right],p,q\in\left[ { - 10,10} \right]}
\end{eqnarray*}

where ${E}_{\textit{stpq}} = \frac{1}{n}\sum_{j = 1}^n I( {{R}_{pj} = s} )I( {{R}_{qj} = t} )I( {{T}_p\ne s} )I( {{T}_q\ne t} )$, $I( \cdot )$ is the indicator function, D is the deletion, and $error( R )$ is a $5\ \times \ 5\ \times \ 21\ \times \ 21$ four-dimensional array. The sequence context is binary coded with 2 digits for each base as follows:


\begin{eqnarray*}
\textit{context} = {\left[ {{C}_i} \right]}_{i\in\left[ { - 10,10} \right]}
\end{eqnarray*}

where *C_i_* = 00 if *T_i_* = “A,” *C_i_* = 01 if *T_i_* = “C,” *C_i_* = 10 if *T_i_* = “G,” and *C_i_* = 11 if *T_i_* = “T.”

### Data preparation and preprocessing

The FAST5 files of R9 (abbreviated as R9.4.1 flowcell) data were downloaded from [29] and [30] for the HG002 dataset and the HPGP dataset, respectively. The POD5/FAST5 files of the R10.4.1 flowcell (abbreviated as R10) Ashkenazim trio data are downloaded from [32] and [40] for the 4-kHz and 5-kHz flowcells, respectively. For the R9 data, basecalling was performed using the parameters “*guppy_basecaller -x "cuda:all" –compress_fastq –bam_out -r -c dna_r9.4.1_450bps_hac.cfg*,” and base-level CpG methylation calling was performed using “*guppy_basecaller -x "cuda:all" –compress_fastq –bam_out -r -c dna_​r9.4.1_​450bps_​modbases_​5mc_​cg_​hac.​cfg*” with Guppy 6.3.8. For the R10 data, basecalling was performed using the following parameters: “dorado basecaller –reference reffile hac infile > bamfile,” and base-level CpG methylation calling was performed using “dorado basecaller –reference reffile hac,5mCG_5hmCG infile > bamfile” with Dorado 0.5.3. Reads mapping was performed using Guppy/Dorado along with basecalling by providing GRCh38 obtained from s3://ont-open-data/gm24385_mod_2021.09/refs as the reference genome. DNA methylation calling for genomic loci was performed using modbam2bed (version 0.6.3) downloaded from [41] with the parameters “modbam2bed -e -m 5mC –cpg.” Basecalling results obtained using Guppy 2.3.5 and Guppy 4.2.2 were downloaded from [30] for 7 samples—HG01109, HG01243, HG02055, HG02080, HG02723, HG03098, and HG03492—and the other samples did not include basecalling results obtained with Guppy 2.3.5 or Guppy 4.2.2. The basecalled reads were mapped to GRCh38 using minimap2 [42] (version 2.24) with the parameters “minimap2 -ax map-ont –secondary=no –sam-hit-only -L,” and the mapped reads were sorted, indexed, and filtered using samtools [43] (version 1.16.1) with the parameters “samtools sort,” “samtools index,” and “samtools view -h -b -F 4079.” The methylation calling results of whole-genome bisulfite sequencing used as the benchmark in this study were downloaded from EPI2ME Desktop (s3://​ont-open-data/​gm24385_mod_2021.09/​bisulphite/​cpg/​CpG​.gz.​bismark.​zero.cov.gz).

### Training the model of NanoFreeLunch

All the analyses for detecting DNA methylation without raw signals used version 0.24.0 of NanoFreeLunch. For the R9 data, we used chromosome 10 of sample HG01109 from the HPGP dataset and sample HG002 from ONT Open Data as the training data for the R9 PromethION data and R9 MinION data, respectively. For the R10 data, we used chromosome 10 of sample HG002 from ONT Open Data for the R10 4-kHz and R10 5-kHz data. The mapped reads were converted to the features described in the section on extracting the features used by NanoFreeLunch by “*nfl prepdata -r -p -f –chr chrname bamfile reffile locifile*,” where *bamfile* is the mapped reads, *reffile* is the FASTA file of the reference genome, and *locifile* is the CpG loci reported by modbam2bed. In the *locifile*, loci with depth lower than 10× or score less than 800 were removed. This command also converts matrices and high-dimensional arrays to vectors so that they can be used as the input of the gradient boosting model. The DNA methylation level at each CpG locus predicted by modbam2bed was logit-transformed with the following formula and used as the response of the model:


\begin{eqnarray*}
y^{\prime} = \left\{ {\begin{array}{@{}*{1}{c}@{}} {- \alpha, \quad\quad\quad y = 0}\\ {\log \left( y \right) - log \left( {1 - y} \right), \quad 0 < y < 1}\\ {\alpha, \quad y = 1} \end{array}} \right.
\end{eqnarray*}

where $\alpha = {10}^{ - 3}$ in this study. The model is trained with the parameters “nfl train –alpha 1e-3.” The core gradient boosting model is implemented by the Julia [44] wrapper of XGBoost (version 1.5.2) [25, 45]. The learning rate, “*eta*,” is 0.1; the number of trees, “*num_round*,” is 1,500; the maximal tree depth, “*max_depth*,” is 8; and the other parameters are set to their defaults.

### Estimating DNA methylation levels

We used the trained model to predict the DNA methylation level by the command “nfl predict” with default parameters. This NanoFreeLunch command internally calls the trained XGBoost model with the input features. The DNA methylation levels in the forward and backward strands of the same CpG site were averaged.

### Estimating the DNA methylation level of CpG islands

The GRCh38-based genomic coordinates of CpG islands were downloaded from the UCSC genome browser [46] by selecting “Regulation” in “Group,” “CpG islands” in “Track,” and “GRCh38” in “Assembly.” The average methylation level of a CpG island is estimated by the trimmed mean of the CpG methylation level by removing data points outside of the [median − variance, median + variance] range. The command is “*nfl get-range-trimmean -f*.”

### Estimating the DNA methylation level of ICRs

The ICRs are obtained from regions in Table 1 of Jima et al. [36] with a “#” mark and filtered by retaining the regions that can also be found in Table 1 of Skaar et al. [35]. The genomic coordinates in Skaar et al. [35] are based on GRCh37, and we used LiftOver [47] to convert them to GRCh38-based coordinates. Similar to the method described in the section on estimating DNA methylation level of CpG islands, the DNA methylation levels of the ICRs are estimated by the “*nfl get-range-trimmean -f*.” The mean and variance of ICR methylation levels are calculated using trimmed statistics, specifically by excluding the highest and lowest 5% of data points.

### Estimating DNA methylation levels in regions with different histone marks

We used the histone mark data and DNase sensitivity data of GM12878 from the ENCODE project [37, 38] as the reference epigenome in this study. The H3K4me3 peak regions were downloaded from [48]. The H3K9me3 peak regions were downloaded from [49]. The DNase-hypersensitive regions were downloaded from [50]. “H3K4m3 +​ DNase” regions are the overlapping regions between H3K4me3 peak regions and DNase-hypersensitive regions. Similar to the method described in the section on estimating DNA methylation level of CpG islands, the average methylation level of a region is estimated by “*nfl get-range-trimmean-f*.”

### Assessing the relative importance of different features

We used the region 50,000,000–60,000,000 on chromosome 10 of sample HG01109 to assess the impact of DNA methylation on these features. The features are extracted from the aligned reads, as described in the section on extracting the features used by NanoFreeLunch, for the 106,209 CpG loci in this region. The loci with DNA methylation levels predicted by Guppy less than 0.1 and greater than 0.9 were regarded as unmethylated loci and methylated loci, respectively. The differences between the average features of methylated loci and unmethylated loci are shown in Supplementary Fig. S1. The model accuracy using each feature or combination of features was evaluated using chromosome 6 of the 9 samples from the Human Pangenome Project. The average accuracies are shown in Supplementary Fig. S2.

### Statistics of the SRA records

We performed an advanced search of the SRA database [20] and selected “oxford nanopore” in “Platform” to obtain the total number of ONT records, denoted as ${N}_{\textit{total}}$. Similarly, we selected “oxford nanopore” in “Platform” and “filetype nanopore” in “Properties” to obtain the number of ONT records with the raw FAST5/POD5 files, denoted as ${N}_{raw}$. The proportion of records consisting of raw FAST5/POD5 files was calculated by ${N}_{raw}/{N}_{\textit{total}}$. To obtain the statistics for each year in Fig. 1 , we repeated the process by setting “Publication Date,” ranging from 2015 to 2023.

## Availability of Source Code and Requirements

Project name: NanoFreeLunch

Project homepage: https://gitee.com/zhixingfeng/NanoFreeLunch.jl [51]

Project demo: https://gitee.com/zhixingfeng/nfl-demo/tree/main/demo [52]

biotoolsID: nanofreelunch

RRID:SCR_027196

Operating system(s): Linux for x86_64 machines.

Programming languages: Julia

License: GNU GPL v3

## Additional Files

**Supplementary Fig. S1**. The impact of 5mC on different features. The y-axis shows the differences in the features between the methylated loci and unmethylated loci. (A) Mean of QV. (B) Covariance of QV. (C) Coskewness of QV. (D) Cokurtosis of QV. (E) Pairwise joint error rates.

**Supplementary Fig. S2**. The accuracy of NanoFreeLunch using different features. The accuracy is the Pearson correlation coefficient between the DNA methylation level predicted by NanoFreeLunch and Guppy 6.3.8 on chromosome 6 of the human pangenome data.

**Supplementary Fig. S3**. The accuracy of NanoFreeLunch using Guppy 6.3.8 for basecalling on the human pangenome data. The x-axis and y-axis are the DNA methylation levels of each CpG site predicted by Guppy and NanoFreeLunch, respectively. Predicted DNA methylation levels are segmented into 20 bins of equal size ranging from 0% to 100%. The color of each bin represents the base-10 logarithm transformation of the number of loci within the bin. PCC denotes Pearson correlation coefficient. (A–I) The results for each sample.

**Supplementary Fig. S4**. The accuracy of NanoFreeLunch using Guppy 4.2.2 for basecalling on the human pangenome data. The x-axis and y-axis are the DNA methylation levels of each CpG site predicted by Guppy and NanoFreeLunch, respectively. Predicted DNA methylation levels are segmented into 20 bins of equal size ranging from 0% to 100%. The color of each bin represents the base-10 logarithm transformation of the number of loci within the bin. PCC denotes Pearson correlation coefficient. (A–F) The results for each sample.

**Supplementary Fig. S5**. The accuracy of NanoFreeLunch using Guppy 2.3.5 for basecalling on the human pangenome data. The x-axis and y-axis are the DNA methylation levels of each CpG site predicted by Guppy and NanoFreeLunch, respectively. Predicted DNA methylation levels are segmented into 20 bins of equal size ranging from 0% to 100%. The color of each bin represents the base-10 logarithm transformation of the number of loci within the bin. PCC denotes Pearson correlation coefficient. (A–F) The results for each sample.

**Supplementary Fig. S6**. The accuracy of NanoFreeLunch using Dorado 0.5.3 for basecalling on the Ashkenazim trio data. The x-axis and y-axis are the DNA methylation levels of each CpG site predicted by Dorado and NanoFreeLunch, respectively. Predicted DNA methylation levels are segmented into 20 bins of equal size ranging from 0% to 100%. The color of each bin represents the base-10 logarithm transformation of the number of loci within the bin. PCC denotes Pearson correlation coefficient. (A–C) The results for the 4-kHz data. (D–F) The results for the 5-kHz data.

**Supplementary Fig. S7**. The region-level accuracy of NanoFreeLunch using Guppy 6.3.8 for basecalling on the human pangenome data. The x-axis and y-axis are the average DNA methylation levels of each CpG island predicted by Guppy and NanoFreeLunch, respectively. Predicted DNA methylation levels are segmented into 20 bins of equal size ranging from 0% to 100%. The color of each bin represents the base-10 logarithm transformation of the number of CpG islands within the bin. PCC denotes Pearson correlation coefficient. (A–I) The results for each sample.

**Supplementary Fig. S8**. The region-level accuracy of NanoFreeLunch using Guppy 4.2.2 for basecalling on the human pangenome data. The x-axis and y-axis are the average DNA methylation levels of each CpG island predicted by Guppy and NanoFreeLunch, respectively. Predicted DNA methylation levels are segmented into 20 bins of equal size ranging from 0% to 100%. The color of each bin represents the base-10 logarithm transformation of the number of CpG islands within the bin. PCC denotes Pearson correlation coefficient. (A–F) The results for each sample.

**Supplementary Fig. S9**. The region-level accuracy of NanoFreeLunch using Guppy 2.3.5 for basecalling on the human pangenome data. The x-axis and y-axis are the average DNA methylation levels of each CpG island predicted by Guppy and NanoFreeLunch, respectively. Predicted DNA methylation levels are segmented into 20 bins of equal size ranging from 0% to 100%. The color of each bin represents the base-10 logarithm transformation of the number of CpG islands within the bin. PCC denotes Pearson correlation coefficient. (A–F) The results for each sample.

**Supplementary Fig. S10**. The region-level accuracy of NanoFreeLunch using Dorado 0.5.3 for basecalling on the Ashkenazim trio data. The x-axis and y-axis are the average DNA methylation levels of each CpG island predicted by Guppy and NanoFreeLunch, respectively. Predicted DNA methylation levels are segmented into 20 bins of equal size ranging from 0% to 100%. The color of each bin represents the base-10 logarithm transformation of the number of CpG islands within the bin. PCC denotes Pearson correlation coefficient. (A–C) The results for the 4-kHz data. (D–F) The results for the 5-kHz data.

**Supplementary Fig. S11**. The accuracy of NanoFreeLunch using the data obtained by different flowcell types and basecallers. PCC represents Pearson correlation coefficient. The PCC in the figure is the average PCC of each category.

**Supplementary Fig. S12**. The average DNA methylation level of ICR predicted by NanoFreeLunch using Guppy 2.3.5 for basecalling. Each boxplot illustrates the distribution of the average DNA methylation levels of ICRs predicted by NanoFreeLunch using human pangenome data. The line in each box represents the median. The lower and upper bounds of the box correspond to the first (Q1) and third (Q3) quartiles, respectively. The lower fence is determined as the last sample point below 1.5 times the interquartile range (IQR), calculated as Q3 minus Q1. Similarly, the upper fence is identified as the last sample point above 1.5 times the IQR. On the x-axis, the region on the left of “→” is the genomic region of the ICR on GRCh38, and the gene symbol on the right is the putative gene associated with the ICR. The basecalling results of Guppy 2.3.5 are used as the input of NanoFreeLunch.

**Supplementary Fig. S13**. The average DNA methylation level of ICR predicted by NanoFreeLunch using Guppy 4.2.2 for basecalling. Each boxplot illustrates the distribution of the average DNA methylation levels of ICRs predicted by NanoFreeLunch using human pangenome data. The line in each box represents the median. The lower and upper bounds of the box correspond to the first (Q1) and third (Q3) quartiles, respectively. The lower fence is determined as the last sample point below 1.5 times the interquartile range (IQR), calculated as Q3 minus Q1. Similarly, the upper fence is identified as the last sample point above 1.5 times the IQR. On the x-axis, the region on the left of “→” is the genomic region of the ICR on GRCh38, and the gene symbol on the right is the putative gene associated with the ICR. The basecalling results of Guppy 4.2.2 are used as the input of NanoFreeLunch.

**Supplementary Fig. S14**. The average DNA methylation level of ICR predicted by NanoFreeLunch using Dorado 0.5.3 for basecalling. Each boxplot illustrates the distribution of the average DNA methylation levels of ICRs predicted by NanoFreeLunch using the R10 Ashkenazim trio data. The line in each box represents the median. The lower and upper bounds of the box correspond to the first (Q1) and third (Q3) quartiles, respectively. The lower fence is determined as the last sample point below 1.5 times the interquartile range (IQR), calculated as Q3 minus Q1. Similarly, the upper fence is identified as the last sample point above 1.5 times the IQR. On the x-axis, the region on the left of “→” is the genomic region of the ICR on GRCh38, and the gene symbol on the right is the putative gene associated with the ICR. The basecalling results of Dorado 0.5.3 are used as the input of NanoFreeLunch.

**Supplementary Fig. S15**. The average DNA methylation level of ICR predicted by Guppy 6.3.8. Each boxplot illustrates the distribution of the average DNA methylation levels of ICRs predicted by Guppy using human pangenome data. The line in each box represents the median. The lower and upper bounds of the box correspond to the first (Q1) and third (Q3) quartiles, respectively. The lower fence is determined as the last sample point below 1.5 times the interquartile range (IQR), calculated as Q3 minus Q1. Similarly, the upper fence is identified as the last sample point above 1.5 times the IQR. On the x-axis, the region on the left of “→” is the genomic region of the ICR on GRCh38, and the gene symbol on the right is the putative gene associated with the ICR.

**Supplementary Fig. S16**. The average DNA methylation level of ICR predicted by Dorado 0.5.3. Each boxplot illustrates the distribution of the average DNA methylation levels of ICRs predicted by Dorado using the R10 Ashkenazim trio data. The line in each box represents the median. The lower and upper bounds of the box correspond to the first (Q1) and third (Q3) quartiles, respectively. The lower fence is determined as the last sample point below 1.5 times the interquartile range (IQR), calculated as Q3 minus Q1. Similarly, the upper fence is identified as the last sample point above 1.5 times the IQR. On the x-axis, the region on the left of “→” is the genomic region of the ICR on GRCh38, and the gene symbol on the right is the putative gene associated with the ICR.

**Supplementary Fig. S17**. Comparing DNA methylation level predicted by NanoFreeLunch using Guppy 4.2.2 for basecalling with other epigenetic markers. Each boxplot depicts the distribution of average DNA methylation levels in H3K9me3 regions or DNase-hypersensitive regions marked by H3K4me3, predicted by NanoFreeLunch using human pangenome data. Different colors represent distinct regions. The line in each box represents the median. The lower and upper bounds of the box correspond to the first (Q1) and third (Q3) quartiles, respectively. The lower fence is determined as the last sample point below 1.5 times the interquartile range (IQR), calculated as Q3 minus Q1. Similarly, the upper fence is identified as the last sample point above 1.5 times the IQR. The histone modification data and DNase data are obtained from the GM12878 cell line of ENCODE. The basecalling results of Guppy 4.2.2 are used as the input of NanoFreeLunch.

**Supplementary Fig. S18**. Comparing DNA methylation level predicted by NanoFreeLunch using Guppy 2.3.5 for basecalling with other epigenetic markers. Each boxplot depicts the distribution of average DNA methylation levels in H3K9me3 regions or DNase-hypersensitive regions marked by H3K4me3, predicted by NanoFreeLunch using human pangenome data. Different colors represent distinct regions. The line in each box represents the median. The lower and upper bounds of the box correspond to the first (Q1) and third (Q3) quartiles, respectively. The lower fence is determined as the last sample point below 1.5 times the interquartile range (IQR), calculated as Q3 minus Q1. Similarly, the upper fence is identified as the last sample point above 1.5 times the IQR. The histone modification data and DNase data are obtained from the GM12878 cell line of ENCODE. The basecalling results of Guppy 2.3.5 are used as the input of NanoFreeLunch.

**Supplementary Fig. S19**. The average DNA methylation level of regions with different epigenetic markers predicted by Guppy 6.3.8. Each boxplot depicts the distribution of average DNA methylation levels in H3K9me3 regions or DNase-hypersensitive regions marked by H3K4me3, predicted by Guppy using human pangenome data. Different colors represent distinct regions. The line in each box represents the median. The lower and upper bounds of the box correspond to the first (Q1) and third (Q3) quartiles, respectively. The lower fence is determined as the last sample point below 1.5 times the interquartile range (IQR), calculated as Q3 minus Q1. Similarly, the upper fence is identified as the last sample point above 1.5 times the IQR. The histone modification data and DNase data are obtained from the GM12878 cell line of ENCODE.

**Supplementary Fig. S20**. Comparing DNA methylation level predicted by NanoFreeLunch using Dorado 0.5.3 for basecalling with other epigenetic markers. Each boxplot depicts the distribution of average DNA methylation levels in H3K9me3 regions or DNase-hypersensitive regions marked by H3K4me3, predicted by NanoFreeLunch using the R10 Ashkenazim trio data. Different colors represent distinct regions. The line in each box represents the median. The lower and upper bounds of the box correspond to the first (Q1) and third (Q3) quartiles, respectively. The lower fence is determined as the last sample point below 1.5 times the interquartile range (IQR), calculated as Q3 minus Q1. Similarly, the upper fence is identified as the last sample point above 1.5 times the IQR. The histone modification data and DNase data are obtained from the GM12878 cell line of ENCODE. The basecalling results of Dorado 0.5.3 are used as the input of NanoFreeLunch.

**Supplementary Fig. S21**. The average DNA methylation level of regions with different epigenetic markers predicted by Dorado 0.5.3. Each boxplot depicts the distribution of average DNA methylation levels in H3K9me3 regions or DNase-hypersensitive regions marked by H3K4me3, predicted by Dorado using the R10 Ashkenazim trio data. Different colors represent distinct regions. The line in each box represents the median. The lower and upper bounds of the box correspond to the first (Q1) and third (Q3) quartiles, respectively. The lower fence is determined as the last sample point below 1.5 times the interquartile range (IQR), calculated as Q3 minus Q1. Similarly, the upper fence is identified as the last sample point above 1.5 times the IQR. The histone modification data and DNase data are obtained from the GM12878 cell line of ENCODE.

**Supplementary Fig. S22**. The impact of basecalling mode on the accuracy of NanoFreeLunch. Comparison of NanoFreeLunch performance using R9 (Guppy) and R10 (Dorado) data basecalled with FAST, HAC, and SUP modes. Bars show the Pearson correlation coefficient (PCC) between methylation levels predicted by NanoFreeLunch and those predicted by the respective basecaller.

giaf113_supplementary_figures

giaf113_Authors_Response_To_Reviewer_Comments_Original_Submission

giaf113_GIGA-D-25-00019_Original_Submission

giaf113_GIGA-D-25-00019_Revision_1

giaf113_Reviewer_1_Report_Original_Submission Callum MacPhillamy, Ph.D. -- 2/9/2025

giaf113_Reviewer_2_Report_Original_Submission Simon Heath -- 4/2/2025

giaf113_Reviewer_2_Report_Revision_1 Simon Heath -- 8/5/2025

## Abbreviations

5hmC: 5-hydroxymethylcytosine; 5mC: 5-methylcytosine; CLI: command line interface; HPGP: Human Pangenome Project; ICR: imprinting control region; NGS: next-generation sequencing; PCC: Pearson correlation coefficient; QV: quality value; SRA: Sequence Read Archive.

## Data Availability

The ONT open dataset’s MinION R9.4.1 flowcell data were obtained from EPI2ME Desktop [29]. The PromethION R9.4.1 flowcell data released by the Human Pangenome Project were obtained from GitHub [30]. The ONT open dataset’s PromethION R10.4.1 flowcell 4-kHz and 5-kHz data were obtained from EPI2ME Desktop [32] and [40], respectively. The histone mark data and DNase sensitivity data of GM12878 were obtained from the ENCODE project. Specifically, the H3K4me3 peak regions were downloaded from EPI2ME Desktop [48]. The H3K9me3 peak regions were downloaded from EPI2ME Desktop [49]. The DNase-hypersensitive regions were downloaded from EPI2ME Desktop [50]. There are additional demo data hosted in NanoFreeLunch [53].
