# Supplementary material for: Quantitative detection of DNA methylation from nanopore sequencing data without raw signals
Source: Gigascience. 2025 Oct 31;14:giaf113. doi: 10.1093/gigascience/giaf113 (PMC12576052; doi:10.1093/gigascience/giaf113)
Supplement: giaf113_supplementary_figures [file giaf113_supplementary_figures.pdf]

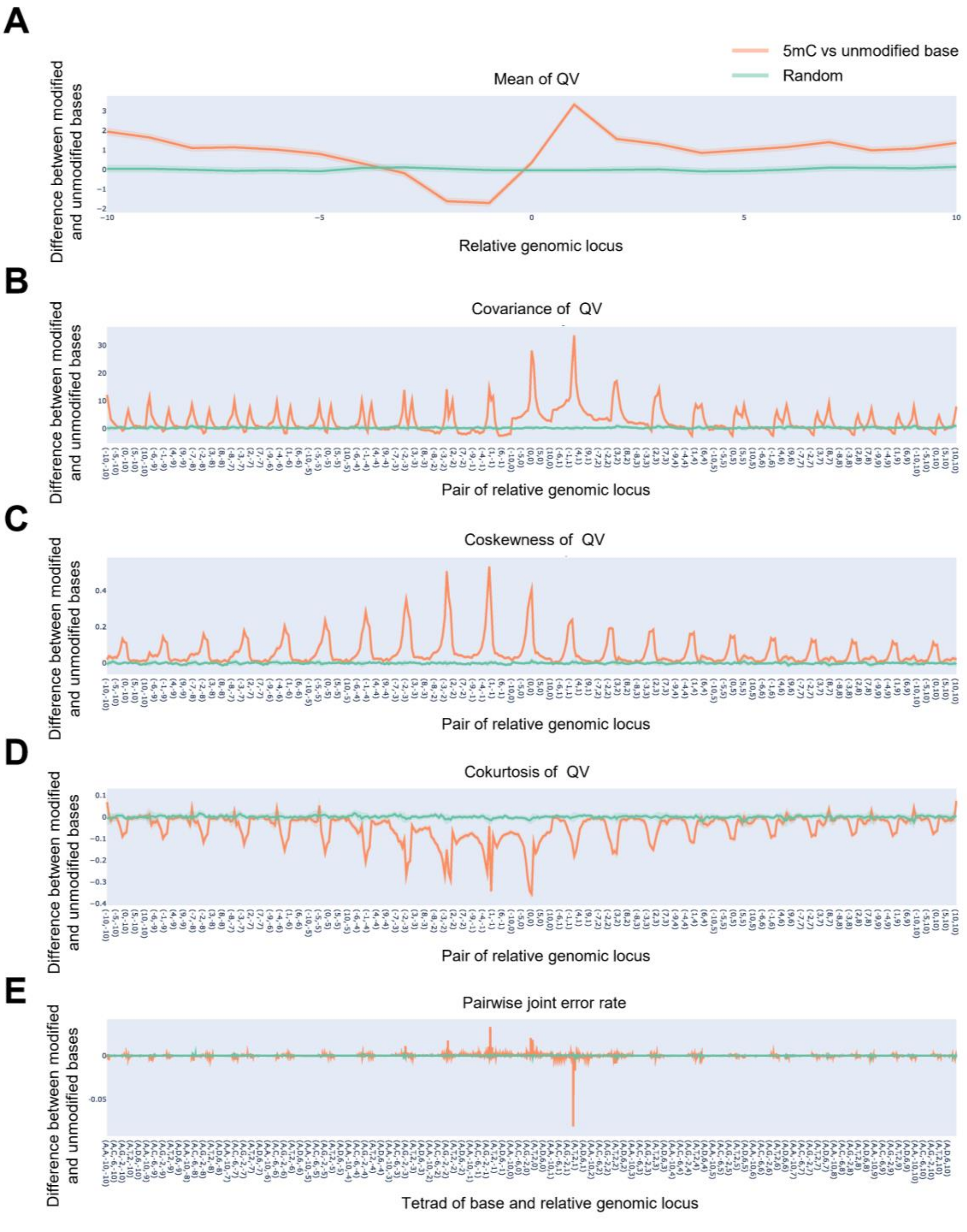

Supplementary Fig. S1. The impact of 5mC on different features. The y-axis shows the differences in the features between the methylated loci and unmethylated loci. **A,** Mean of QV**. B,** Covariance of QV**. C,** Coskewness of QV**. D,** Cokurtosis of QV**. E,** Pairwise joint error rates.

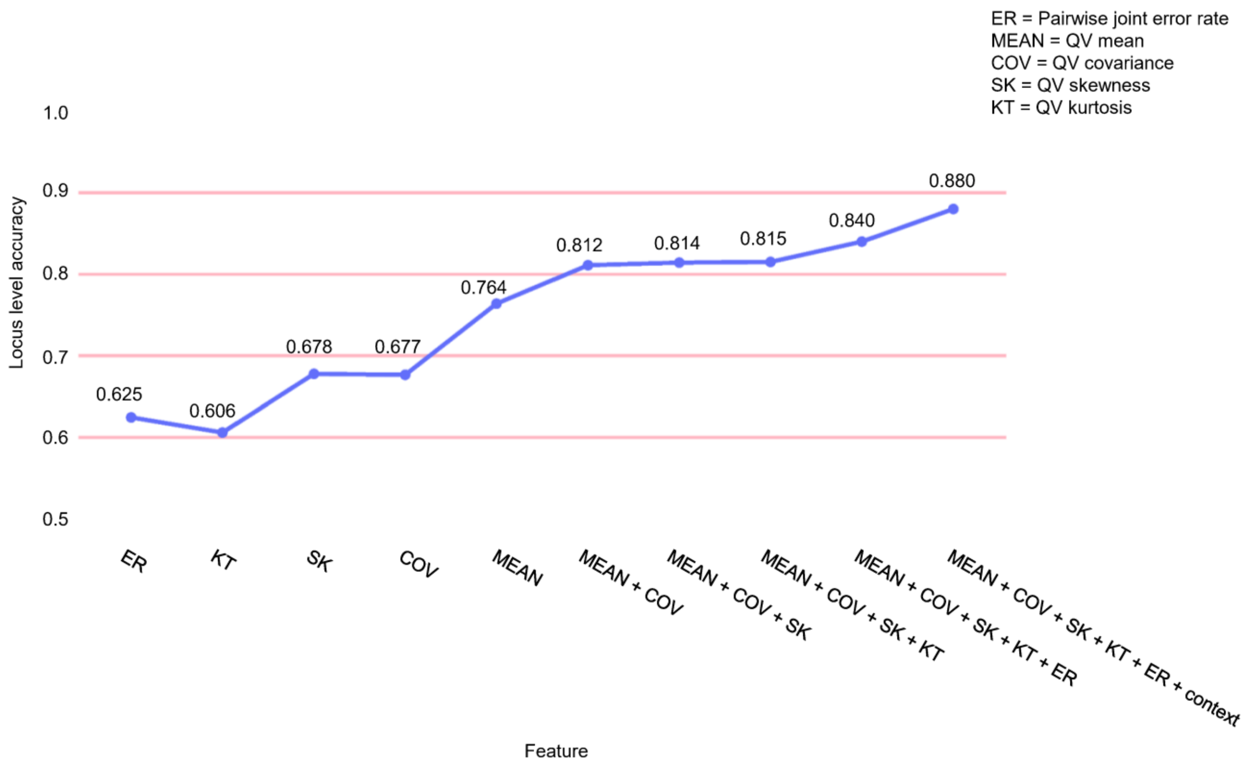

Supplementary Fig. S2. The accuracy of NanoFreeLunch using different features. The accuracy is the Pearson correlation coefficient between the DNA methylation level predicted by NanoFreeLunch and Guppy 6.3.8 on chromosome 6 of the human pangenome data.

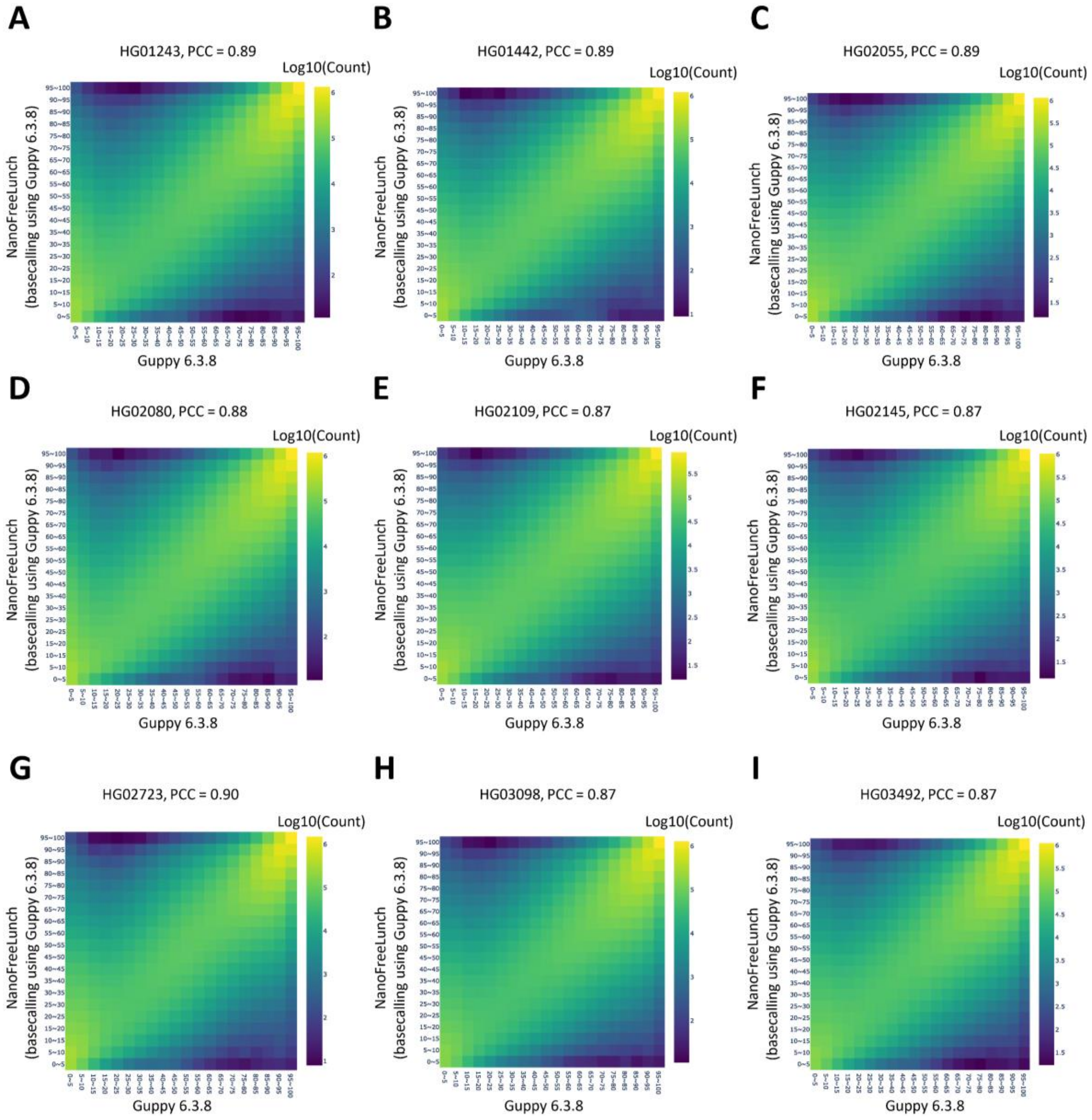

Supplementary Fig. S3. The accuracy of NanoFreeLunch using Guppy 6.3.8 for basecalling on the human pangenome data. The x-axis and y-axis are the DNA methylation levels of each CpG site predicted by Guppy and NanoFreeLunch respectively. Predicted DNA methylation levels are segmented into 20 bins of equal size ranging from 0% to 100%. The color of each bin represents the base-10 logarithm transformation of the number of loci within the bin. PCC denotes Pearson Correlation Coefficient. **A-I,** The results for each sample.

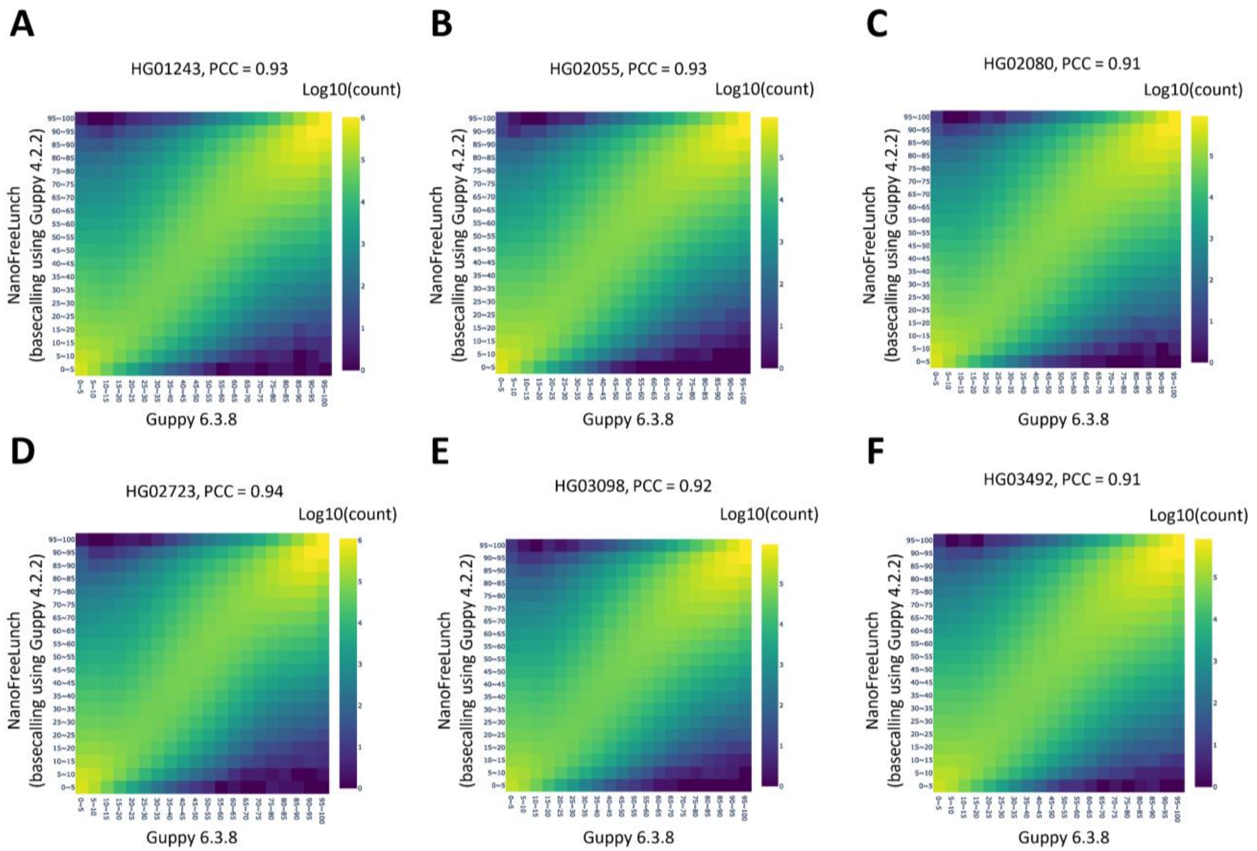

Supplementary Fig. S4. The accuracy of NanoFreeLunch using Guppy 4.2.2 for basecalling on the human pangenome data. The x-axis and y-axis are the DNA methylation levels of each CpG site predicted by Guppy and NanoFreeLunch respectively. Predicted DNA methylation levels are segmented into 20 bins of equal size ranging from 0% to 100%. The color of each bin represents the base-10 logarithm transformation of the number of loci within the bin. PCC denotes Pearson Correlation Coefficient. **A-F,** The results for each sample.

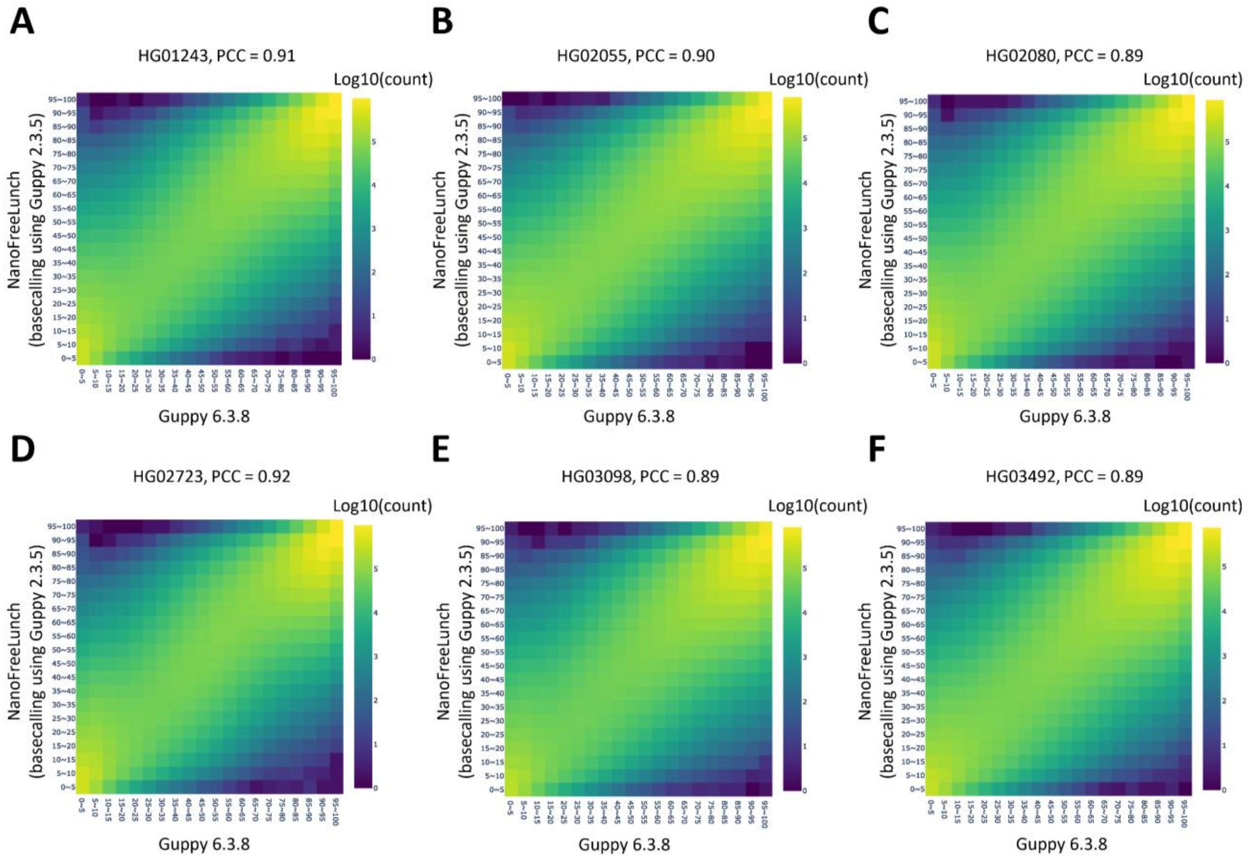

Supplementary Fig. S5. The accuracy of NanoFreeLunch using Guppy 2.3.5 for basecalling on the human pangenome data. The x-axis and y-axis are the DNA methylation levels of each CpG site predicted by Guppy and NanoFreeLunch respectively. Predicted DNA methylation levels are segmented into 20 bins of equal size ranging from 0% to 100%. The color of each bin represents the base-10 logarithm transformation of the number of loci within the bin. PCC denotes Pearson Correlation Coefficient. **A-F,** The results for each sample.

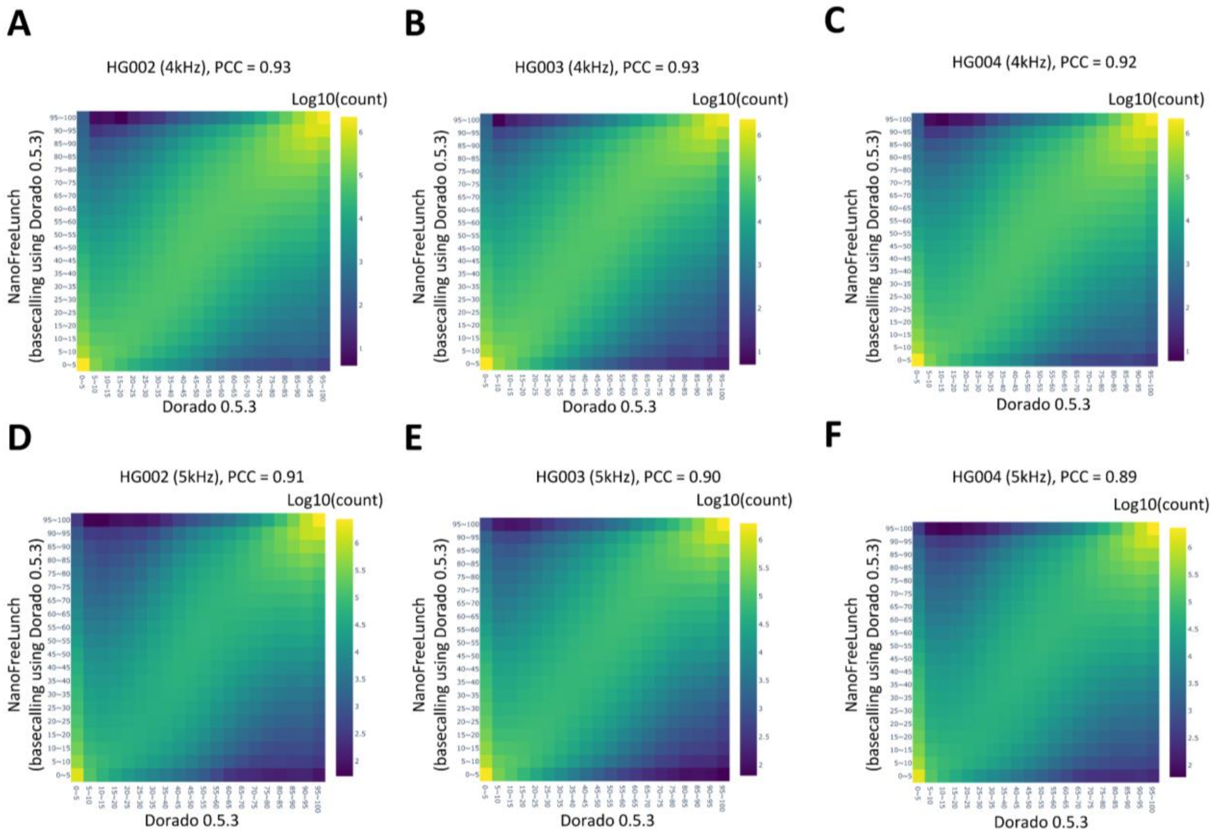

Supplementary Fig. S6. The accuracy of NanoFreeLunch using Dorado 0.5.3 for basecalling on the Ashkenazim Trio data. The x-axis and y-axis are the DNA methylation levels of each CpG site predicted by Dorado and NanoFreeLunch respectively. Predicted DNA methylation levels are segmented into 20 bins of equal size ranging from 0% to 100%. The color of each bin represents the base-10 logarithm transformation of the number of loci within the bin. PCC denotes Pearson Correlation Coefficient. **A-C**, The results for the 4kHz data. **D-F**, The results for the 5kHz data.

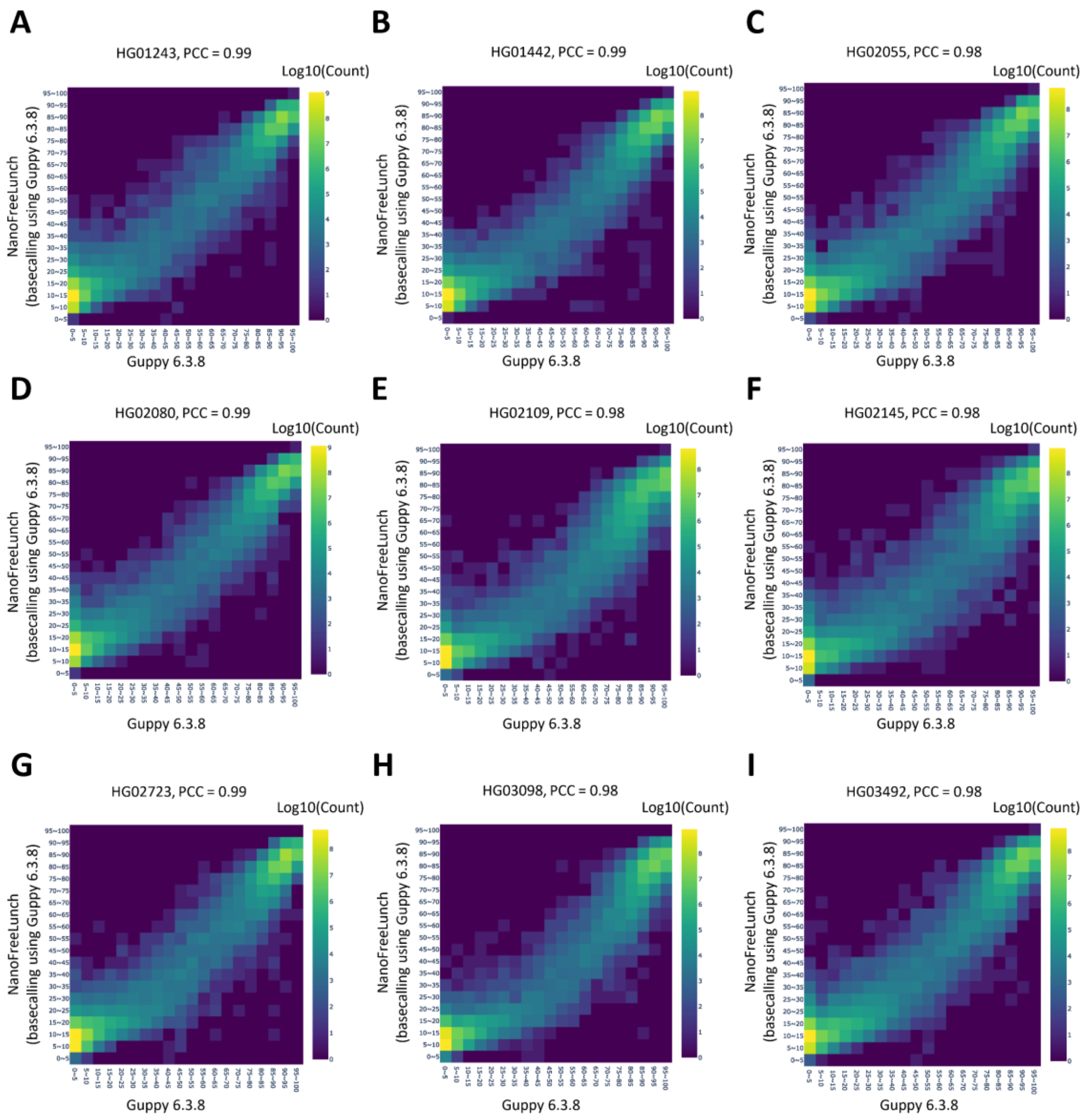

Supplementary Fig. S7. The region-level accuracy of NanoFreeLunch using Guppy 6.3.8 for basecalling on the human pangenome data. The x-axis and y-axis are the average DNA methylation levels of each CpG island predicted by Guppy and NanoFreeLunch respectively. Predicted DNA methylation levels are segmented into 20 bins of equal size ranging from 0% to 100%. The color of each bin represents the base-10 logarithm transformation of the number of CpG islands within the bin. PCC denotes Pearson Correlation Coefficient. **A-I,** The results for each sample.

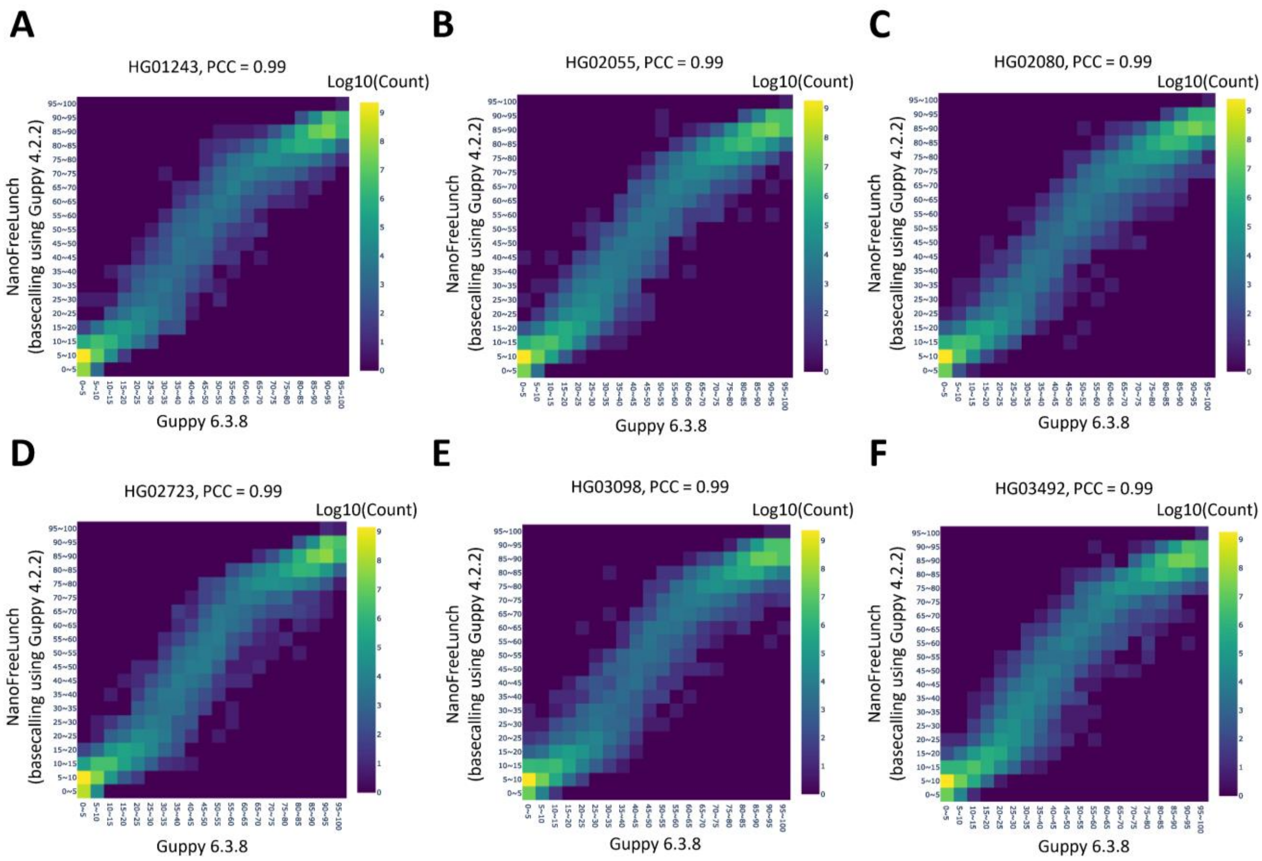

Supplementary Fig. S8. The region-level accuracy of NanoFreeLunch using Guppy 4.2.2 for basecalling on the human pangenome data. The x-axis and y-axis are the average DNA methylation levels of each CpG island predicted by Guppy and NanoFreeLunch respectively. Predicted DNA methylation levels are segmented into 20 bins of equal size ranging from 0% to 100%. The color of each bin represents the base-10 logarithm transformation of the number of CpG islands within the bin. PCC denotes Pearson Correlation Coefficient. **A-F,** The results for each sample.

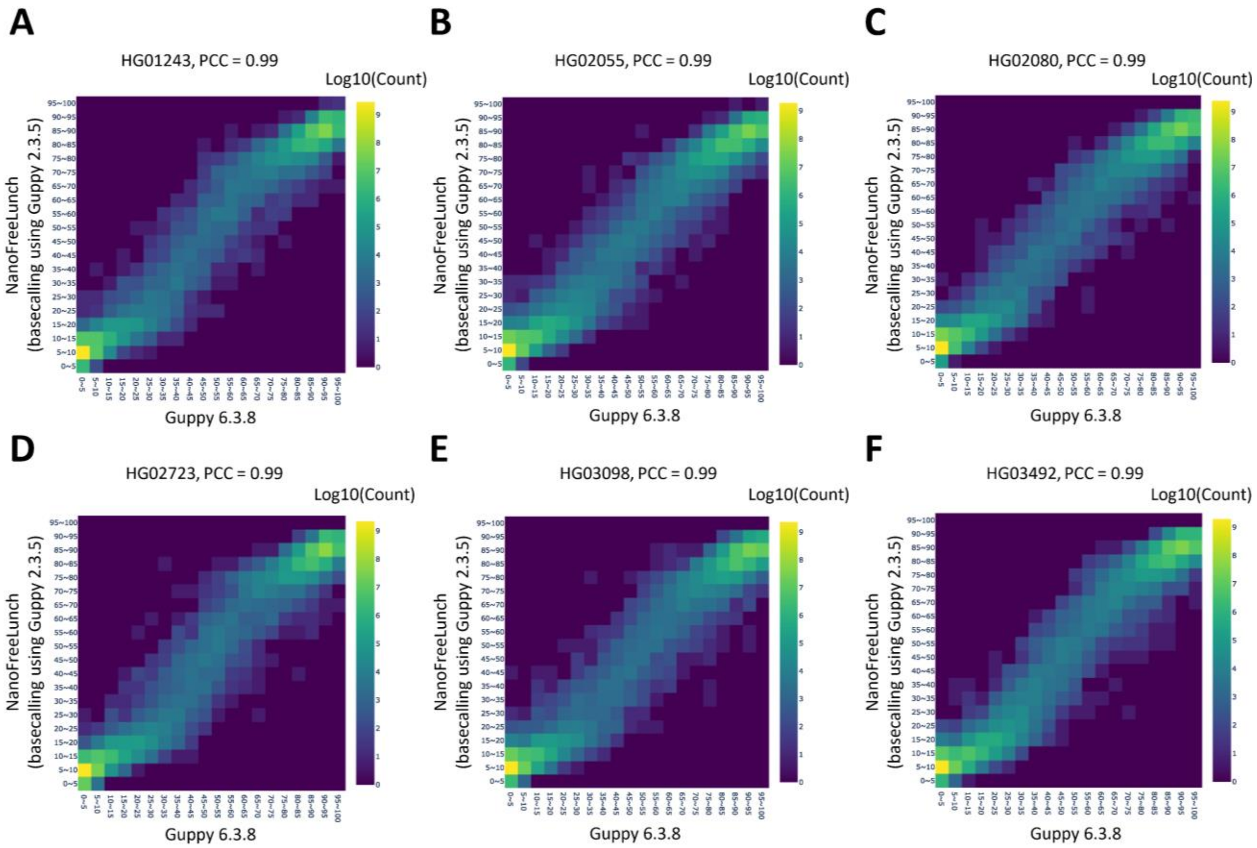

Supplementary Fig. S9. The region-level accuracy of NanoFreeLunch using Guppy 2.3.5 for basecalling on the human pangenome data. The x-axis and y-axis are the average DNA methylation levels of each CpG island predicted by Guppy and NanoFreeLunch respectively. Predicted DNA methylation levels are segmented into 20 bins of equal size ranging from 0% to 100%. The color of each bin represents the base-10 logarithm transformation of the number of CpG islands within the bin. PCC denotes Pearson Correlation Coefficient. **A-F,** The results for each sample.

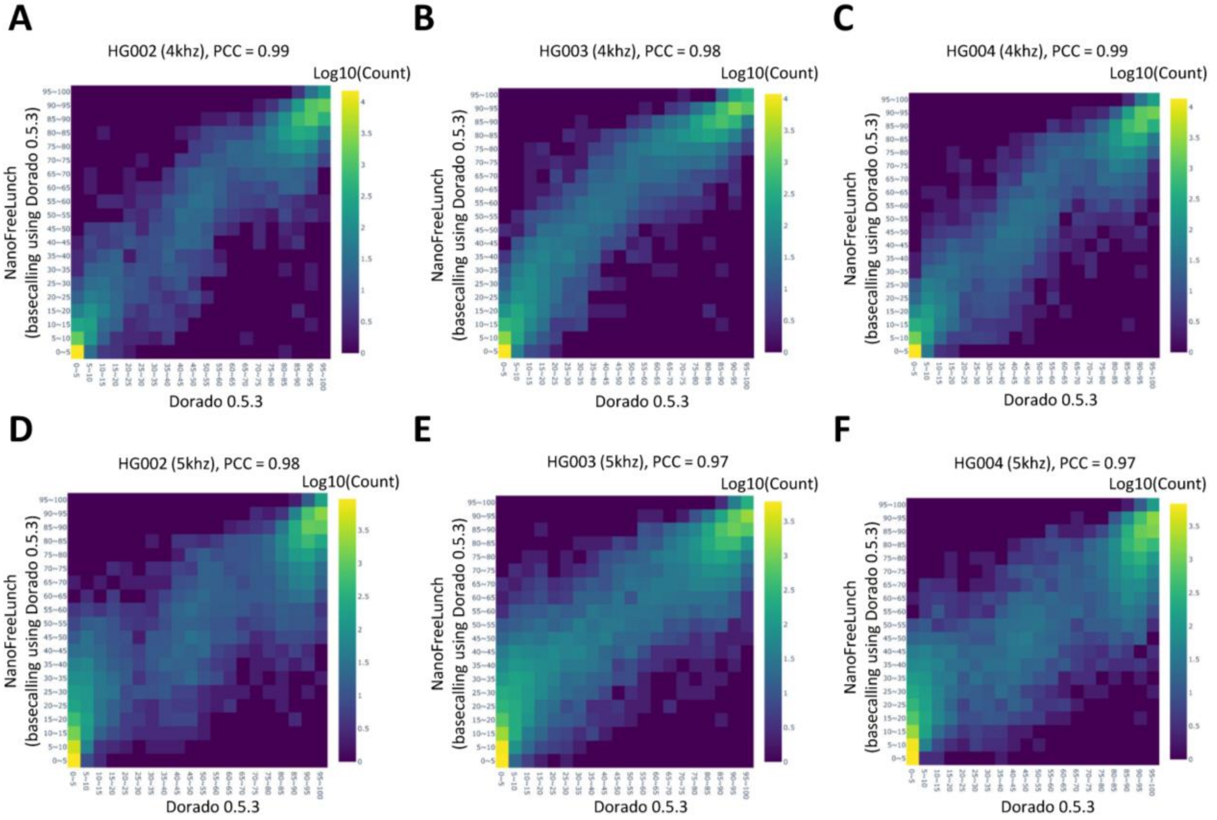

Supplementary Fig. S10. The region-level accuracy of NanoFreeLunch using Dorado 0.5.3 for basecalling on the Ashkenazim Trio data. The x-axis and y-axis are the average DNA methylation levels of each CpG island predicted by Guppy and NanoFreeLunch respectively. Predicted DNA methylation levels are segmented into 20 bins of equal size ranging from 0% to 100%. The color of each bin represents the base-10 logarithm transformation of the number of CpG islands within the bin. PCC denotes Pearson Correlation Coefficient. **A-C**, The results for the 4kHz data. **D-F**, The results for the 5kHz data.

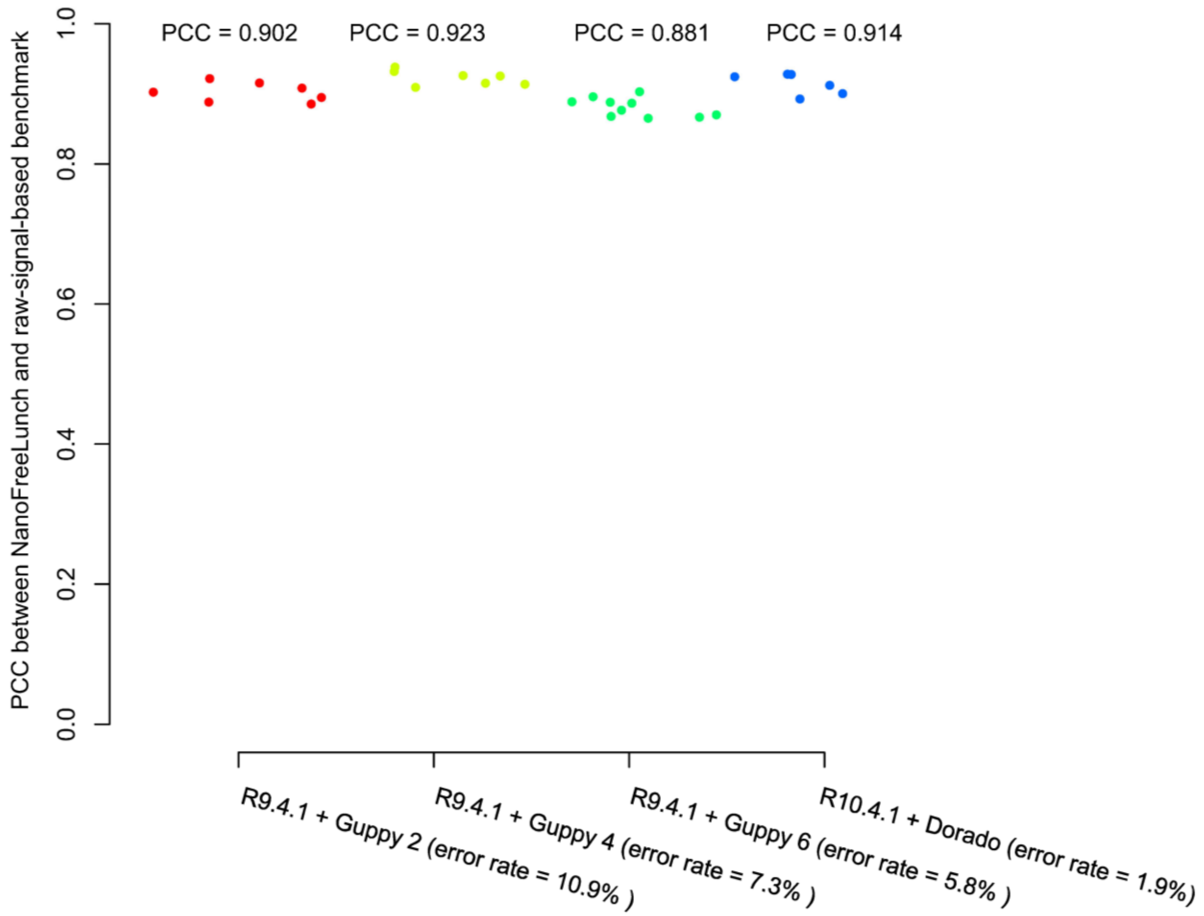

Supplementary Fig. S11. The accuracy of NanoFreeLunch using the data obtained by different flowcell types and basecallers. PCC represents Pearson Correlation Coefficient. The PCC in the figure is the average PCC of each category.

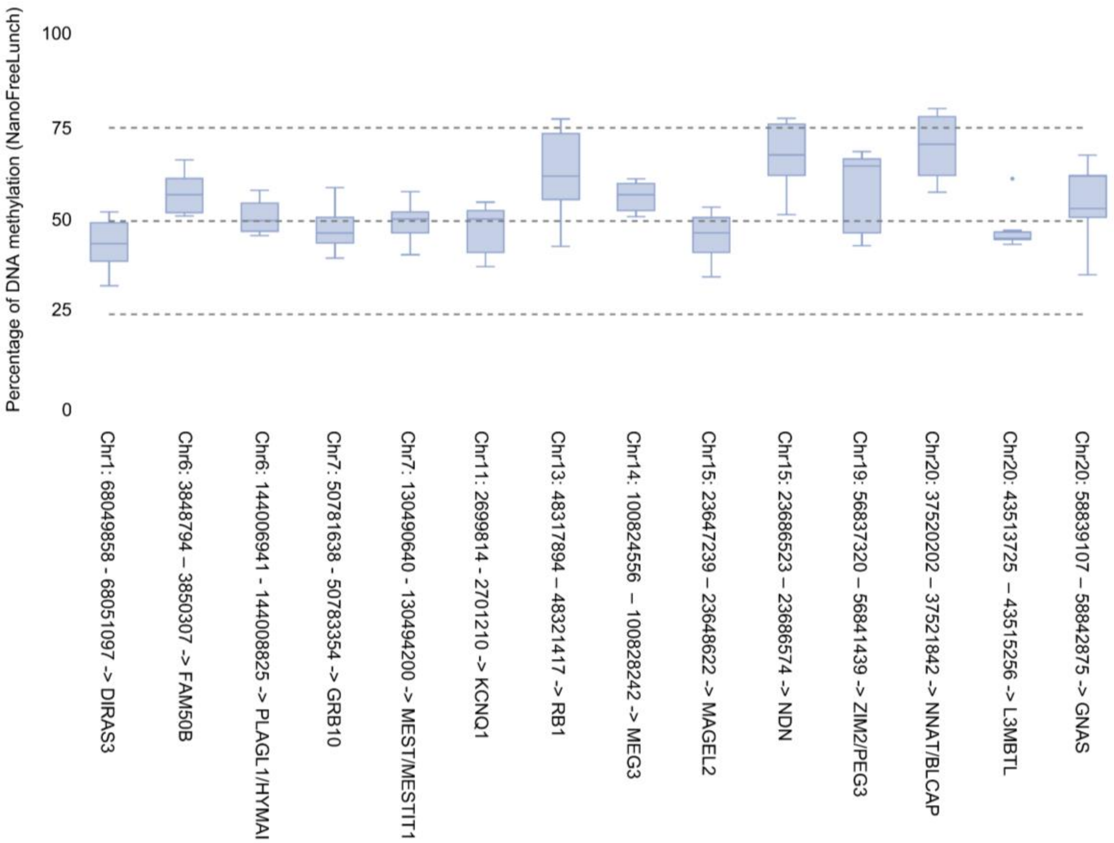

Supplementary Fig. S12. The average DNA methylation level of ICR predicted by NanoFreeLunch using Guppy 2.3.5 for basecalling. Each boxplot illustrates the distribution of the average DNA methylation levels of ICRs predicted by NanoFreeLunch using human pangenome data. The line in each box represents the median. The lower and upper bounds of the box correspond to the first (Q1) and third (Q3) quartiles, respectively. The lower fence is determined as the last sample point below 1.5 times the interquartile range (IQR), calculated as Q3 minus Q1. Similarly, the upper fence is identified as the last sample point above 1.5 times the IQR. In the x-axis, the region on the left of "->" is the genomic region of the ICR on GRCh38, and the gene symbol on the right is the putative gene associated with the ICR. The basecalling results of Guppy 2.3.5 are used as the input of NanoFreeLunch.

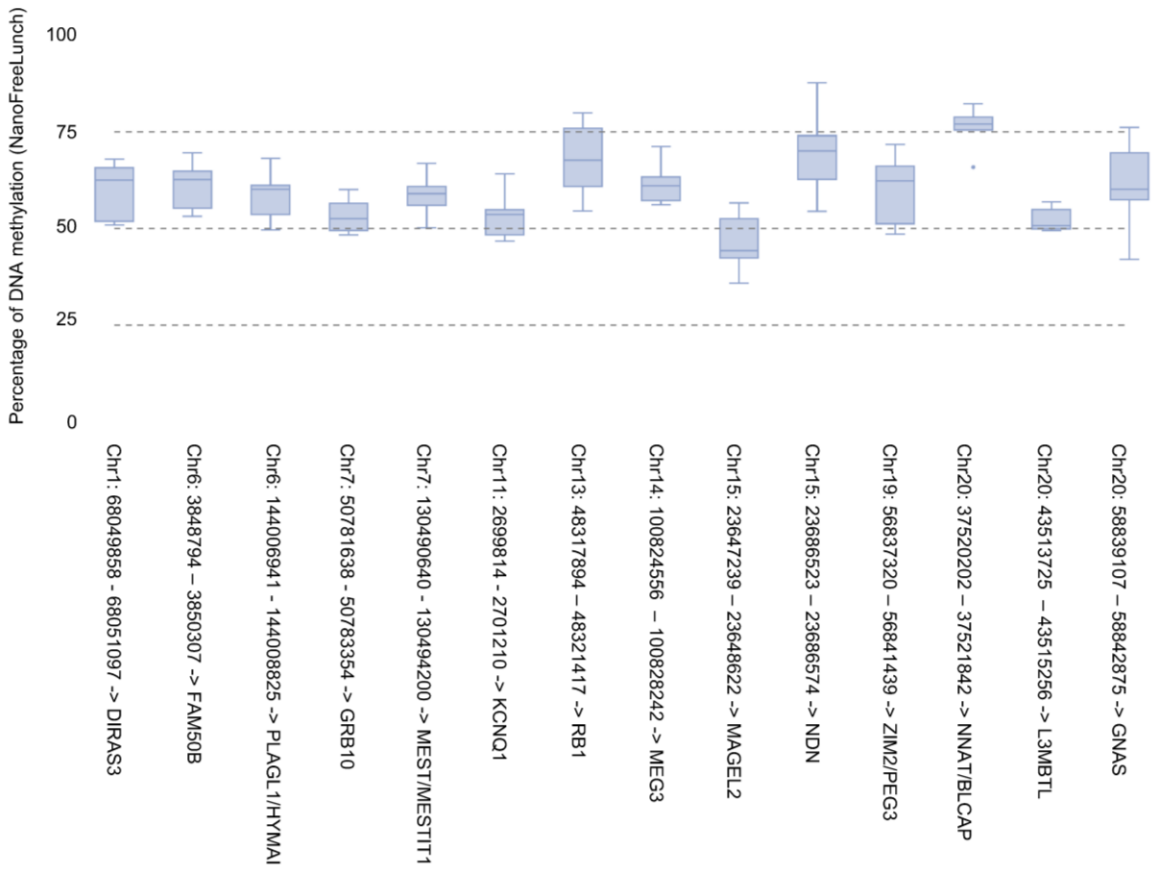

Supplementary Fig. S13. The average DNA methylation level of ICR predicted by NanoFreeLunch using Guppy 4.2.2 for basecalling. Each boxplot illustrates the distribution of the average DNA methylation levels of ICRs predicted by NanoFreeLunch using human pangenome data. The line in each box represents the median. The lower and upper bounds of the box correspond to the first (Q1) and third (Q3) quartiles, respectively. The lower fence is determined as the last sample point below 1.5 times the interquartile range (IQR), calculated as Q3 minus Q1. Similarly, the upper fence is identified as the last sample point above 1.5 times the IQR. In the x-axis, the region on the left of “->” is the genomic region of the ICR on GRCh38, and the gene symbol on the right is the putative gene associated with the ICR. The basecalling results of Guppy 4.2.2 are used as the input of NanoFreeLunch.

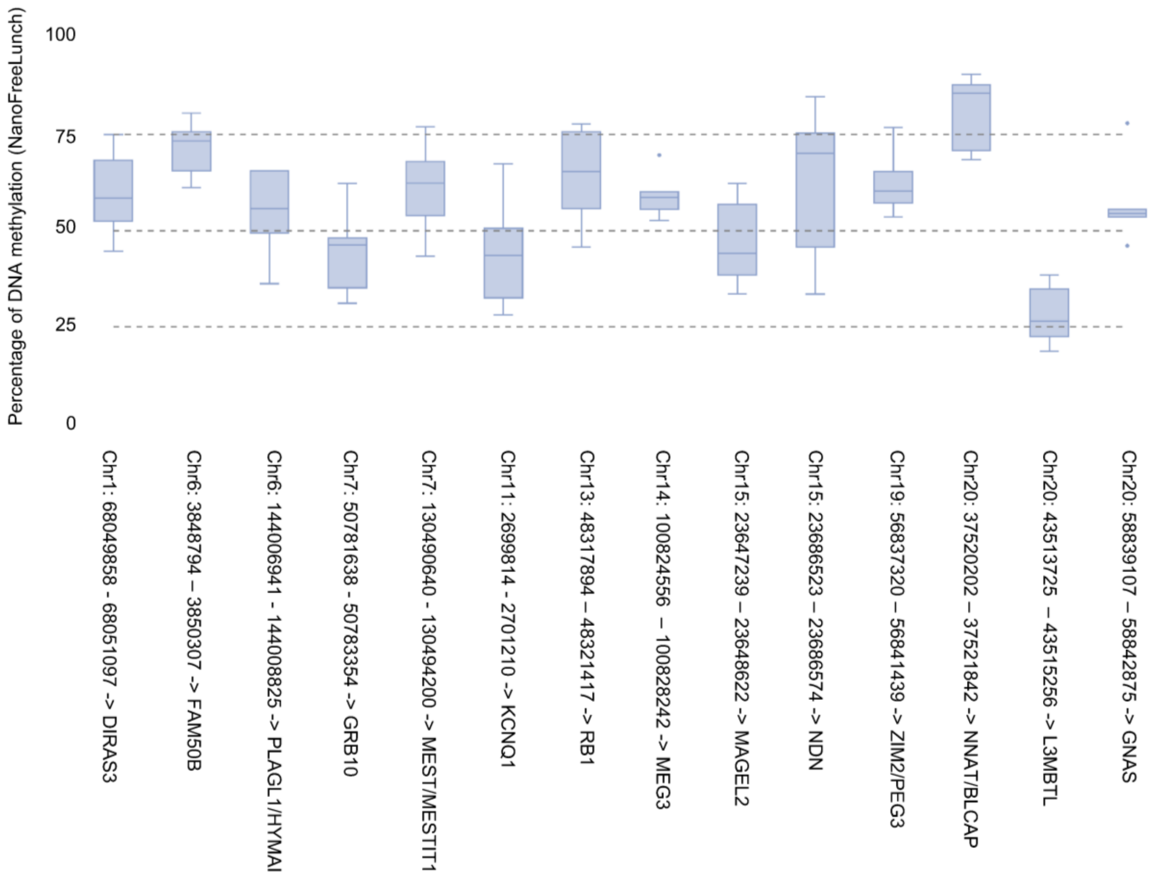

Supplementary Fig. S14. The average DNA methylation level of ICR predicted by NanoFreeLunch using Dorado 0.5.3 for basecalling. Each boxplot illustrates the distribution of the average DNA methylation levels of ICRs predicted by NanoFreeLunch using the R10 Ashkenazim trio data. The line in each box represents the median. The lower and upper bounds of the box correspond to the first (Q1) and third (Q3) quartiles, respectively. The lower fence is determined as the last sample point below 1.5 times the interquartile range (IQR), calculated as Q3 minus Q1. Similarly, the upper fence is identified as the last sample point above 1.5 times the IQR. In the x-axis, the region on the left of "->" is the genomic region of the ICR on GRCh38, and the gene symbol on the right is the putative gene associated with the ICR. The basecalling results of Dorado 0.5.3 are used as the input of NanoFreeLunch.

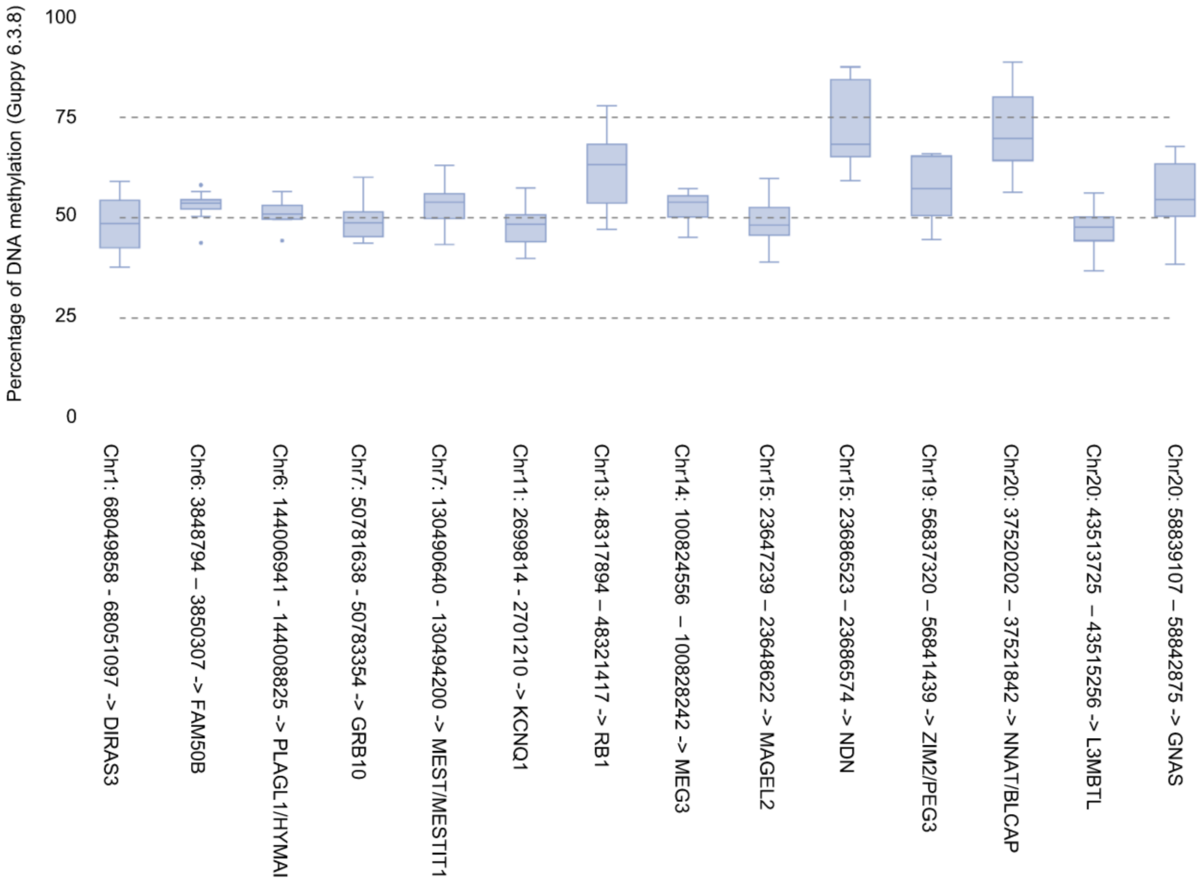

Supplementary Fig. S15. The average DNA methylation level of ICR predicted by Guppy 6.3.8. Each boxplot illustrates the distribution of the average DNA methylation levels of ICRs predicted by Guppy using human pangenome data. The line in each box represents the median. The lower and upper bounds of the box correspond to the first (Q1) and third (Q3) quartiles, respectively. The lower fence is determined as the last sample point below 1.5 times the interquartile range (IQR), calculated as Q3 minus Q1. Similarly, the upper fence is identified as the last sample point above 1.5 times the IQR. In the x-axis, the region on the left of “->” is the genomic region of the ICR on GRCh38, and the gene symbol on the right is the putative gene associated with the ICR.

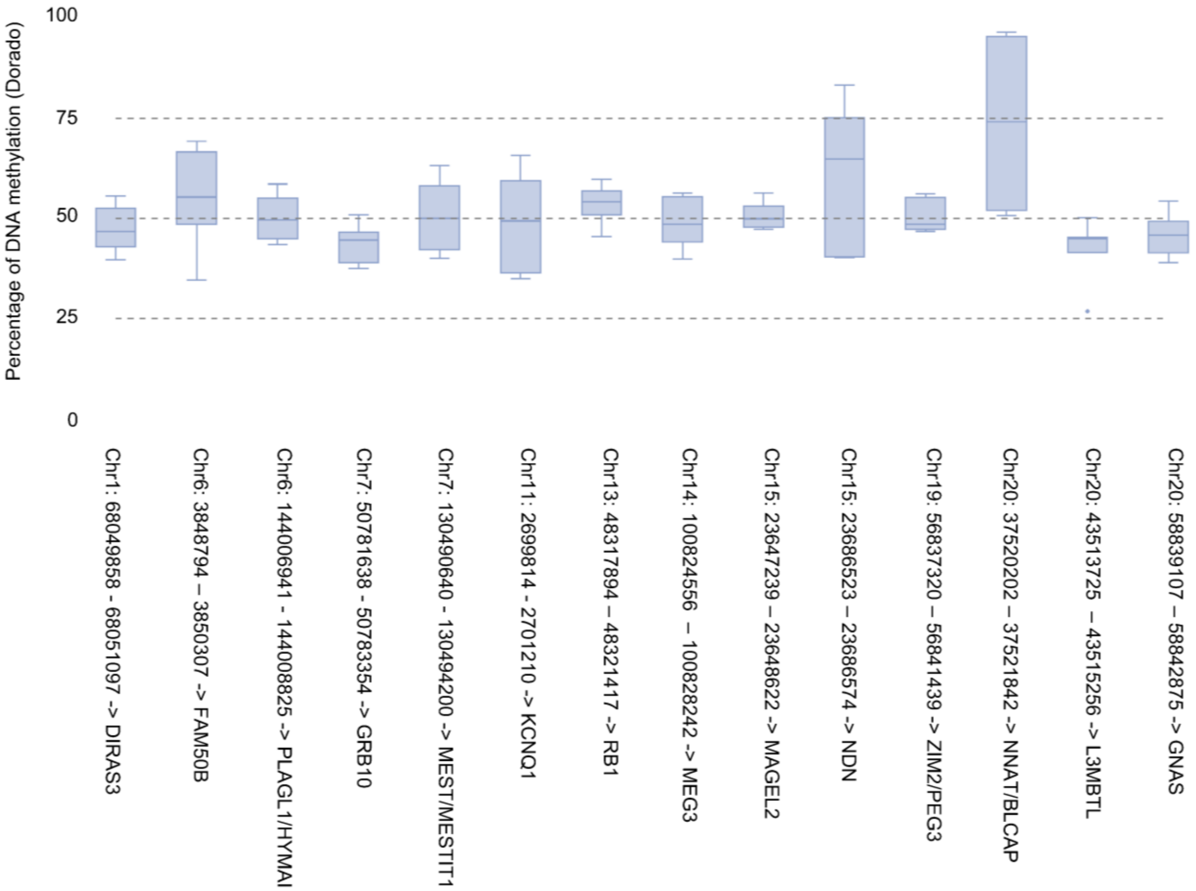

Supplementary Fig. S16. The average DNA methylation level of ICR predicted by Dorado 0.5.3. Each boxplot illustrates the distribution of the average DNA methylation levels of ICRs predicted by Dorado using the R10 Ashkenazim trio data. The line in each box represents the median. The lower and upper bounds of the box correspond to the first (Q1) and third (Q3) quartiles, respectively. The lower fence is determined as the last sample point below 1.5 times the interquartile range (IQR), calculated as Q3 minus Q1. Similarly, the upper fence is identified as the last sample point above 1.5 times the IQR. In the x-axis, the region on the left of "->" is the genomic region of the ICR on GRCh38, and the gene symbol on the right is the putative gene associated with the ICR.

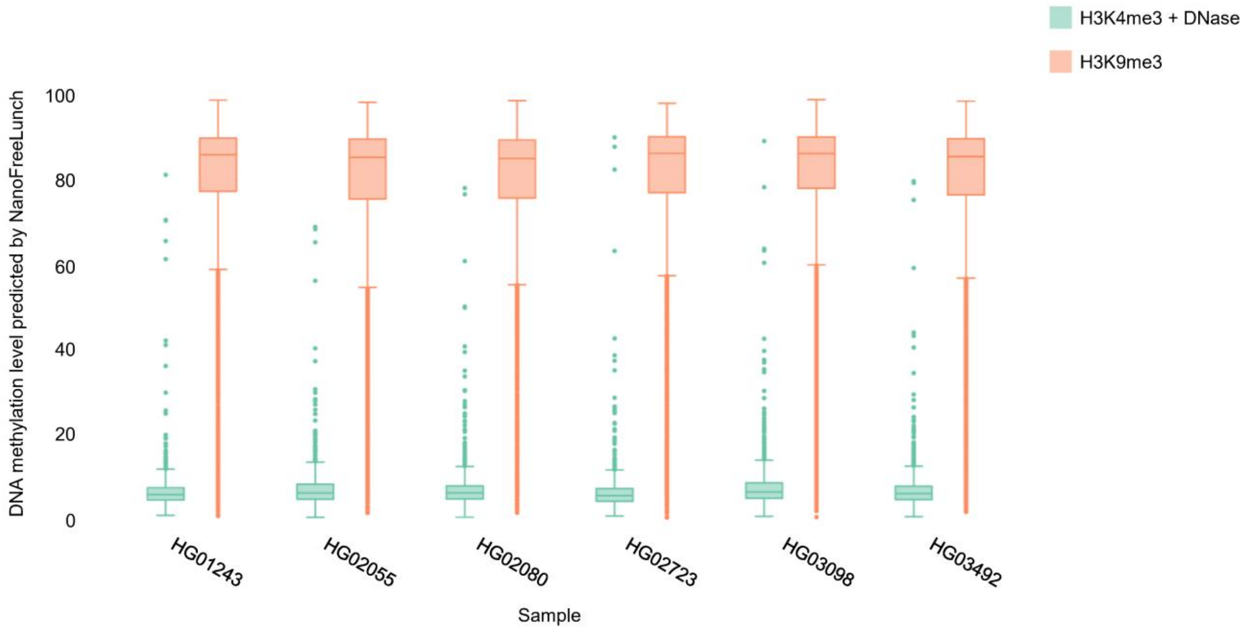

Supplementary Fig. S17. Comparing DNA methylation level predicted by NanoFreeLunch using Guppy 4.2.2 for basecalling with other epigenetic markers. Each boxplot depicts the distribution of average DNA methylation levels in H3K9me3 regions or DNase hypersensitive regions marked by H3K4me3, predicted by NanoFreeLunch using human pangenome data. Different colors represent distinct regions. The line in each box represents the median. The lower and upper bounds of the box correspond to the first (Q1) and third (Q3) quartiles, respectively. The lower fence is determined as the last sample point below 1.5 times the interquartile range (IQR), calculated as Q3 minus Q1. Similarly, the upper fence is identified as the last sample point above 1.5 times the IQR. The histone modification data and DNase data are obtained from the GM12878 cell line of ENCODE. The basecalling results of Guppy 4.2.2 are used as the input of NanoFreeLunch.

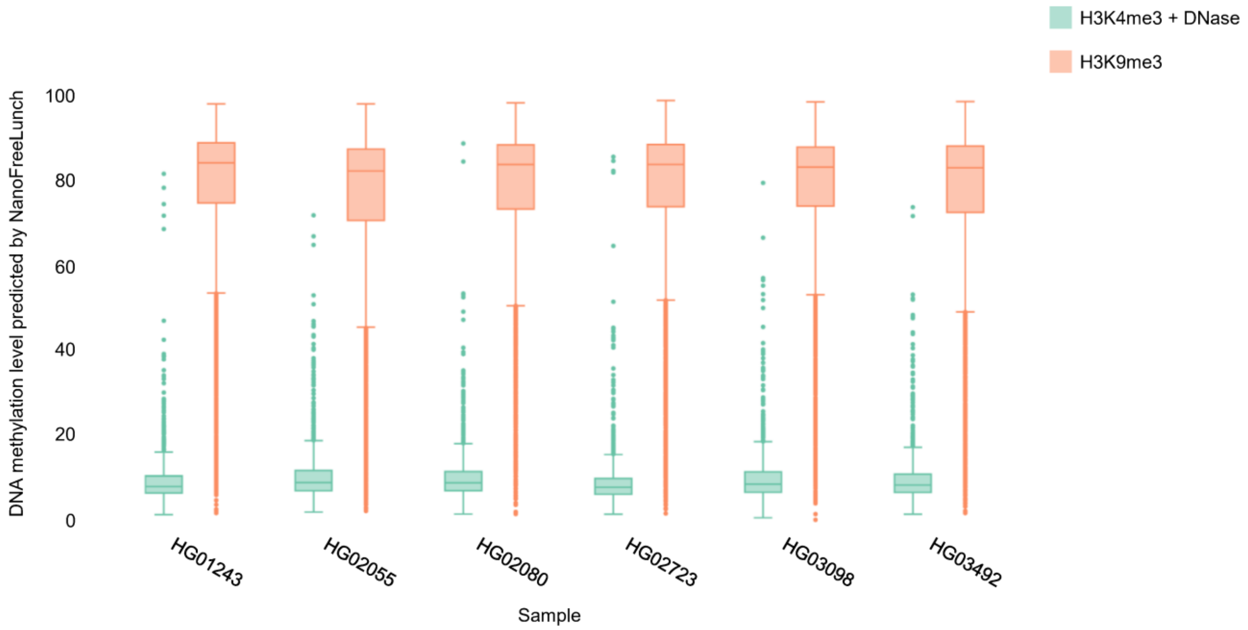

Supplementary Fig. S18. Comparing DNA methylation level predicted by NanoFreeLunch using Guppy 2.3.5 for basecalling with other epigenetic markers. Each boxplot depicts the distribution of average DNA methylation levels in H3K9me3 regions or DNase hypersensitive regions marked by H3K4me3, predicted by NanoFreeLunch using human pangenome data. Different colors represent distinct regions. The line in each box represents the median. The lower and upper bounds of the box correspond to the first (Q1) and third (Q3) quartiles, respectively. The lower fence is determined as the last sample point below 1.5 times the interquartile range (IQR), calculated as Q3 minus Q1. Similarly, the upper fence is identified as the last sample point above 1.5 times the IQR. The histone modification data and DNase data are obtained from the GM12878 cell line of ENCODE. The basecalling results of Guppy 2.3.5 are used as the input of NanoFreeLunch.

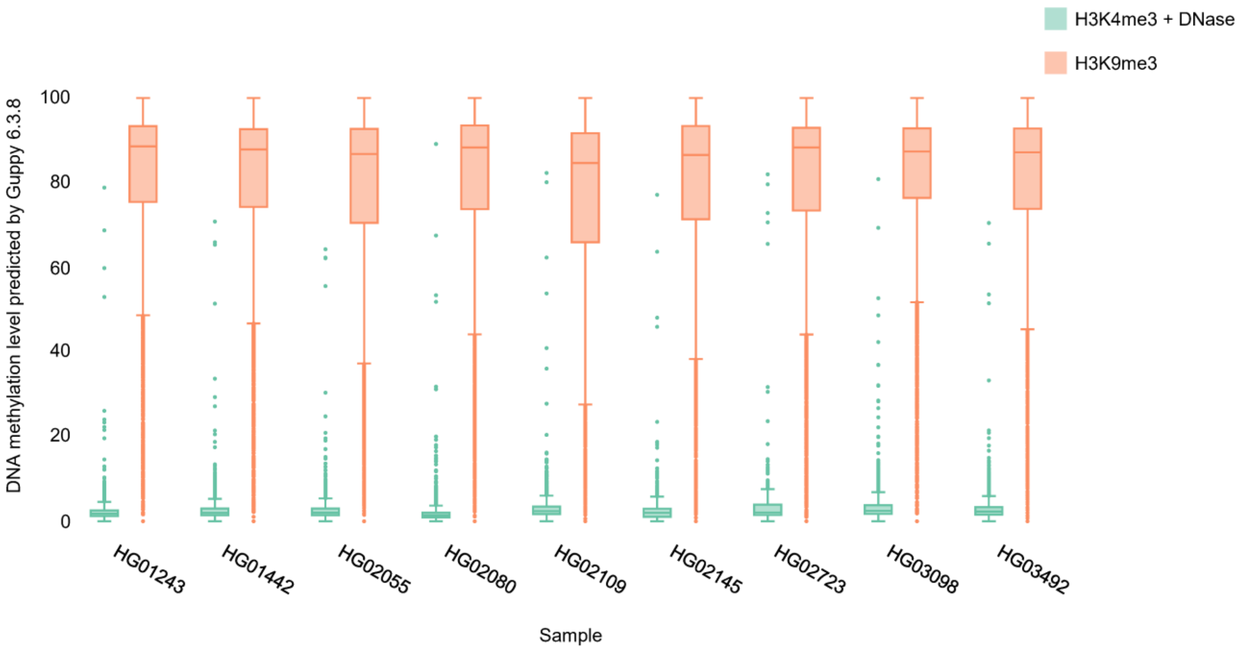

Supplementary Fig. S19. The average DNA methylation level of regions with different epigenetic markers predicted by Guppy 6.3.8. Each boxplot depicts the distribution of average DNA methylation levels in H3K9me3 regions or DNase hypersensitive regions marked by H3K4me3, predicted by Guppy using human pangenome data. Different colors represent distinct regions. The line in each box represents the median. The lower and upper bounds of the box correspond to the first (Q1) and third (Q3) quartiles, respectively. The lower fence is determined as the last sample point below 1.5 times the interquartile range (IQR), calculated as Q3 minus Q1. Similarly, the upper fence is identified as the last sample point above 1.5 times the IQR. The histone modification data and DNase data are obtained from the GM12878 cell line of ENCODE.

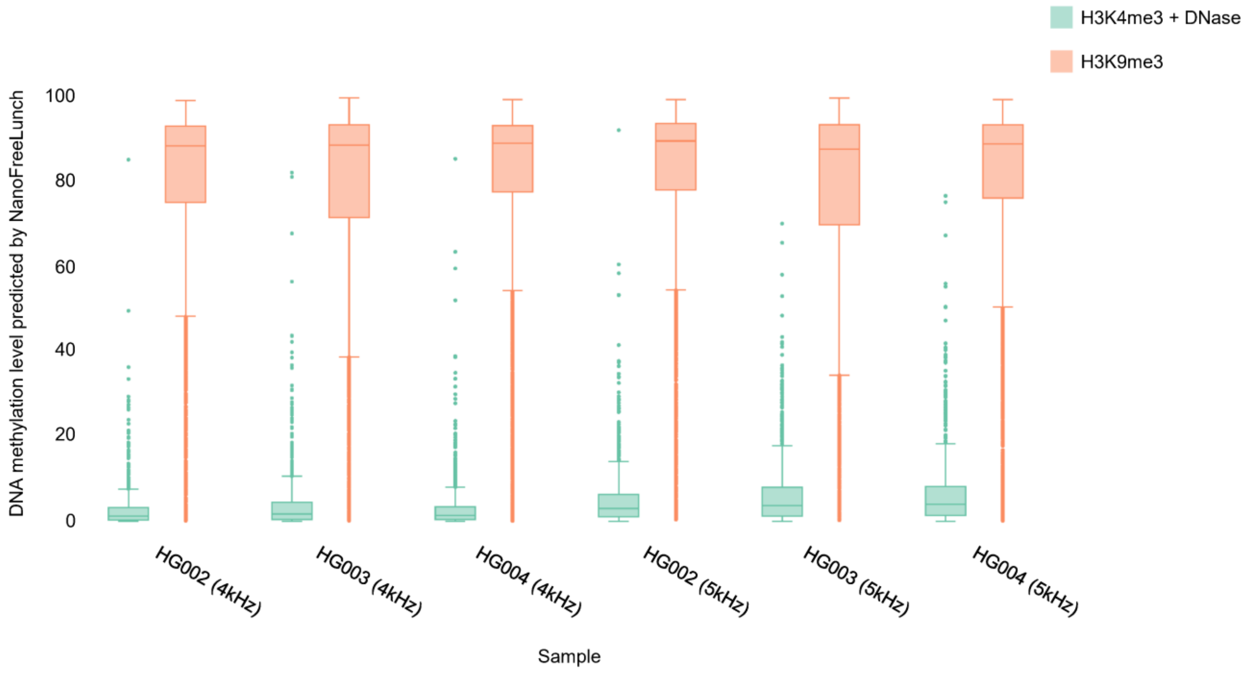

Supplementary Fig. S20. Comparing DNA methylation level predicted by NanoFreeLunch using Dorado 0.5.3 for basecalling with other epigenetic markers. Each boxplot depicts the distribution of average DNA methylation levels in H3K9me3 regions or DNase hypersensitive regions marked by H3K4me3, predicted by NanoFreeLunch using the R10 Ashkenazim trio data. Different colors represent distinct regions. The line in each box represents the median. The lower and upper bounds of the box correspond to the first (Q1) and third (Q3) quartiles, respectively. The lower fence is determined as the last sample point below 1.5 times the interquartile range (IQR), calculated as Q3 minus Q1. Similarly, the upper fence is identified as the last sample point above 1.5 times the IQR. The histone modification data and DNase data are obtained from the GM12878 cell line of ENCODE. The basecalling results of Dorado 0.5.3 are used as the input of NanoFreeLunch.

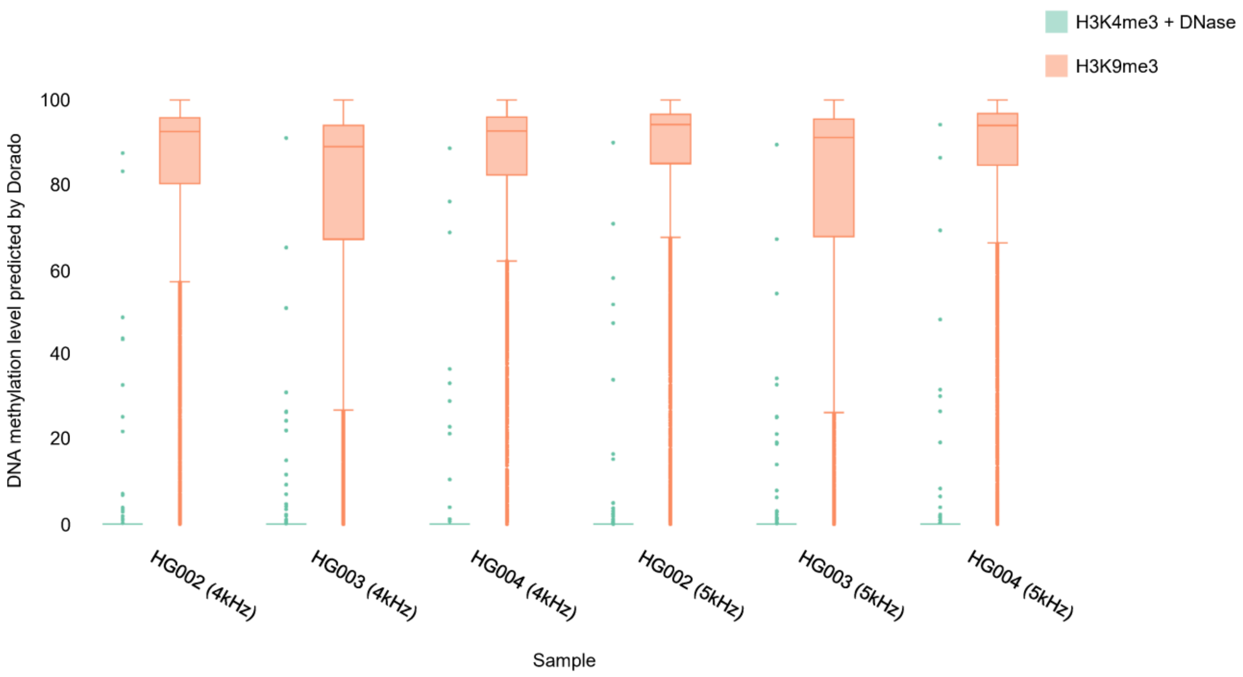

Supplementary Fig. S21. The average DNA methylation level of regions with different epigenetic markers predicted by Dorado 0.5.3. Each boxplot depicts the distribution of average DNA methylation levels in H3K9me3 regions or DNase hypersensitive regions marked by H3K4me3, predicted by Dorado using the R10 Ashkenazim trio data. Different colors represent distinct regions. The line in each box represents the median. The lower and upper bounds of the box correspond to the first (Q1) and third (Q3) quartiles, respectively. The lower fence is determined as the last sample point below 1.5 times the interquartile range (IQR), calculated as Q3 minus Q1. Similarly, the upper fence is identified as the last sample point above 1.5 times the IQR. The histone modification data and DNase data are obtained from the GM12878 cell line of ENCODE.

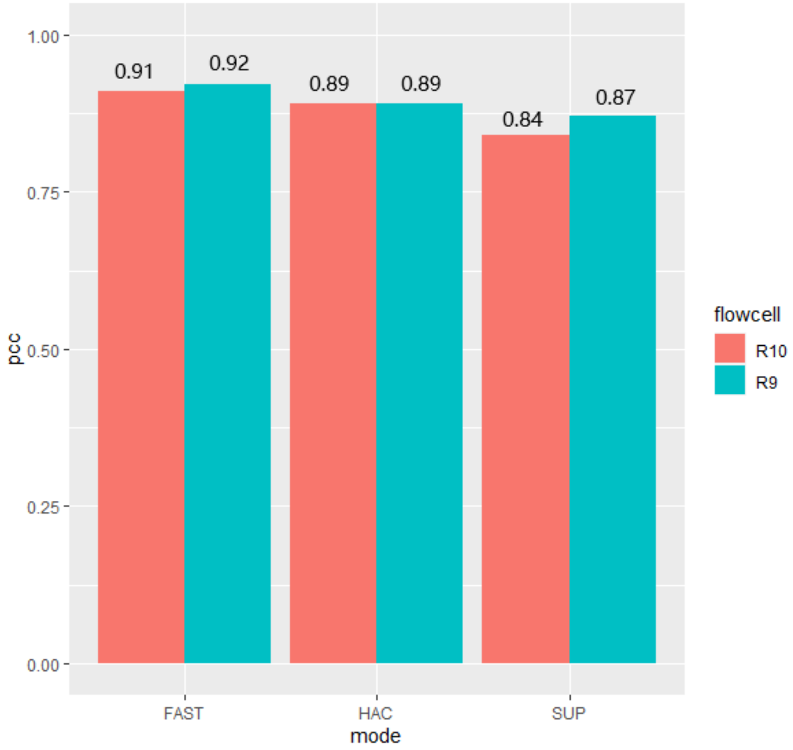

Supplementary Fig. 22. The impact of basecalling mode on the accuracy of NanoFreeLunch. Comparison of NanoFreeLunch performance using R9 (Guppy) and R10 (Dorado) data basecalled with FAST, HAC, and SUP modes. Bars show the Pearson correlation coefficient (PCC) between methylation levels predicted by NanoFreeLunch and those predicted by the respective basecaller.
