# Supplementary material for: Quantitative detection of DNA methylation from nanopore sequencing data without raw signals
Source: Gigascience. 2025 Oct 31;14:giaf113. doi: 10.1093/gigascience/giaf113 (PMC12576052; doi:10.1093/gigascience/giaf113)
Supplement: giaf113_GIGA-D-25-00019_Revision_1 [file giaf113_giga-d-25-00019_revision_1.pdf]

GigaScience

Quantitative Detection of DNA Methylation from Nanopore Sequencing Data without Raw Signals

--Manuscript Draft--

**Manuscript Number:**	GIGA-D-25-00019R1
**Full Title:**	Quantitative Detection of DNA Methylation from Nanopore Sequencing Data without Raw Signals
**Article Type:**	Technical Note
**Funding Information:**	National Natural Science Foundation of China (32370695) — Assistant Professor Zhixing Feng<br>National Natural Science Foundation of China (32470684) — Associate Professor Huijuan Feng<br>Key Technologies Research and Development Program (2022YFC2703400) — Assistant Professor Zhixing Feng, Associate Professor Huijuan Feng
**Abstract:**	Nanopore sequencing has revolutionized the field of epigenomics by enabling direct detection of DNA methylation without sample preprocessing. It is theoretically possible to reutilize any nanopore sequencing data to construct epigenomes. However, reutilizing the data in practice is challenging with existing methods because they rely on raw signals from nanopore sequencing, which are absent in more than 98% of public nanopore sequencing data. Moreover, storing raw signals for large-scale sequencing projects is impractical due to their enormous file sizes. To overcome these limitations, we propose a novel method, NanoFreeLunch, which can quantitatively detect DNA methylation without the need for raw signals by modeling base quality values and sequencing error patterns. Our results demonstrated a strong correlation between the DNA methylation levels estimated by NanoFreeLunch and those estimated by the benchmark methods, ranging from 0.87 to 0.94 for individual genomic loci and from 0.97 to 0.99 for average methylation levels of genomic regions. With the rapid accumulation of nanopore sequencing data, the development of NanoFreeLunch will enable the construction of epigenomes on an unprecedented scale, facilitating novel insights into the role of DNA methylation in health and disease.
**Corresponding Author:**	Zhixing Feng<br>Xinhua Hospital Affiliated to Shanghai Jiaotong University School of Medicine: Shanghai Jiaotong University School of Medicine Xinhua Hospital<br>Shanghai, CHINA
**Corresponding Author Secondary Information:**	
**Corresponding Author's Institution:**	Xinhua Hospital Affiliated to Shanghai Jiaotong University School of Medicine: Shanghai Jiaotong University School of Medicine Xinhua Hospital
**Corresponding Author's Secondary Institution:**	
**First Author:**	Zhixing Feng
**First Author Secondary Information:**	
**Order of Authors:**	Zhixing Feng<br>Chenxi Zhang<br>Shuo Jin<br>Jiale Niu<br>Huijuan Feng
**Order of Authors Secondary Information:**	

Powered by Editorial Manager® and ProduXion Manager® from Aries Systems Corporation

Response to Reviewers:	(The complete response to the reviewers is provided in the file 'response-to-reviewers.docx', which may be found at the end of the system-generated PDF of the revised manuscript. Please note that the response included here is text-only and incomplete, as figures could not be uploaded through the submission system.)<br><br>We sincerely thank the reviewers for their constructive feedback. The comments are very helpful in improving the quality of this manuscript. Below, we provide a detailed point-by-point response to the reviewers' comments, and we highlight the changes in the revised manuscript. The page and line numbers cited in the responses below refer to the revised manuscript.<br><br>Reviewer reports:<br>Reviewer #1: In the manuscript "Quantitative Detection of DNA Methylation from Nanopore Sequencing Data without Raw Signals", the authors present a novel method to infer DNA methylation from sequence data without the need for the original signal data. This work describes an exciting new tool to utilize existing ONT data for epigenomics without the need for the massive POD5/FAST5 files.<br><br>We sincerely thank the reviewer for highlighting the significance of our study. We also appreciate the constructive comments and have addressed them point-by-point below.<br>## Major comments<br><br>One of the major claims of this work is that it can remove the need to store POD5/FAST5 files which is expensive due to their size. However, they do not offer any indication of resource requirements for model training or training duration. This is particularly relevant to researchers who would like to leverage this tool but work on species other than human as they would likely need to train their own species specific model. Moreover, for other species, there is often far less data available to train such models. Can the authors offer a recommendation of the minimum training data requirements?<br><br>We agree with the reviewer that sample size for model training is important. To address this issue, we performed saturation analysis by downsampling the human training data (chr10 of HG01109) to 1%, 10%, 20%, 50%, 75%, and 90% respectively. The results (Fig. R1) show that accuracy plateaus at approximately 883,912 CpG sites (highlighted in red). While increasing sample size beyond this point yields no further gains, training with 117,855 CpG sites achieves an accuracy of 0.85 (PCC between NanoFreeLunch and Guppy), which represents 96% of the peak accuracy (PCC = 0.89) achieved with the full dataset (tested on chr6 of HG01243). This suggests that using roughly 118,000 CpG sites offers a favorable balance between data requirements and performance for the human data analyzed. While it is difficult to define an exact universal minimum training data requirement applicable to all species, these results provide valuable insights into the relationship between training sample size and accuracy. Researchers working on other species can adapt this saturation analysis to their own datasets to estimate suitable training sizes given their specific performance goals and data availability.<br><br>Line 289: The authors used the HAC option for basecalling. Did the authors test with different base calling models, I.e., SUP or FAST? Can the authors offer some discussion as to why they picked this model and whether or not they tested the other models? If they tested the other models was there a difference in the correlations?<br><br>Yes, we tested basecalling with FAST, HAC, and SUP modes. We found that the choice of basecalling mode has a minor, but measurable, impact on NanoFreeLunch's accuracy. The results show that FAST mode yielding slightly higher accuracy than HAC mode, and SUP mode yielding slightly lower accuracy for both R9 and R10 data (Fig. R2). In the previous version of the manuscript, we used HAC mode because it is commonly used and the default recommended mode in MinKNOW, the official sequencer software that wraps Guppy/Dorado for basecalling (https://nanoporetech.com/document/experiment-companion-minknow#starting-a-sequencing-run-on-promethion-p2i).<br><br>The exact reason for this discrepancy between basecalling modes is non-trivial to determine. Our results suggest that the relationship between basecalling accuracy and

NanoFreeLunch accuracy is complex and non-monotonic. For instance, while R10 data has significantly higher basecalling accuracy than R9 data, NanoFreeLunch accuracy is similar for both flowcell types (Fig. R2). This indicates that the specific types of error/QV pattern introduced by different basecalling modes (FAST, HAC, SUP) may have complicated and hard-to-interpret effects on the underlying features used by NanoFreeLunch to predict methylation.

We added a paragraph in Discussion (page 22, line 241-255) as following and added Fig.R2 as Supplementary Fig. S22 to revised manuscript.
“In this study, we used HAC mode for basecalling, which is commonly used and recommended mode since it has a good balance between speed and accuracy. However, basecalling mode might affect methylation detection of NanoFreeLunch since different modes might have different error and QV patterns. Using models trained on chromosome 10 (HG01109 for R9, HG002 for R10) and tested on chromosome 6 (HG01243 for R9, HG003 for R10), we found that FAST mode yielded slightly higher accuracy than HAC mode, while SUP mode yielded slightly lower accuracy for both R9 and R10 data (Supplementary Fig. S22). This observed dependency, coupled with the complex and non-monotonic relationship between basecalling accuracy and NanoFreeLunch accuracy, indicates that the specific error and QV characteristics introduced by different basecalling modes may have complex and hard-to-interpret effects on the features used by NanoFreeLunch for methylation prediction.”

Minor comments

Line 12. I'm not sure it's completely correct to say it requires no sample preprocessing, as there is still the necessary library prep for Nanopore sequencing.

We thank the reviewer to pointing this out. To avoid confusion, we have changed it to “Nanopore sequencing has revolutionized the field of epigenomics by enabling direct detection of DNA methylation without additional sample preprocessing such as bisulfite treatment” in the revised manuscript.

Line 54: Consider changing to "…impact on the raw electrical signals detected by the sequencer."

We thank the reviewer for this comment. This is more accurate. We have changed manuscript according to the reviewer’s suggestion.

Line 62: Change "modification" to "methylation".

We have changed the wording in the revised manuscript.

Line 281: I believe it should be written as "… files of the R10.4.1 flowcell (abbreviated as R10)…"

We have changed manuscript according to the reviewer’s suggestion. The revised part is now at line 293 of the revised manuscript.

Reviewer #2: This is an interesting and useful approach to calling DNA methylation from ONT data when only the sequence data are available. As the author's comment, the vast majority of ONT data present in the public archives do not have the raw data available, and few of the sequence only datasets have methylation calls included (for example as tags in the BAMs). For this reason, a method to accurately call methylation using just the base calls and quality values would be very valuable.
I found the article to be well written and the example datasets were well chosen, showing the performance of their method with datasets called using various different sequencing and analysis pipelines. This gives confidence that their approach will be usable for many of the available ONT datasets.

We thank the reviewer for the insightful comments and for emphasizing the importance of our work. Below, we provide a point-by-point response to the comments.

I have just a few general comments:
(1) The authors discuss the performance of their method on cytosines in CpG context

only (as far as I could tell). Have the authors tested their approach with other contexts? This would require testing on datasets from tissues with high-levels of non-CpG methylation such as stem cells or neural tissue.

Yes, our current study focuses exclusively on CpG methylation. While non-CpG methylation detection is a fascinating area for future work, we limited our scope to CpG contexts for two key reasons:
1.ONT-based CpG detection is well validated: Raw-signal-based methylation detection has been rigorously validated only for CpG contexts in independent studies (e.g., SEQC2, large-scale nanopore sequencing in Icelandic populations).
2.Lack of Non-CpG Standards: Though Dorado’s latest update offers non-CpG detection, no third-party validation exists for non-CpG accuracy.
To support future extensions, NanoFreeLunch’s training CLI allows retraining for non-CpG contexts once the field matures. We appreciate this suggestion and will explore it in follow-up work.

(2) Have the authors tested the approach on non-human (or invertebrate/plant samples? It would be interesting to know how general the approach is.

We agree with the reviewer that it is interesting to test if NanoFreeLunch can be applied to other species. We tested the pre-trained human model used in the study (trained on human data) on zebrafish (Danio rerio, s3://genomeark/species/Danio_rerio/fDanRer18/genomic_data/ont/pod5/) and rice (Oryza sativa, SRA ID is SRR16080273) data. The results show that despite of minor accuracy loss compared to the results on human data, the pre-trained model achieved a good accuracy on the zebrafish data (PCC = 0.87). However, we observed a substantial accuracy drop in the rice data (PCC = 0.65). We hypothesize this discrepancy occurs because rice exhibits substantial non-CpG methylation, whereas CpG methylation dominates in zebrafish and humans (i.e., the same primary methylation motif). Non-CpG methylation may affect local and global QV patterns and error profiles, thereby affecting CpG methylation detection. Since the model trained on human data explicitly accounts for sequence context effects and performs well on the zebrafish data, it is unlikely that the reduced accuracy on the rice data is due to genomic sequence differences between humans and rice.

To test this hypothesis, we trained a model on Arabidopsis thaliana data (SRA ID: SRR16149191) and predicted CpG methylation in rice. These species have highly divergent genome sequences but both exhibit substantial non-CpG methylation (CHG and CHH). Results demonstrate NanoFreeLunch achieves high accuracy (PCC = 0.91) for rice CpG methylation prediction using the Arabidopsis-trained model. This indicates that for species with CpG-dominated methylation contexts, the pre-trained model is directly applicable. For species with substantial non-CpG methylation, retraining on species with identical methylation motifs is required.

(3) It would be useful to have some indication of the computational requirements. What would be the requirements (cpu, time, memory etc.) to call a 30x human genome for example?

We tested the model on a server equipped with 2× Intel Xeon Scalable Cascade Lake 6248 (2.5GHz, 20 cores, 40 threads) CPUs and 12× Samsung 16GB DDR4 ECC REG 2666 (totaling 192GB RAM). The computational time for a single human genome is approximately 16 hours using the default chunk size of NanoFreeLunch. The chunk size impacts memory usage: larger chunks require more memory but improve runtime by reducing file I/O overhead. With the default setting, memory usage remains under 32GB, which is within the capabilities of standard workstations or HPC clusters.

(4) As far as I can tell each cytosine is called independently of its neighbours. If this is correct, and given that methylation values are highly correlated between neighboring sites, have the authors considered using information from nearby cytosines?

We agree with the reviewer that neighboring CpG sites may offer valuable information for detecting DNA methylation. We explored this idea by incorporating the maximum error/QV correlation with neighboring CpG sites as an additional feature in an earlier version of NanoFreeLunch. However, we observed no improvement in accuracy. A

	possible explanation is that the current model already implicitly captures the correlation between neighboring CpG sites, as it includes joint error rates and QV correlations across all adjacent sites (including CpG sites, but without giving them special treatment).
Additional Information:	
Question	Response
Are you submitting this manuscript to a special series or article collection?	No
**Experimental design and statistics**<br><br>Full details of the experimental design and statistical methods used should be given in the Methods section, as detailed in our Minimum Standards Reporting Checklist. Information essential to interpreting the data presented should be made available in the figure legends.<br><br>Have you included all the information requested in your manuscript?	Yes
**Resources**<br><br>A description of all resources used, including antibodies, cell lines, animals and software tools, with enough information to allow them to be uniquely identified, should be included in the Methods section. Authors are strongly encouraged to cite Research Resource Identifiers (RRIDs) for antibodies, model organisms and tools, where possible.<br><br>Have you included the information requested as detailed in our Minimum Standards Reporting Checklist?	Yes
**Availability of data and materials**<br><br>All datasets and code on which the conclusions of the paper rely must be either included in your submission or deposited in publicly available repositories (where available and ethically appropriate), referencing such data using	Yes

a unique identifier in the references and in the "Availability of Data and Materials" section of your manuscript.<br><br>Have you have met the above requirement as detailed in our [Minimum Standards Reporting Checklist]?	
GigaScience has policies and guidelines in place for the use of generative AI-writing tools such as ChatGPT. If you have used such writing tools to assist with writing the manuscript this must be declared and cited in the text. Authors should not list AI-writing tools and other AI-assisted technologies as an author or co-author and should acknowledge that they are fully responsible for text generated or refined by AI-writing tools.<p><br><br>A summary of use (particularly in the introduction or among methods) needs to be included at the end of the paper, and the outputs should also be included as a supplementary file hosted in GigaDB or other open repositories. Please read our guidelines for more information. <p><br><br>By submitting to GigaScience, you are aware of the journal's AI-writing tools policy, and if you have declared use of such tools below, you have acknowledged this where appropriate in your manuscript and have made a summary of use and outputs available. <p><br><b>AI-assisted writing tools have been used in the preparation of this manuscript?	No

Manuscript Click here to access/download;Manuscript;Manuscript_final.docx

Quantitative Detection of DNA Methylation from Nanopore Sequencing Data without Raw Signals

Zhixing Feng[1,*], Chenxi Zhang[2], Shuo Jin[2], Jiale Niu[2], and Huijuan Feng[2,*]

[1] Department of Clinical Genetics, Xinhua Hospital affiliated to Shanghai Jiao Tong University School of Medicine, Shanghai, 200092, China

[2] Department of Computational Biology, School of Life Sciences, Fudan University, Shanghai, 200438, China

* To whom correspondence should be addressed. Email: fengzhixing@shsmu.edu.cn.

Correspondence may also be addressed to huijuanfeng@fudan.edu.cn.

ORCID iDs: Zhixing Feng [0000-0003-0308-8549]; Huijuan Feng [0000-0002-4005-560X];

Abstract

Nanopore sequencing has revolutionized the field of epigenomics by enabling direct detection of DNA methylation without additional sample preprocessing such as bisulfite treatment. It is theoretically possible to reutilize any nanopore sequencing data to construct epigenomes. However, reutilizing the data in practice is challenging with existing methods because they rely on raw signals from nanopore sequencing, which are absent in more than 98% of public nanopore sequencing data. Moreover, storing raw signals for large-scale sequencing projects is impractical due to their enormous file sizes. To overcome these limitations, we propose a novel method, NanoFreeLunch, which can quantitatively detect DNA methylation without the need for raw signals by modeling base quality values and sequencing error patterns. Our results demonstrated

a strong correlation between the DNA methylation levels estimated by NanoFreeLunch and those estimated by the benchmark methods, ranging from 0.87 to 0.94 for individual genomic loci and from 0.97 to 0.99 for average methylation levels of genomic regions. With the rapid accumulation of nanopore sequencing data, the development of NanoFreeLunch will enable the construction of epigenomes on an unprecedented scale, facilitating novel insights into the role of DNA methylation in health and disease.

Keywords

Nanopore sequencing, DNA methylation, computational methods, machine learning.

Introduction

DNA methylation plays important roles in many biological processes, such as regulating gene expression, maintaining genome stability, gene imprinting, and X chromosome inactivation [1–3]. It is also an important biomarker for diseases, including congenital disorders and cancer [4–6]. Each genomic locus can be methylated, unmethylated, or partially methylated. Although DNA methylation is a dynamic marker affected by both genetics and environment [7,8], the methylation status of many genomic regions is precisely regulated and can cause a wide range of diseases if disturbed. For example, the 15q11-q13 region regulates the imprinting of multiple genes and is approximately 50% methylated, with only one of the two haplotypes fully methylated in healthy individuals. Full methylation of this region causes Angelman syndrome, while full unmethylation causes Prader–Willi syndrome [1]. Therefore, precise quantification of DNA methylation is critical for understanding the function of DNA methylation and determining its relationship with phenotypes and diseases.

Currently, the most widely used approaches for detecting DNA methylation quantitatively are based on next-generation sequencing (NGS) or microarrays. The sample is treated with bisulfite to mutate unmethylated cytosine to thymine while keeping 5-methylcytosine (5mC) and 5-hydroxymethylcytosine (5hmC) unchanged, and the DNA methylation level for each genomic locus can be quantified by the proportion of unmutated cytosine in NGS or the relative signal intensity in microarrays [9–11]. Although DNA methylation can be quantified with these methods, a major limitation is that they require the sample to be treated with bisulfite before sequencing or probe hybridization. Therefore, the vast majority of the existing NGS or microarray data cannot be reutilized to detect DNA methylation because many of them were designed for genotyping and not treated with bisulfite.

Nanopore sequencing provides a revolutionary platform for generating genomes and epigenomes simultaneously since it can detect DNA methylation directly without sample preprocessing, such as bisulfite treatment or immunoprecipitation. DNA methylation is retained during the sequencing process and has an impact on the raw electrical signals detected by the sequencer [12]. A machine learning model can be built to predict DNA methylation by extracting features from the raw signals of nanopore sequencing [12]. Therefore, it is theoretically possible to reutilize any nanopore sequencing data to study DNA methylation even if the data are generated for other purposes, such as studying structural variation. However, achieving this goal is challenging in practice due to limitations in existing methods [12–19]. The existing tools including Oxford Nanopore's official software (e.g., Guppy and Dorado) and third-party solutions (e.g., Nanopolish, DeepMod, and DeepSignal) rely on raw signal data stored in FAST5/POD5 files for DNA methylation detection [12–19]. These raw signal files are very large, often exceeding one

terabyte for a single ~20x human genome dataset, making them expensive to store, difficult to transfer, and rarely shared in public repositories. For example, in the SRA database (Sequence Read Archive) [20], there are 742,566 records of Oxford Nanopore genome sequencing (ONT) data as the study was conducted, but only 1.5% of the data include raw-signal files and the percentage has decreased over the years (**Fig. 1**). This makes it impossible to harness large-scale DNA methylation information from most datasets with the existing methods. As large-scale nanopore sequencing data accumulate rapidly [21], it is also unsustainable to store all the raw-signal files of nanopore sequencing because the size of 20,000 20x human genome datasets is approximately 20 petabytes, which is almost the total size of the SRA database [22]. More than 4,000 nanopore sequencing datasets of human genomes have been published in the last three years [21,23], and the accumulation of such data has accelerated remarkably in recent years (**Fig. 1**). Therefore, alternative approaches for detecting DNA methylation without reliance on raw-signal files are urgently needed.

To address the challenge of reutilizing nanopore sequencing data to construct epigenomes, we introduce NanoFreeLunch, a computational framework for quantitatively detecting DNA methylation from basecalled FASTQ files via a novel approach to model base quality value (QV) and sequencing error patterns. NanoFreeLunch has undergone extensive testing on three independent datasets of 16 nanopore sequencing experiments. The DNA methylation levels predicted by NanoFreeLunch are highly consistent with those predicted by benchmarking methods, including raw-signal-based algorithms and conventional bisulfite sequencing. The correlation ranged from 0.87 to 0.94 for the DNA methylation level of each CpG site and from 0.97 to 0.99 for the average methylation level of the CpG islands. The results of NanoFreeLunch are

also consistent with established epigenetic knowledge. The partial methylation of imprinting control regions (ICRs), hypomethylation of regions with H3k4me3 histone modification, and hypermethylation of regions with H3k9me3 histone modification can be reliably detected by NanoFreeLunch. As nanopore sequencing data accumulate rapidly, NanoFreeLunch represents a powerful tool enabling the construction of epigenomes on an unprecedented scale by reutilizing the existing data and establishing the relationships among DNA methylation, genotypes, and phenotypes.

Results

Detecting DNA methylation by modeling sequencing error patterns and base quality values

NanoFreeLunch leverages the impact of DNA methylation on base QVs and error patterns to quantitatively detect DNA methylation. As we have previously reported, DNA methylation has an impact on the error patterns of nanopore sequencing data [23]. For each genomic locus of interest, we obtained the aligned reads covering the region from 10 bases upstream to 10 bases downstream and used the joint probability distribution of basecalling QVs and pairwise joint sequencing error rates in these 21 loci as features to predict the DNA methylation level (proportion of the methylated bases at the locus). Because it is difficult to use joint probability directly as the input of a machine learning model, we characterize the distribution by combining high-order moments (**Fig. 2**): 1) first-order moment, the mean QV of each locus; 2) second-order moment, the covariance of QVs; 3) third-order moment, the coskewness of QVs; and 4) fourth-order moment, the cokurtosis of QVs. Since sequence context affects the QV and error rate, the sequences of the 21 loci are also included in the feature list. The details of feature extraction are described in the **Methods** section.

In this study, we used the DNA methylation level at each CpG locus predicted by Guppy 6.3.8 or Dorado 0.5.3 as the “known” methylation level and the features obtained from basecalling QVs and sequencing errors to train a gradient boosting regression model [24] (**Fig. 2**). The details of model training are described in the **Methods** section. Guppy [25]and Dorado [26]are toolsets provided by Oxford Nanopore for basecalling and base-modification calling. They can be used as the benchmark since the predicted locus-level DNA methylation is highly consistent with that obtained by bisulfite sequencing (the Pearson correlation coefficient (PCC) is approximately 0.95 [27,28]). By comparing hypermethylated and hypomethylated loci predicted by Guppy in the training data, all the features exhibit differences between them (**Supplementary Fig. S1**). By evaluating the accuracy of NanoFreeLunch with different features on human pangenome data, the results show that while each feature has some ability to predict DNA methylation levels, combining all the features yields the most accurate model (**Supplementary Fig. S2**).

Comparing NanoFreeLunch with benchmark methods

In this study, we adopt two independent benchmark methods. The first is bisulfite sequencing, which is a traditional NGS-based method for detecting DNA methylation. The second one is Guppy/Dorado, the raw-signal-based method provided by Oxford Nanopore, to detect base modifications from raw signals of nanopore sequencing data. NanoFreeLunch was evaluated on three independent datasets. The first dataset was obtained from the ONT open dataset released by Oxford Nanopore [28], which sequences the GM24385 (HG002) cell line by MinION with the R9.4.1 flowcell. The second dataset included nine samples from the Human Pangenome Project (HPGP) [29] sequenced by PromethION with the R9.4.1 flowcell (a part of sample HG01109 was used as the training data and excluded from evaluation) [30]. The third dataset is the Ashkenazim

trio (HG002, HG003, and HG004) sequenced by PromethION with the R10.4.1 flowcell. Each sample was sequenced at 4kHz and 5kHz sampling rates. The data are downloaded from [31]and [32].

In the first dataset, we used Guppy 6.3.8 for basecalling from the raw electrical signals of the HG002 R9.4.1 data and used NanoFreeLunch to estimate the DNA methylation level of each CpG site from the basecalled data. The bisulfite-based and raw-signal-based DNA methylation levels were downloaded from [28]. The results show that the PCCs between NanoFreeLunch and these benchmark methods are 0.89 and 0.90, respectively (**Fig. 3A** and **Fig. 3B**). In the second dataset, the basecalling results of multiple versions of Guppy (version 2.3.5, 4.2.2, and 6.3.8) are used as the input of NanoFreeLunch because these major versions implement different basecalling algorithms. Raw-signal-based DNA methylation calling was performed with Guppy 6.3.8 (**Methods**). The PCC between NanoFreeLunch and Guppy ranged from 0.87 to 0.94 (**Supplementary Fig. S3**, **Supplementary Fig. S4**, and **Supplementary Fig. S5**). We used the same basecaller version for the training and testing data. In the third dataset, we used Dorado 0.5.3 to convert the raw signals to basecalled DNA sequences and used NanoFreeLunch to estimate the DNA methylation level of each CpG site. The raw-signal-based DNA methylation levels were obtained using the modification calling mode of Dorado 0.5.3. The PCC between NanoFreeLunch and Dorado ranges from 0.89 to 0.93 (**Supplementary Fig. S6**).

Cytosine methylation is spatially correlated [32], and the overall methylation state of genomic regions is commonly used in studying the association between DNA methylation and diseases or phenotypes [33]. Therefore, we also evaluated the performance of NanoFreeLunch in estimating the methylation level of genomic regions. In this study, we used CpG islands for the evaluation.

By averaging the methylation levels of the loci in each CpG island, the PCC between the regional average methylation levels predicted by NanoFreeLunch and bisulfite sequencing was 0.99 (**Fig. 3C**). By comparing NanoFreeLunch with Guppy/Dorado, the PCC ranged from 0.97 to 0.99 (**Fig. 3D, Supplementary Fig. S7, Supplementary Fig. S8, Supplementary Fig. S9, and Supplementary Fig. S10**). These results demonstrate that NanoFreeLunch can restore DNA methylation accurately without using raw signals.

The impact of flowcell type and basecaller version on the accuracy of NanoFreeLunch

Flowcell type and basecaller version have an impact on the results of NanoFreeLunch since they might produce different errors and QV patterns. We compared the accuracy of NanoFreeLunch for different flowcell types (R9.4.1 and R10.4.1) and basecaller versions (Guppy 2.3.5, 4.2.2, and 6.3.8 for R9.4.1 and Dorado 0.5.3 for R10.4.1). The results show that the differences in accuracy are limited. The maximum accuracy difference is 0.04 according to a comparison of the Guppy 4.2.2 and Guppy 6.3.8 data (**Supplementary Fig. S11**). Therefore, despite the significant differences in the sequencing error rate, flowcell type and basecaller version have limited impacts on the accuracy of NanoFreeLunch.

Estimating the DNA methylation level of imprinting control regions with NanoFreeLunch

A key feature of NanoFreeLunch is the quantitative detection of DNA methylation, which means that it can report the percentage of methylated bases for each genomic locus without using raw signals. Partially methylated regions such as imprinting control regions (ICRs) that regulate gene imprinting play a critical role in human development and diseases [1]. To evaluate the performance of NanoFreeLunch in detecting partially methylated genomic regions, we used 14 ICRs with

hypermethylated DNA from only one of the parents confirmed by multiple previous studies [34,35] and calculated the average NanoFreeLunch-predicted methylation level in these regions using samples from the HGPG dataset (R9.4.1 flowcell and basecalling with Guppy) and Ashkenazim trio dataset (R10.4.1 flowcell and basecalling with Dorado). Utilizing basecalling outcomes from either Guppy 6.3.8 or Guppy 2.3.5 as input, all 14 ICRs exhibited a median methylation level within the range of 0.25 to 0.75, which encapsulates the middle 50% of the [0,1] range (**Fig. 4 and Supplementary Fig. S12**). Likewise, employing basecalling outcomes from Guppy 4.2.2 or Dorado 0.5.3 yields comparable results, with 13 out of the 14 ICRs displaying a median methylation level within the 0.25 to 0.75 range (**Supplementary Fig. S13** and **Supplementary Fig. S14**). When examining the trimmed means of predicted ICR methylation levels, NanoFreeLunch prediction with basecalling using Guppy 2.3.5, Guppy 4.2.2, Guppy 6.3.8, and Dorado 0.5.3 revealed values of 0.54, 0.59, 0.50, and 0.57, respectively. Correspondingly, the associated trimmed standard variances are 0.09, 0.08, 0.06, and 0.13 (**Methods**). As a benchmark, when considering methylation levels estimated by the raw-signal-based methods Guppy 6.3.8 and Dorado 0.5.3, all 14 out of 14 ICRs exhibited a median methylation level within the 25% to 75% range (**Supplementary Fig. S15** and **Supplementary Fig. S16**). The trimmed means for the predicted ICR methylation levels were 0.54 for Guppy 6.3.8 and 0.50 for Dorado 0.5.3, with corresponding standard variances of 0.07 for Guppy 6.3.8 and 0.08 for Dorado 0.5.3. These results demonstrate that the DNA methylation levels of ICR predicted by NanoFreeLunch are concentrated in the middle segment of the [0,1] range, exhibiting mean and variance characteristics consistent with those of raw-signal-based methodologies.

DNA methylation levels estimated by NanoFreeLunch are consistent with histone modification and DNase sensitivity

To further evaluate the reliability of NanoFreeLunch, we calculated the consistency between the DNA methylation level predicted by NanoFreeLunch and other epigenomic markers, including histone modification and DNase sensitivity, obtained from the ENCODE project [36,37] (**Methods**). We obtained the average CpG methylation level predicted by NanoFreeLunch for H3K9me3 regions and the overlaps between H3K4me3 regions and DNase hypersensitive regions (**Methods**). H3K9me3 is the histone mark of regions with repressed transcription and DNA hypermethylation, while H3K4me3 and DNase hypersensitivity are associated with activated transcription and DNA hypomethylation [38]. In the HPGP dataset, which was sequenced using the R9.4.1 flowcell, the DNase hypersensitive regions marked by H3K4me3 exhibited low methylation levels, as predicted by NanoFreeLunch. The average median methylation levels for these regions were 13.4%, 6.6%, and 8.6% when the data were basecalled by Guppy versions 6.3.8, 4.2.2, and 2.3.5, respectively. In contrast, the H3K9me3 regions exhibit high methylation levels. Specifically, the average median methylation levels for these regions were 83.7%, 86.1%, and 83.9%, respectively (**Fig. 5**, **Supplementary Fig. S17**, and **Supplementary Fig. S18**). NanoFreeLunch-predicted methylation levels are also consistent with those estimated by the raw-signal-based method Guppy (**Supplementary Fig. S19**). According to the Ashkenazim trio data, which were sequenced by the R10.4.1 flowcell, the DNase hypersensitive regions with H3K4me3 had an average median NanoFreeLunch-predicted methylation level of 2.5%, and the H3K9me3 regions had an average median methylation level of 88.3% (**Supplementary Fig. S20**), consistent with the Dorado-predicted methylation levels (**Supplementary Fig. S21**). These results

demonstrate that DNA methylation levels estimated by NanoFreeLunch are consistent with histone modification and DNase sensitivity.

Discussion

Nanopore sequencing provides an unprecedented opportunity for the data mining of DNA methylation data by reutilizing and integrating existing nanopore sequencing data. A major obstacle is that the existing methods for detecting DNA methylation require raw signals from nanopore sequencing as the input, but most data do not include raw signals due to the difficulty of storing, processing, and sharing the raw signal files. In this work, we address this challenge by developing a novel method termed NanoFreeLunch that can detect DNA methylation quantitatively from basecalled FASTQ files without raw signals. With the ability to leverage rapidly accumulating nanopore sequencing data, NanoFreeLunch provides unprecedented opportunities for large-scale construction of epigenomes, even from datasets not originally designed for DNA methylation studies.

This study demonstrated the significance and effectiveness of NanoFreeLunch for detecting hypermethylated, hypomethylated, and partially methylated regions whose methylation status is precisely regulated. These results show that the restored DNA methylation from basecalled FASTQ files when the raw signals are lost is accurate enough to provide biologically meaningful insights.

Major version changes in the basecaller or flowcell might have an impact on the results of NanoFreeLunch since they change the error pattern and QV distribution. NanoFreeLunch should be trained on the matched version to achieve the best accuracy. We provide pre-trained models for versions 2.3.5, 4.2.2, and 6.3.8 of Guppy on flowcell R9.4.1 and Dorado 0.5.3 on the R10.4.1

flowcell in this work, but NanoFreeLunch is flexible and has a CLI (command line interface) for users to train their models.

In this study, we used HAC mode for basecalling, which is commonly used and recommended mode since it has a good balance between speed and accuracy. However, basecalling mode might affect methylation detection of NanoFreeLunch since different modes might have different error and QV patterns. Using models trained on chromosome 10 (HG01109 for R9, HG002 for R10) and tested on chromosome 6 (HG01243 for R9, HG003 for R10), we found that FAST mode yielded slightly higher accuracy than HAC mode, while SUP mode yielded slightly lower accuracy for both R9 and R10 data (**Supplementary Fig. S22**). This observed dependency, coupled with the complex and non-monotonic relationship between basecalling accuracy and NanoFreeLunch accuracy, indicates that the specific error and QV characteristics introduced by different basecalling modes may have complex and hard-to-interpret effects on the features used by NanoFreeLunch for methylation prediction.

The method presented in this study offers a versatile and adaptable framework with potential for expansion. While the focus of this work was on detecting 5mC in the CpG context, the same framework can be applied to other types of base modifications by using different training data. NanoFreeLunch provides a CLI for training models with customized data, allowing further study to extend its ability to detect various types of base modifications beyond 5mC.

Conclusions

NanoFreeLunch offers a distinct solution to a significant challenge in the field: the reutilization of nanopore sequencing data for DNA methylation detection, particularly in the absence of raw-

signal files in public databases. By introducing a novel strategy that accounts for sequencing error and base QV, NanoFreeLunch enables reliable quantitative detection of DNA methylation. This new method opens avenues for uncovering novel biological insights through large-scale integration of nanopore sequencing for DNA methylation detection.

Methods

Extracting the features used by NanoFreeLunch

Assuming that there are n reads fully covering the [-10, 10] regions of a genomic locus of interest (**Fig. 2**), we denote R_{ij} as the sequenced base of read j at genomic locus i, T_i as the reference genome base at locus i, and Q_{ij} as the base QV of read j at locus i, where $i = -10, \dots, 10$, $j = 1, \dots, n$. The mean QV vector is defined as

$$mean(Q) = [M_i]_{i \in [-10,10]}$$

where $M_i = \frac{1}{n}\sum_{j=1}^{n} Q_{ij}$ and $mean(Q)$ is an 1×21 row vector. The QV covariance matrix is defined as

$$cov(Q) = \left[V_{pq}\right]_{p,q \in [-10,10]}$$

where $V_{pq} = \frac{1}{n-1}\sum_{j=1}^{n}(Q_{pj} - M_p)(Q_{qj} - M_q)$ and $cov(Q)$ is an 21×21 matrix. The QV coskewness matrix is defined as

$$coskewness(Q) = \left[S_{pq}\right]_{p,q \in [-10,10]}$$

where $S_{pq} = \frac{\sum_{j=1}^{n}\left(Q_{pj} - M_p\right)^2 (Q_{qj} - M_q)}{n\sigma_p^2\sigma_q}$, σ_p and σ_q are standard variances. $coskewness(Q)$ is an 21×21 matrix. The QV cokurtosis matrix is defined as

$$coskurtosis(Q) = [K_{pq}]_{p,q \in [-10,10]}$$

where $K_{pq} = \frac{\sum_{j=1}^{n}(Q_{pj}-M_p)^2(Q_{qj}-M_q)^2}{n\sigma_p^2\sigma_q^2}$, σ_p and σ_q are standard variances. $coskurtosis(Q)$ is an 21×21 matrix. The pairwise joint sequencing error rate is defined as

$$error(R) = [E_{stpq}]_{s,t \in [A,C,G,T,D],p,q \in [-10,10]}$$

where $E_{stpq} = \frac{1}{n}\sum_{j=1}^{n} I(R_{pj} = s)I(R_{qj} = t)I(T_p \neq s)I(T_q \neq t)$, $I(\cdot)$ is the indicator function, D is the deletion, and $error(R)$ is an $5 \times 5 \times 21 \times 21$ 4-dimensional array. The sequence context is binary coded with two digits for each base as follows:

$$context = [C_i]_{i \in [-10,10]}$$

where $C_i = 00$ if $T_i = "A"$; $C_i = 01$ if $T_i = "C"$; $C_i = 10$ if $T_i = "G"$; and $C_i = 11$ if $T_i = "T"$.

Data preparation and preprocessing

The FAST5 files of R9 (abbreviated as R9.4.1 flowcell) data were downloaded from [28] and [29] for the HG002 dataset and HPGP (Human Pangenome Project) dataset, respectively. The POD5/FAST5 files of the R10.4.1 flowcell (abbreviated as R10) Ashkenazim Trio data are downloaded from [31] and [39] for the 4kHz and 5kHz flowcells, respectively. For the R9 data, basecalling was performed using the parameters *"guppy_basecaller -x "cuda:all" --compress_fastq --bam_out -r -c dna_r9.4.1_450bps_hac.cfg"*, and base-level CpG methylation calling was performed using *"guppy_basecaller -x "cuda:all" --compress_fastq --bam_out -r -c dna_r9.4.1_ 450bps_modbases_5mc_cg_hac.cfg"* with Guppy 6.3.8. For the R10 data, basecalling was performed using the following parameters: "dorado basecaller --reference reffile hac infile > bamfile", and

base-level CpG methylation calling was performed using "dorado basecaller --reference reffile hac,5mCG_5hmCG infile > bamfile" with Dorado 0.5.3. Reads mapping was performed using Guppy/Dorado along with basecalling by providing GRCh38 obtained from s3://ont-open-data/gm24385_mod_2021.09/refs as the reference genome. DNA methylation calling for genomic loci was performed using modbam2bed (version 0.6.3) downloaded from [40] with the parameters "modbam2bed -e -m 5mC --cpg". Basecalling results obtained using Guppy 2.3.5 and Guppy 4.2.2 were downloaded from [29] for 7 samples—HG01109, HG01243, HG02055, HG02080, HG02723, HG03098, and HG03492—and the other samples did not include basecalling results obtained with Guppy 2.3.5 or Guppy 4.2.2. The basecalled reads were mapped to GRCh38 using minimap2 [41] (version 2.24) with the parameters "minimap2 -ax map-ont --secondary=no --sam-hit-only -L", and the mapped reads were sorted, indexed, and filtered using samtools [42] (version 1.16.1) with the parameters "samtools sort", "samtools index", and "samtools view -h -b -F 4079". The methylation calling results of whole-genome bisulfite sequencing used as the benchmark in this study were downloaded from EPI2ME Desktop (s3://ont-open-data/gm24385_mod_2021.09/bisulphite/cpg/CpG.gz.bismark.zero.cov.gz).

Training the model of NanoFreeLunch

All the analyses for detecting DNA methylation without raw signals used version 0.24.0 of NanoFreeLunch. For the R9 data, we used chromosome 10 of sample HG01109 from the HPGP dataset and sample HG002 from ONT Open Data as the training data for the R9 PromethION data and R9 MinION data, respectively. For the R10 data, we used chromosome 10 of sample HG002 from ONT Open Data for the R10 4kHz and R10 5kHz data. The mapped reads were converted to the features described in **Extracting the features used by NanoFreeLunch** by "*nfl prepdata -r -*

p -f --chr chrname bamfile reffile locifile", where *bamfile* is the mapped reads, *reffile* is the FASTA file of the reference genome, and *locifile* is the CpG loci reported by modbam2bed. In the *locifile*, loci with depth lower than 10x or score less than 800 were removed. This command also converts matrices and high-dimensional arrays to vectors so that they can be used as the input of the gradient boosting model. The DNA methylation level at each CpG locus predicted by modbam2bed was logit-transformed with the following formula and used as the response of the model.

$$y' = \begin{cases} -\alpha, & y = 0 \\ \log(y) - \log(1 - y), & 0 < y < 1 \\ \alpha, & y = 1 \end{cases}$$

where $\alpha = 10^{-3}$ in this study. The model is trained with the parameters "nfl train --alpha 1e-3". The core gradient boosting model is implemented by the Julia [43] wrapper of XGBoost (version 1.5.2) [24,44]. The learning rate, "*eta*", is 0.1, the number of trees, "*num_round*", is 1500, the maximal tree depth, "*max_depth*", is 8, and the other parameters are set to their defaults.

Estimating DNA methylation levels

We used the trained model to predict the DNA methylation level by the command "nfl predict" with default parameters. This NanoFreeLunch command internally calls the trained XGBoost model with the input features. The DNA methylation levels in the forward and backward strands of the same CpG site were averaged.

Estimating the DNA methylation level of CpG islands

The GRCh38-based genomic coordinates of CpG islands were downloaded from the UCSC genome browser [45] by selecting "Regulation" in "Group", "CpG islands" in "Track", and "GRCh38"

in "Assembly". The average methylation level of a CpG island is estimated by the trimmed mean of the CpG methylation level by removing data points outside of the [median – variance, median + variance] range. The command is "*nfl get-range-trimmean -f*".

Estimating the DNA methylation level of ICRs

The ICRs are obtained from regions in Table 1 of (Jima D. et al 2022) [35] with a "#" mark and filtered by retaining the regions that can also be found in Table 1 of (Skaar D. et al 2012)[34]. The genomic coordinates in (Skaar D. et al 2012) are based on GRCh37, and we used LiftOver [46] to convert them to GRCh38-based coordinates. Similar to the method in **Estimating DNA methylation level of CpG islands**, the DNA methylation levels of the ICRs are estimated by the "*nfl get-range-trimmean -f*". The mean and variance of ICR methylation levels are calculated using trimmed statistics, specifically by excluding the highest and lowest 5% of data points.

Estimating DNA methylation levels in regions with different histone marks

We used the histone mark data and DNase sensitivity data of GM12878 from the ENCODE project [36,37] as the reference epigenome in this study. The H3K4me3 peak regions were downloaded from [47]. The H3K9me3 peak regions were downloaded from [48]. The DNase-hypersensitive regions were downloaded from [49]. "H3K4m3 + DNase" regions are the overlapping regions between H3K4me3 peak regions and DNase hypersensitive regions. Similar to the method in **Estimating DNA methylation level of CpG islands**, the average methylation level of a region is estimated by "*nfl get-range-trimmean-f*".

Assessing the relative importance of different features

We used the region 50,000,000–60,000,000 on chromosome 10 of sample HG01109 to assess the impact of DNA methylation on these features. The features are extracted from the aligned reads as described in **Extracting the features used by NanoFreeLunch** for the 106,209 CpG loci in this region. The loci with DNA methylation levels predicted by Guppy less than 0.1 and greater than 0.9 were regarded as unmethylated loci and methylated loci, respectively. The differences between the average features of methylated loci and unmethylated loci are shown in **Supplementary Fig. S1**. The model accuracy using each feature or combination of features was evaluated using chromosome 6 of the nine samples from the human pangenome project. The average accuracies are shown in **Supplementary Fig. S2**.

Statistics of the SRA records

We performed an advanced search of the SRA database [20] and selected "oxford nanopore" in “Platform” to obtain the total number of ONT records, denoted as N_{total}. Similarly, we selected "oxford nanopore" in “Platform” and "filetype nanopore" in “Properties” to obtain the number of ONT records with the raw FAST5/POD5 files, denoted as N_{raw}. The proportion of records consisting of raw FAST5/POD5 files was calculated by N_{raw}/N_{total}. To obtain the statistics for each year in **Fig. 1**, we repeat the process by setting “Publication Date” ranging from 2015 to 2023.

Availability of source code and requirements

Project name: NanoFreeLunch

Project homepage: https://gitee.com/zhixingfeng/NanoFreeLunch.jl [50]

Project demo: https://gitee.com/zhixingfeng/nfl-demo/tree/main/demo [51]

biotoolsID: nanofreelunch

RRID: SCR_027196

Operating system(s): Linux for x86_64 machines.

Programming languages: Julia

License: GNU GPL v3

Data Availability

The ONT open dataset's MinION R9.4.1 flowcell data were obtained from EPI2ME Desktop [28]. The PromethION R9.4.1 flowcell data released by Human Pangenome Project were obtained from Github[29]. The ONT open dataset's PromethION R10.4.1 flowcell 4kHz and 5kHz data were obtained from EPI2ME Desktop [31] and [39] respectively.

The histone mark data and DNase sensitivity data of GM12878 were obtained from ENCODE project. Specifically, the H3K4me3 peak regions were downloaded from EPI2ME Desktop [47]. The H3K9me3 peak regions were downloaded from EPI2ME Desktop [48]. The DNase-hypersensitive regions were downloaded from EPI2ME Desktop [49]. There are additional demo data hosted in NanoFreeLunch [52].

Competing interests

Z.F. is listed as an author on a patent application related to this work. The other authors declare no conflicts of interest.

Funding

This work is supported by the National Natural Science Foundation of China (No. 32370695 and No. 32470684) and the National Key R&D Program of China (No. 2022YFC2703400).

Authors' contributions

Z.F., and H.F., designed the project. Z.F. invented the algorithms of NanoFreeLunch and developed the software. Z.F., C.Z., S.J., J.N. and H.F. designed and evaluated the NanoFreeLunch. C.Z. and H.F. modeled the context effect. Z.F. wrote the manuscript with the help of H.F.

Acknowledgments

The computations in this work were run on the π 2.0 and Siyuan Mark-I clusters supported by the Center for High Performance Computing at Shanghai Jiao Tong University and CFFF platform of Fudan University.

References

1. Monk D, Mackay DJG, Eggermann T, et al. Genomic imprinting disorders: lessons on how genome, epigenome and environment interact. Nat Rev Genet 2019; 20:235–248

2. Sharp AJ, Stathaki E, Migliavacca E, et al. DNA methylation profiles of human active and inactive X chromosomes. Genome Res 2011; 21:1592–1600

3. Jones PA, Gonzalgo ML. Commentary Altered DNA methylation and genome instability: A new pathway to cancer? Proc. Natl. Acad. Sci 1997; 94:2103–2105

4. Caramaschi D, Neumann A, Cardenas A, et al. Meta-analysis of epigenome-wide associations between DNA methylation at birth and childhood cognitive skills. Mol Psychiatry 2022; 27:2126–2135

5. Wang G, Wang B, Yang P. Epigenetics in Congenital Heart Disease. J Am Heart Assoc 2022; 11:e025163

6. Ehrlich M. DNA methylation in cancer: too much, but also too little. Oncogene 2002; 21:5400–5413

7. Min JL, Hemani G, Hannon E, et al. Genomic and phenotypic insights from an atlas of genetic effects on DNA methylation. Nat Genet 2021; 53:1311–1321

8. Breitling LP, Yang R, Korn B, et al. Tobacco-smoking-related differential DNA methylation: 27K discovery and replication. Am J Hum Genet 2011; 88:450–457

9. Péron S, Laffleur B, Denis-Lagache N, et al. Quantitative Sequencing of 5-Methylcytosine and 5-Hydroxymethylcytosine at Single-Base Resolution. Science 2012; 336:931–934

10. Yu M, Hon GC, Szulwach KE, et al. Base-resolution analysis of 5-hydroxymethylcytosine in the mammalian genome. Cell 2012; 149:1368–1380

11. Kurdyukov S, Bullock M. DNA methylation analysis: Choosing the right method. Biology (Basel) 2016; 5:

12. Simpson JT, Workman RE, Zuzarte PC, et al. Detecting DNA cytosine methylation using nanopore sequencing. Nat Methods 2017; 14:407–410

13. Yuen ZWS, Srivastava A, Daniel R, et al. Systematic benchmarking of tools for CpG methylation detection from nanopore sequencing. Nat Commun 2021; 12:3438

14. Rand AC, Jain M, Eizenga JM, et al. Mapping DNA methylation with high-throughput nanopore sequencing. Nat Methods 2017; 14:411–413

15. Bonet J, Chen M, Dabad M, et al. DeepMP: A deep learning tool to detect DNA base modifications on Nanopore sequencing data. Bioinformatics 2022; 38:1235–1243

16. McIntyre ABR, Alexander N, Grigorev K, et al. Single-molecule sequencing detection of N6-methyladenine in microbial reference materials. Nat Commun 2019; 10:579

17. Liu Q, Georgieva DC, Egli D, et al. NanoMod: A computational tool to detect DNA modifications using Nanopore long-read sequencing data. BMC Genomics 2019; 20:78

18. Liu Q, Fang L, Yu G, et al. Detection of DNA base modifications by deep recurrent neural network on Oxford Nanopore sequencing data. Nat Commun 2019; 10:2449

19. Ni P, Huang N, Zhang Z, et al. DeepSignal: Detecting DNA methylation state from Nanopore sequencing reads using deep-learning. Bioinformatics 2019; 35:4586–4595

20. National Center for Biotechnology Information (NCBI). Sequence Read Archive (SRA). https://www.ncbi.nlm.nih.gov/sra. 2023;

21. de Coster W, Weissensteiner MH, Sedlazeck FJ. Towards population-scale long-read sequencing. Nat Rev Genet 2021; 22:572–587

22. NIH. Total size of the SRA database. https://dpcpsi.nih.gov/council/sradwg. 2023;

23. Beyter D, Ingimundardottir H, Oddsson A, et al. Long-read sequencing of 3,622 Icelanders provides insight into the role of structural variants in human diseases and other traits. Nat Genet 2021; 53:779–786

24. Chen T, Guestrin C. XGBoost: A scalable tree boosting system. Proceedings of the ACM SIGKDD International Conference on Knowledge Discovery and Data Mining 2016; 13-17-August-2016:785–794

25. Oxford Nanopore Technologies. Guppy. https://nanoporetech.com/community. 2023;

26. Oxford Nanopore Technologies. Dorado. https://github.com/nanoporetech/dorado. 2023;

27. Ni P, Xu J, Zhong Z, et al. DNA 5-methylcytosine detection and methylation phasing using PacBio circular consensus sequencing. Nat Commun. 2023. https://doi.org/10.1038/s41467-023-39784-9

28. Wright C. Detection of 5-methylcytosine modification in GM24385. https://labs.epi2me.io/gm24385-5mc. 2023;

29. Human PanGenomics Project. Data from the Human PanGenomics Project. https://github.com/human-pangenomics/hpgp-data. 2023;

30. Shafin K, Pesout T, Lorig-Roach R, et al. Nanopore sequencing and the Shasta toolkit enable efficient de novo assembly of eleven human genomes. Nat Biotechnol 2020; 38:1044–1053

31. Wright C. Genome in a Bottle Ashkenazi Trio with Ligation Sequencing Kit V14. https://labs.epi2me.io/askenazi-kit14-2022-12. 2023;

32. Eckhardt F, Lewin J, Cortese R, et al. DNA methylation profiling of human chromosomes 6, 20 and 22. Nat Genet 2006; 38:1378–1385

33. Rakyan VK, Down TA, Balding DJ, et al. Epigenome-wide association studies for common human diseases. Nat Rev Genet 2011; 12:529–541

34. Skaar D, Li Y, Bernal A, et al. The Human Imprintome: Regulatory Mechanisms, Methods of Ascertainment, and Roles in Disease Susceptibility. ILAR J 2012; 53:341–58

35. Jima DD, Skaar DA, Planchart A, et al. Genomic map of candidate human imprint control regions: the imprintome. Epigenetics 2022; 17:1920–1943

36. Dunham I, Kundaje A, Aldred SF, et al. An integrated encyclopedia of DNA elements in the human genome. Nature 2012; 489:57–74

37. Abascal F, Acosta R, Addleman NJ, et al. Expanded encyclopaedias of DNA elements in the human and mouse genomes. Nature 2020; 583:699–710

38. Roadmap Epigenomics Consortium, Kundaje A, Meuleman W, et al. Integrative analysis of 111 reference human epigenomes. Nature 2015; 518:317–329

39. Talenti A. Sequencing Genome in a Bottle samples. https://labs.epi2me.io/giab-2023.05. 2023;

40. Epi2me Labs. modbam2bed. https://github.com/epi2me-labs/modbam2bed. 2023;

41. Li H. Minimap2: Pairwise alignment for nucleotide sequences. Bioinformatics 2018; 34:3094–3100

42. Danecek P, Bonfield JK, Liddle J, et al. Twelve years of SAMtools and BCFtools. Gigascience. 2021,10(2):giab008. doi: 10.1093/gigascience/giab008

43. Julia Language. The Julia Programming Language. https://julialang.org. 2023;

44. XGBoost. XGBoost.jl. https://github.com/dmlc/XGBoost.jl. 2023;

45. UCSC Genome Browser. UCSC Table Browser. https://genome.ucsc.edu/cgi-bin/hgTables. 2024;

46. UCSC Genome Browser. liftOver. https://genome.ucsc.edu/cgi-bin/hgLiftOver. 2023;

47. ENCODE Project Consortium. H3K4me3 peak regions. https://www.encodeproject.org/files/ENCFF320OGZ/@@download/ENCFF320OGZ.bed.gz. 2023;

48. ENCODE Project Consortium. H3K9me3 peak regions. https://www.encodeproject.org/files/ENCFF725UFY/@@download/ENCFF725UFY.bed.gz. 2023;

49. ENCODE Project Consortium. DNase-hypersensitive regions. https://www.encodeproject.org/files/ENCFF759OLD/@@download/ENCFF759OLD.bed.gz. 2023;

50. Feng Z. NanoFreeLunch.jl: Detecting DNA methylation from ONT data without raw-signals. https://gitee.com/zhixingfeng/NanoFreeLunch.jl. 2025;

51. Feng Z. Demo code of NanoFreeLunch. https://gitee.com/zhixingfeng/nfl-demo. 2025;

52. Feng Z. The demo data of NanoFreeLunch. https://doi.org/10.57760/sciencedb.19940. 2025;

53. Integrative Genomics Viewer. https://software.broadinstitute.org/software/igv. 2023;

FIGURES

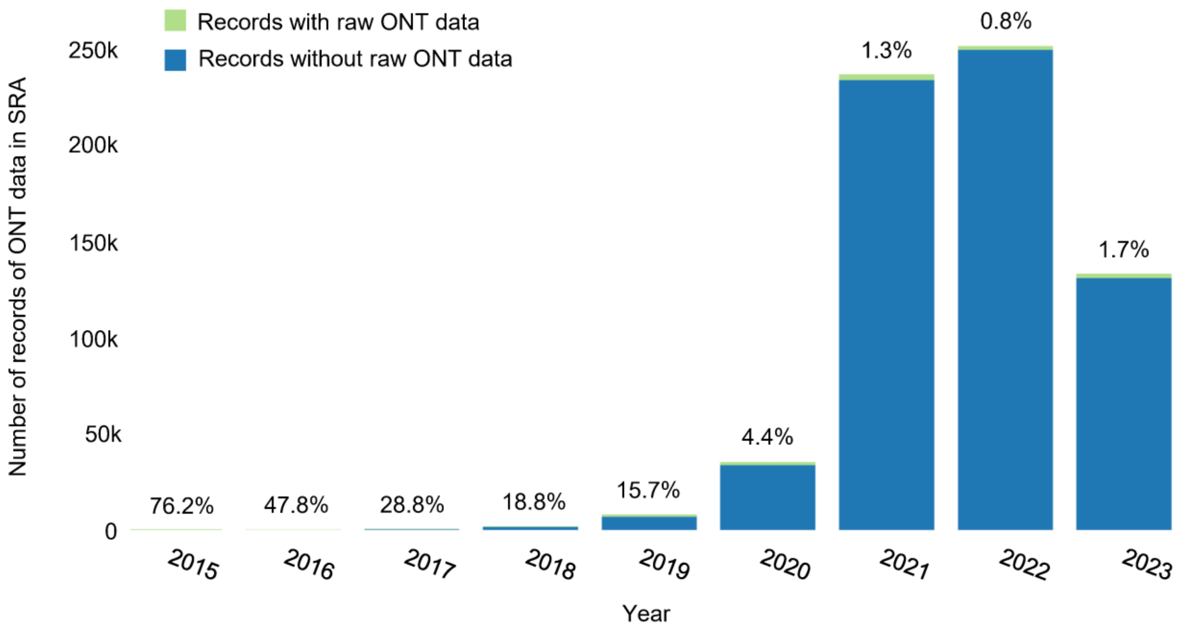

Fig. 1. The number of records of nanopore sequencing data in the SRA database each year. The height of each bar represents the number of records. The records with or without raw signal files are represented using different colors. The percentage on the top of each bar is the ratio of records with raw signals. The bars for the years 2015 and 2016 appear barely visible due to the limited amount of released data during those periods.

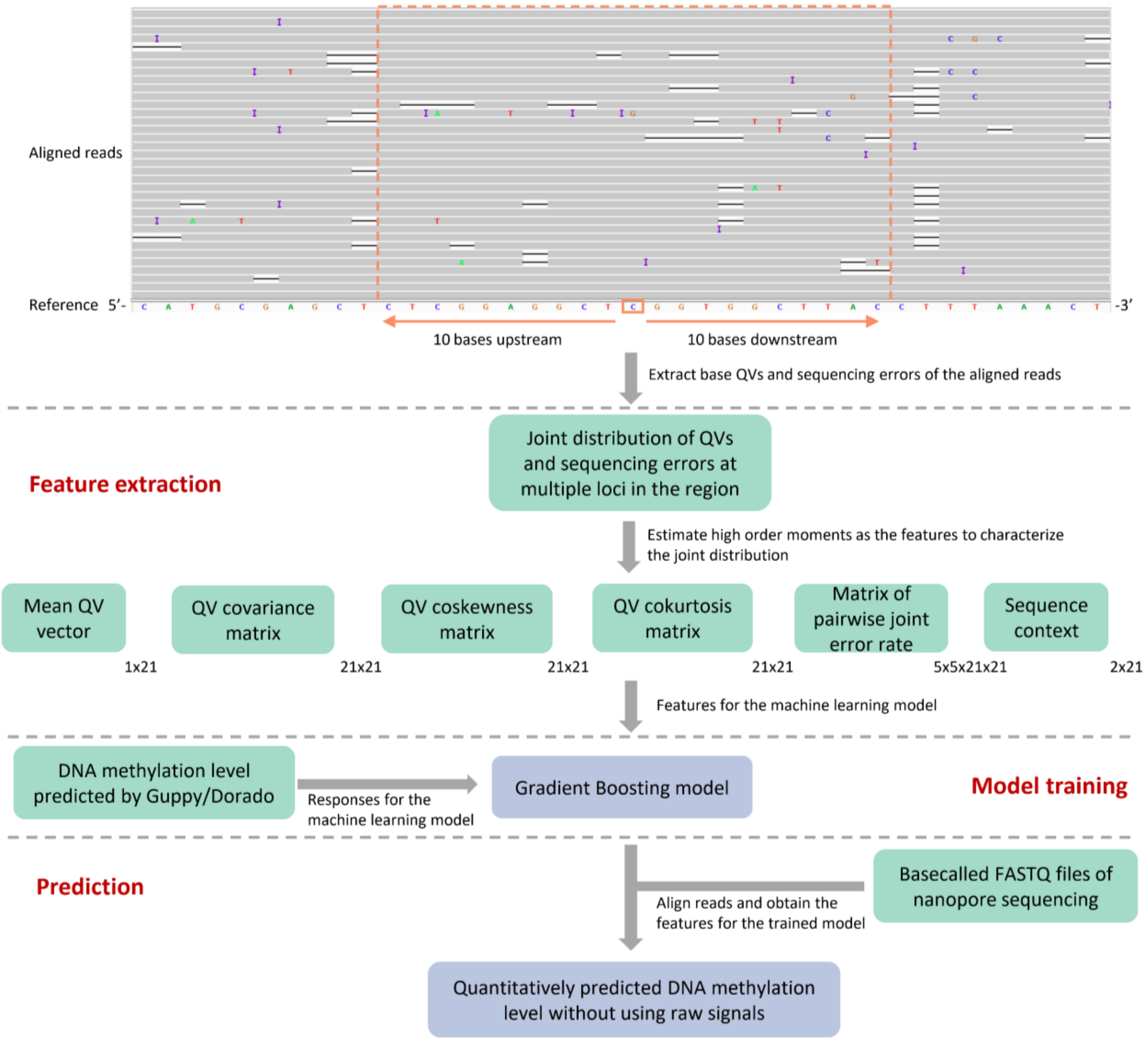

Fig. 2. The workflow of NanoFreeLunch. There are three major components in NanoFreeLunch. The first component, feature extraction, constructs the features from aligned reads of potentially methylated loci. The second component, model training, utilizes the extracted features and DNA methylation levels predicted by Guppy or Dorado to train a boosting model. The third component, prediction, leverages the trained model to predict DNA methylation levels from the features extracted from the aligned reads. The aligned reads are displayed in an IGV snapshot. IGV stands for the Integrative Genomics Viewer [53].

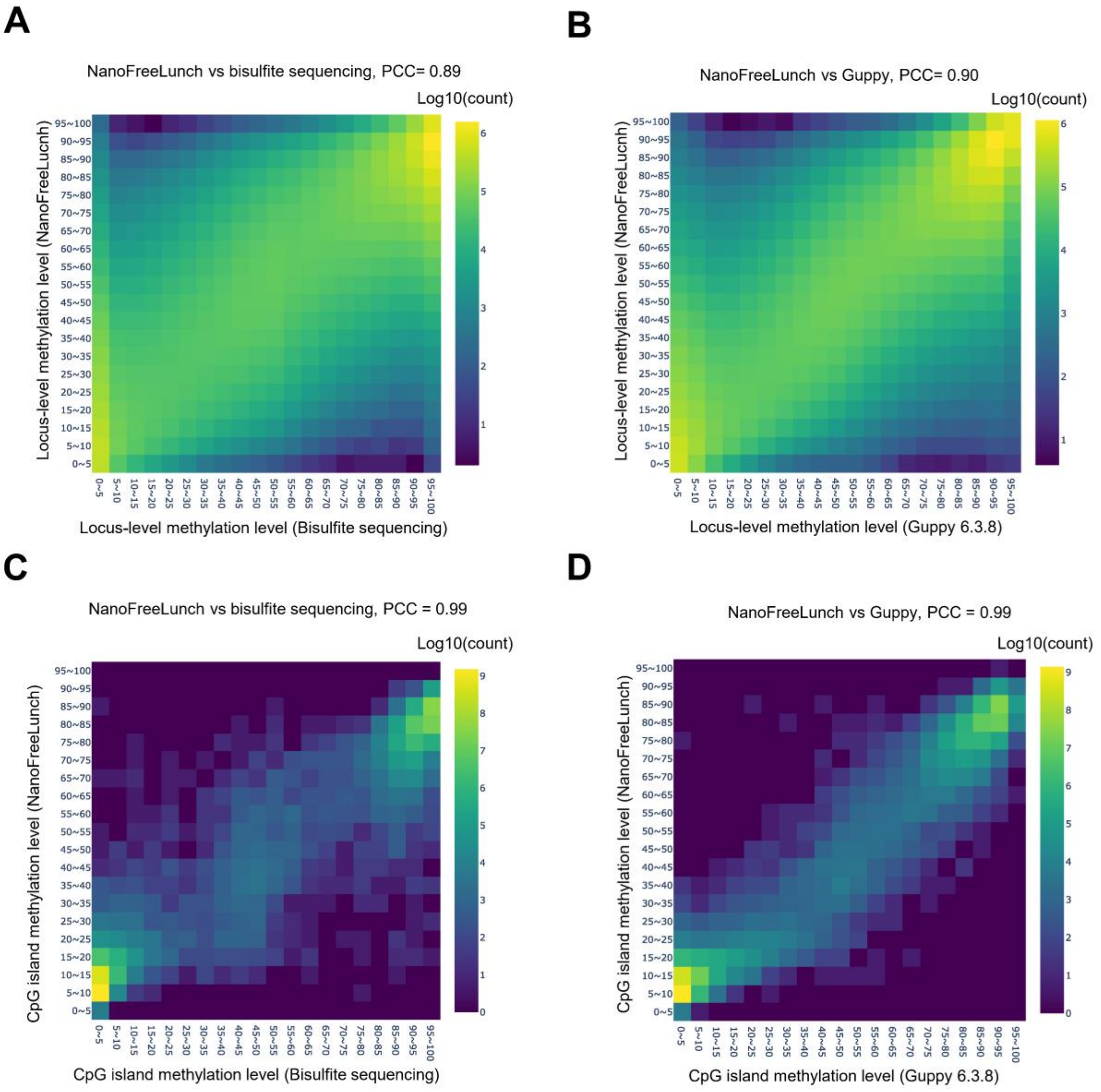

Fig. 3. Performance evaluation of NanoFreeLunch. A-B, Comparing the locus-level methylation levels estimated by NanoFreeLunch with bisulfite sequencing and Guppy 6.3.8 in the HG002 dataset. **C-D,** Comparing the average methylation level of CpG islands estimated by NanoFreeLunch with bisulfite sequencing and Guppy 6.3.8 in the HG002 dataset. The predicted DNA methylation levels were segmented into 20 bins of equal size ranging from 0% to 100%. The color of each bin represents the base-10 logarithm transformation of the number of loci or regions within the bin. PCC denotes the Pearson correlation coefficient.

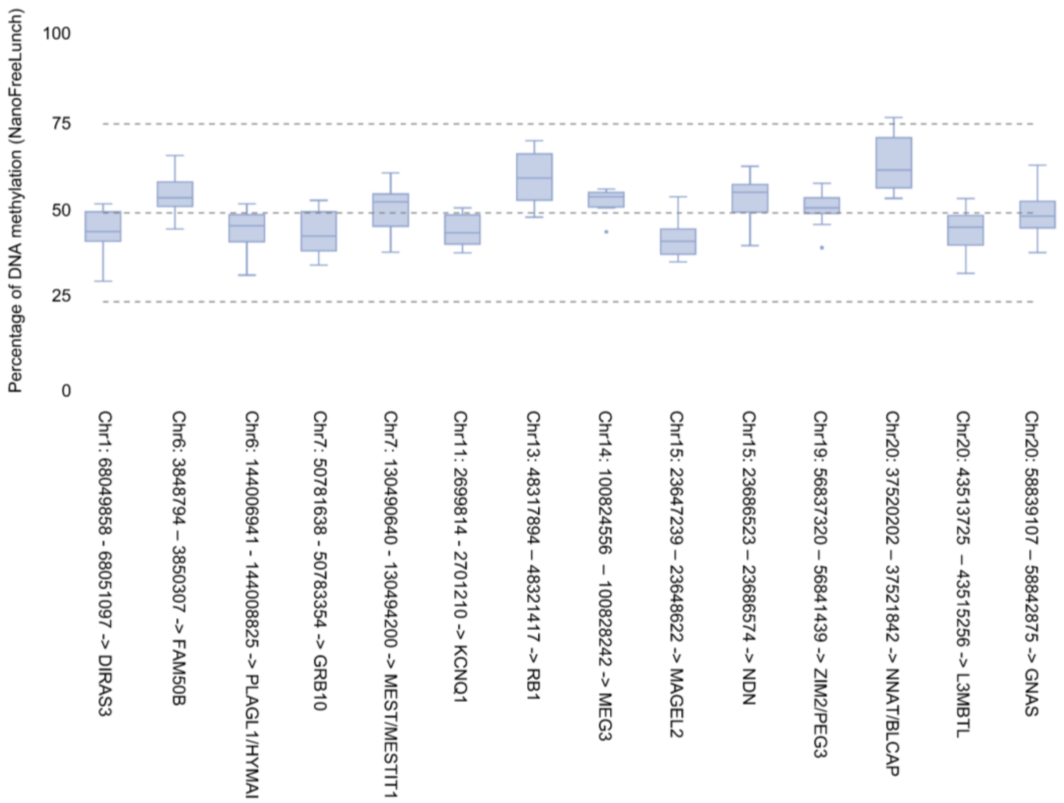

Fig. 4. The average DNA methylation level estimated by NanoFreeLunch in ICRs (Imprinting Control Regions). Each boxplot illustrates the distribution of the average DNA methylation levels of ICRs predicted by NanoFreeLunch using human pangenome data. The line in each box represents the median. The lower and upper bounds of the box correspond to the first (Q1) and third (Q3) quartiles, respectively. The lower fence is determined as the last sample point below 1.5 times the interquartile range (IQR), calculated as Q3 minus Q1. Similarly, the upper fence is identified as the last sample point above 1.5 times the IQR. In the x-axis, the region on the left of "->" is the genomic region of the ICR on GRCh38, and the gene symbol on the right is the putative gene associated with the ICR. The ICRs and their associated genes are obtained from previous publications [34,35]. The basecalling results of Guppy 6.3.8 are used as the input of NanoFreeLunch.

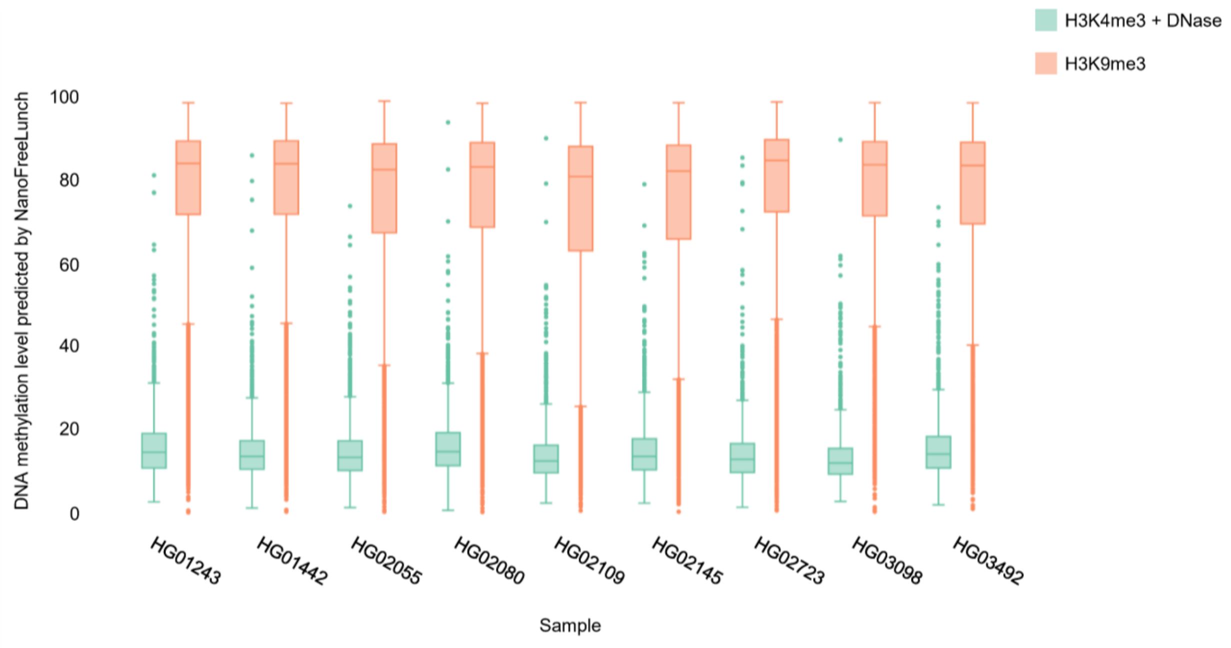

Fig. 5. Comparing DNA methylation level predicted by NanoFreeLunch with other epigenetic markers. Each boxplot depicts the distribution of average DNA methylation levels in H3K9me3 regions or DNase hypersensitive regions marked by H3K4me3, predicted by NanoFreeLunch using human pangenome data. Different colors represent distinct regions. The line in each box represents the median. The lower and upper bounds of the box correspond to the first (Q1) and third (Q3) quartiles, respectively. The lower fence is determined as the last sample point below 1.5 times the interquartile range (IQR), calculated as Q3 minus Q1. Similarly, the upper fence is identified as the last sample point above 1.5 times the IQR. The histone modification and DNase sensitivity are obtained from ENCODE [36,37]. The basecalling results of Guppy 6.3.8 are used as the input of NanoFreeLunch.

Supplementary figures of “Quantitative Detection of DNA Modifications from Nanopore Sequencing Data without Raw Signals”

Zhixing Feng[1,*], Chenxi Zhang[2], Shuo Jin[2], Jiale Niu[2], and Huijuan Feng[2,*]

[1] Department of Clinical Genetics, Xinhua Hospital affiliated to Shanghai Jiao Tong University School of Medicine, Shanghai, 200092, China

[2] Department of Computational Biology, School of Life Sciences, Fudan University, Shanghai 200438, China

* To whom correspondence should be addressed. Email: fengzhixing@shsmu.edu.cn.

Correspondence may also be addressed to huijuanfeng@fudan.edu.cn.

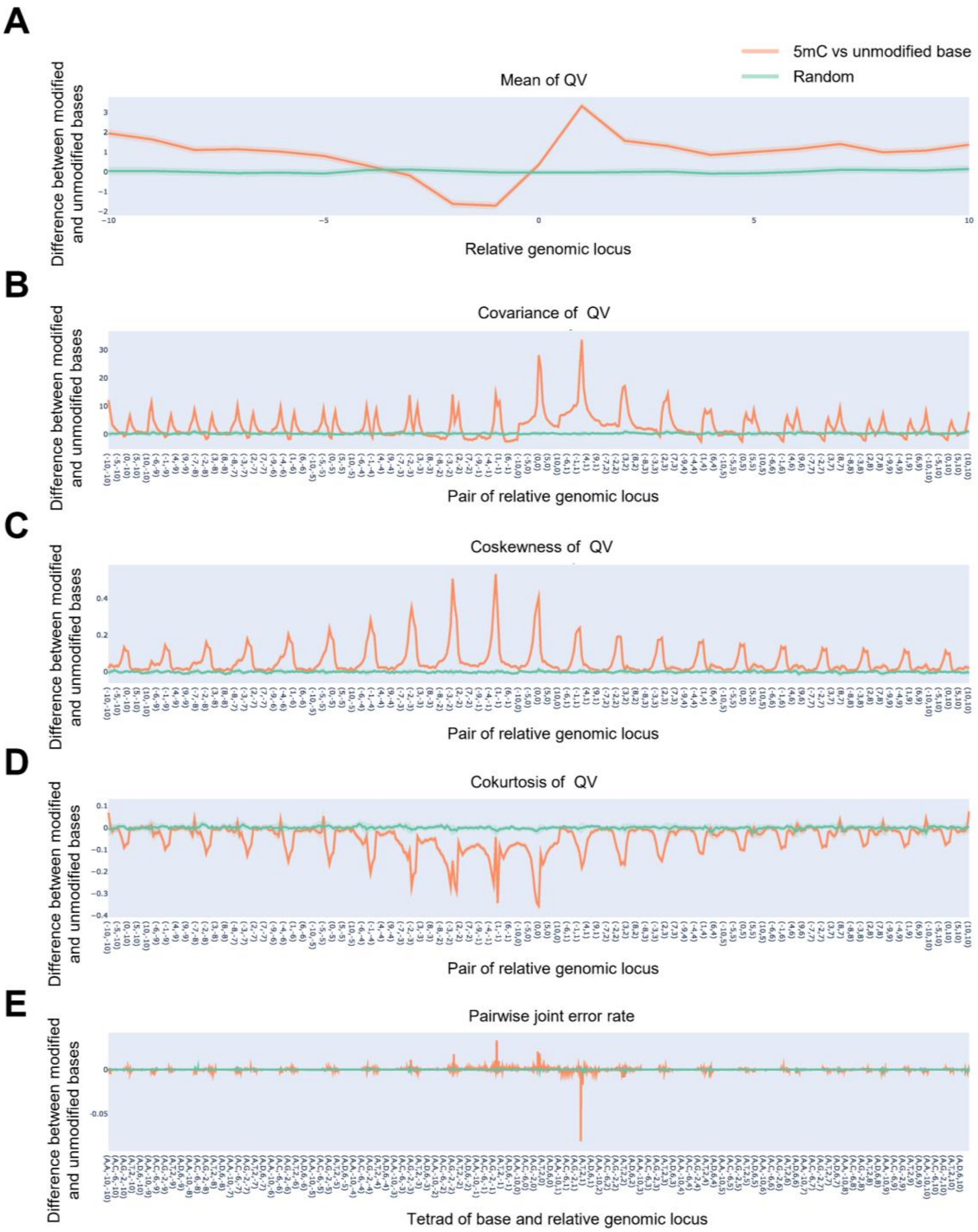

Supplementary Fig. S1. The impact of 5mC on different features. The y-axis shows the differences in the features between the methylated loci and unmethylated loci. **A,** Mean of QV**. B,** Covariance of QV**. C,** Coskewness of QV**. D,** Cokurtosis of QV**. E,** Pairwise joint error rates.

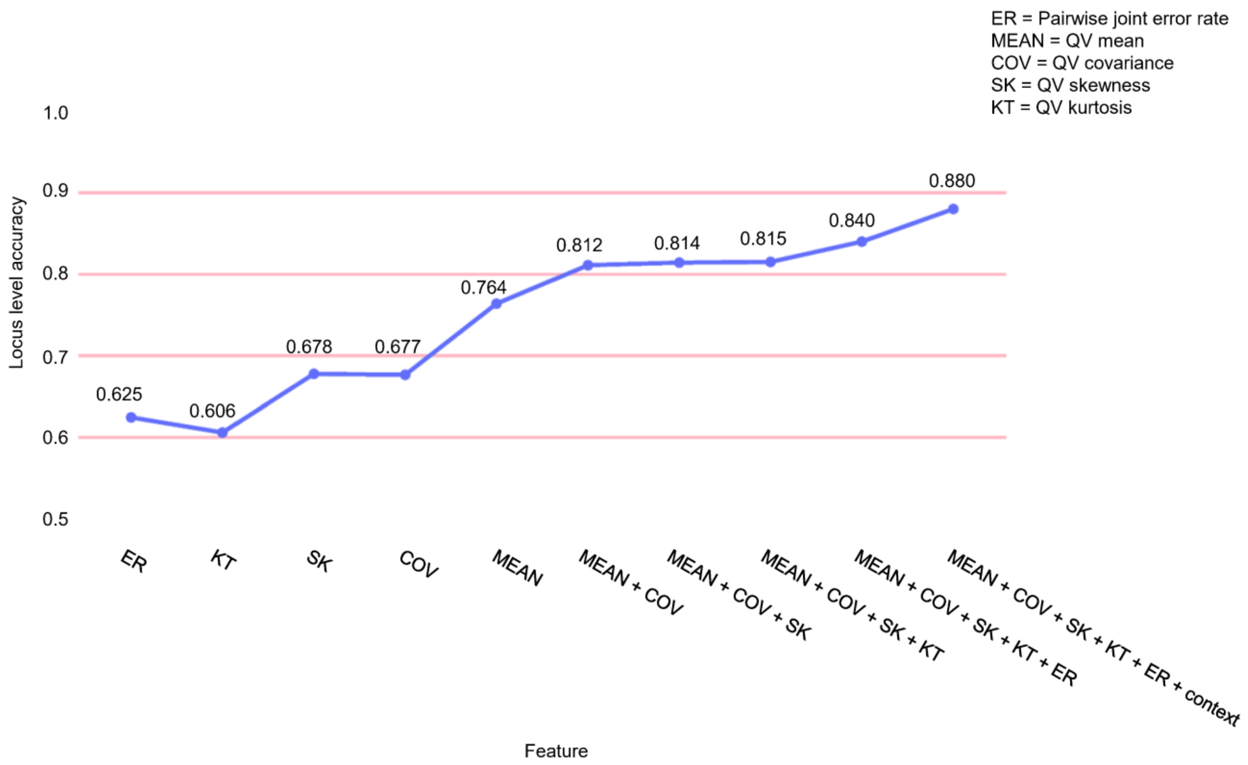

Supplementary Fig. S2. The accuracy of NanoFreeLunch using different features. The accuracy is the Pearson correlation coefficient between the DNA methylation level predicted by NanoFreeLunch and Guppy 6.3.8 on chromosome 6 of the human pangenome data.

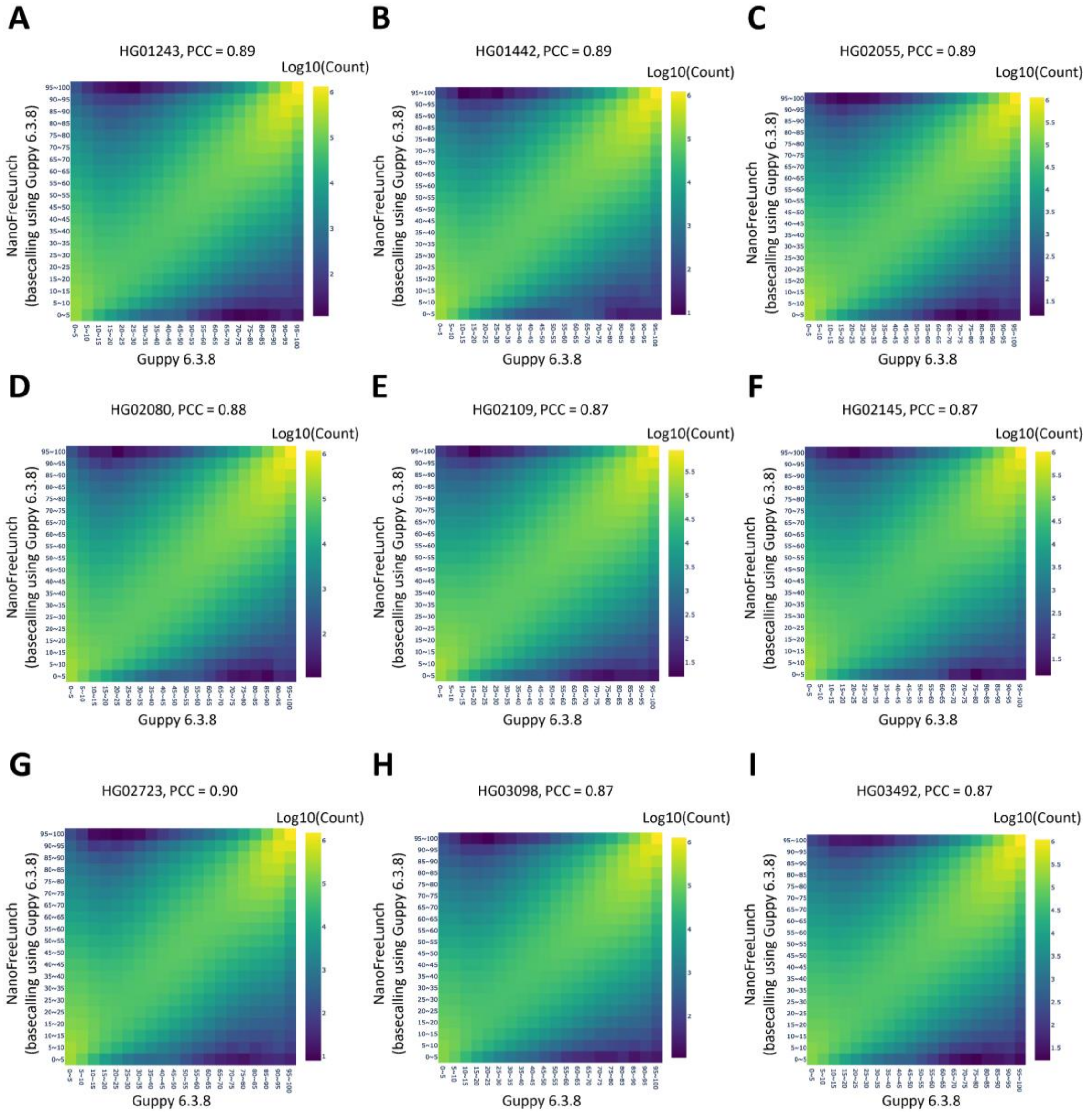

Supplementary Fig. S3. The accuracy of NanoFreeLunch using Guppy 6.3.8 for basecalling on the human pangenome data. The x-axis and y-axis are the DNA methylation levels of each CpG site predicted by Guppy and NanoFreeLunch respectively. Predicted DNA methylation levels are segmented into 20 bins of equal size ranging from 0% to 100%. The color of each bin represents the base-10 logarithm transformation of the number of loci within the bin. PCC denotes Pearson Correlation Coefficient. **A-I,** The results for each sample.

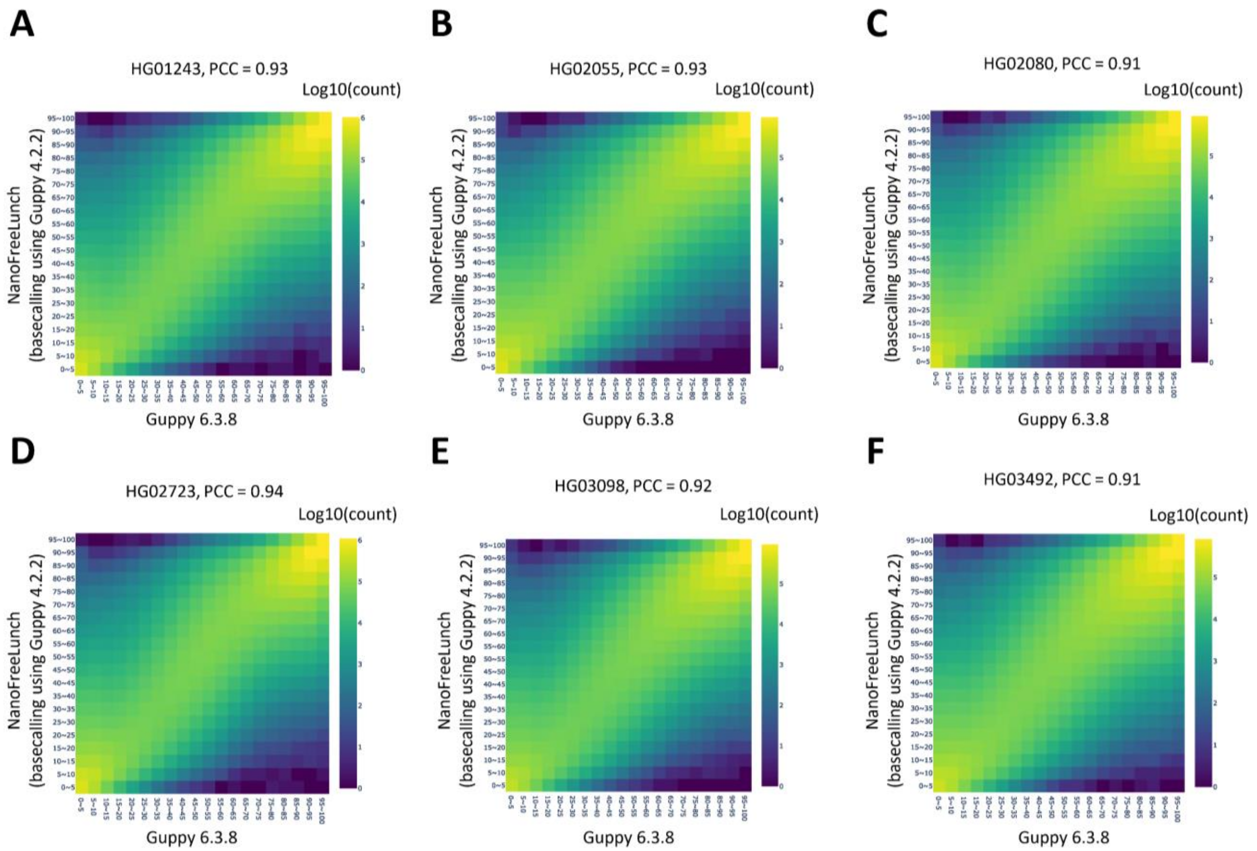

Supplementary Fig. S4. The accuracy of NanoFreeLunch using Guppy 4.2.2 for basecalling on the human pangenome data. The x-axis and y-axis are the DNA methylation levels of each CpG site predicted by Guppy and NanoFreeLunch respectively. Predicted DNA methylation levels are segmented into 20 bins of equal size ranging from 0% to 100%. The color of each bin represents the base-10 logarithm transformation of the number of loci within the bin. PCC denotes Pearson Correlation Coefficient. **A-F,** The results for each sample.

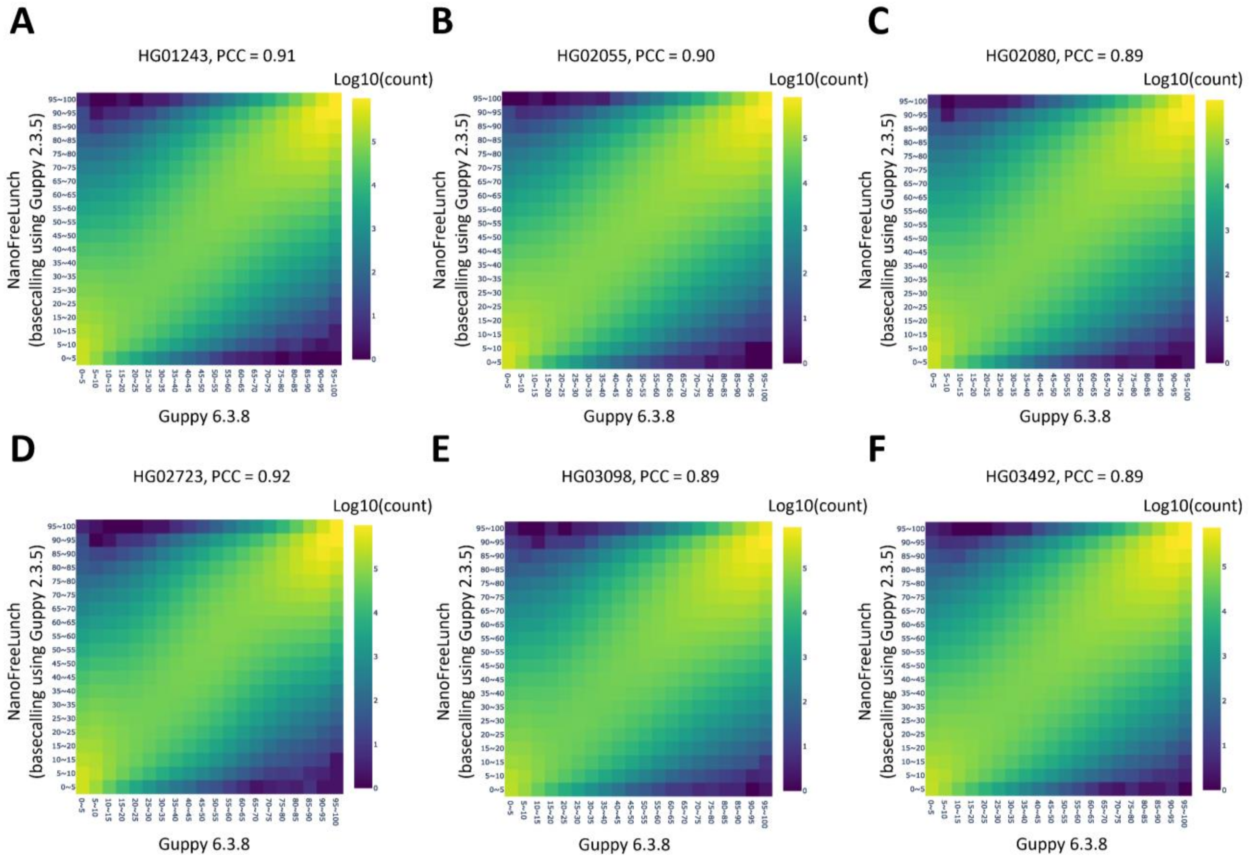

Supplementary Fig. S5. The accuracy of NanoFreeLunch using Guppy 2.3.5 for basecalling on the human pangenome data. The x-axis and y-axis are the DNA methylation levels of each CpG site predicted by Guppy and NanoFreeLunch respectively. Predicted DNA methylation levels are segmented into 20 bins of equal size ranging from 0% to 100%. The color of each bin represents the base-10 logarithm transformation of the number of loci within the bin. PCC denotes Pearson Correlation Coefficient. **A-F,** The results for each sample.

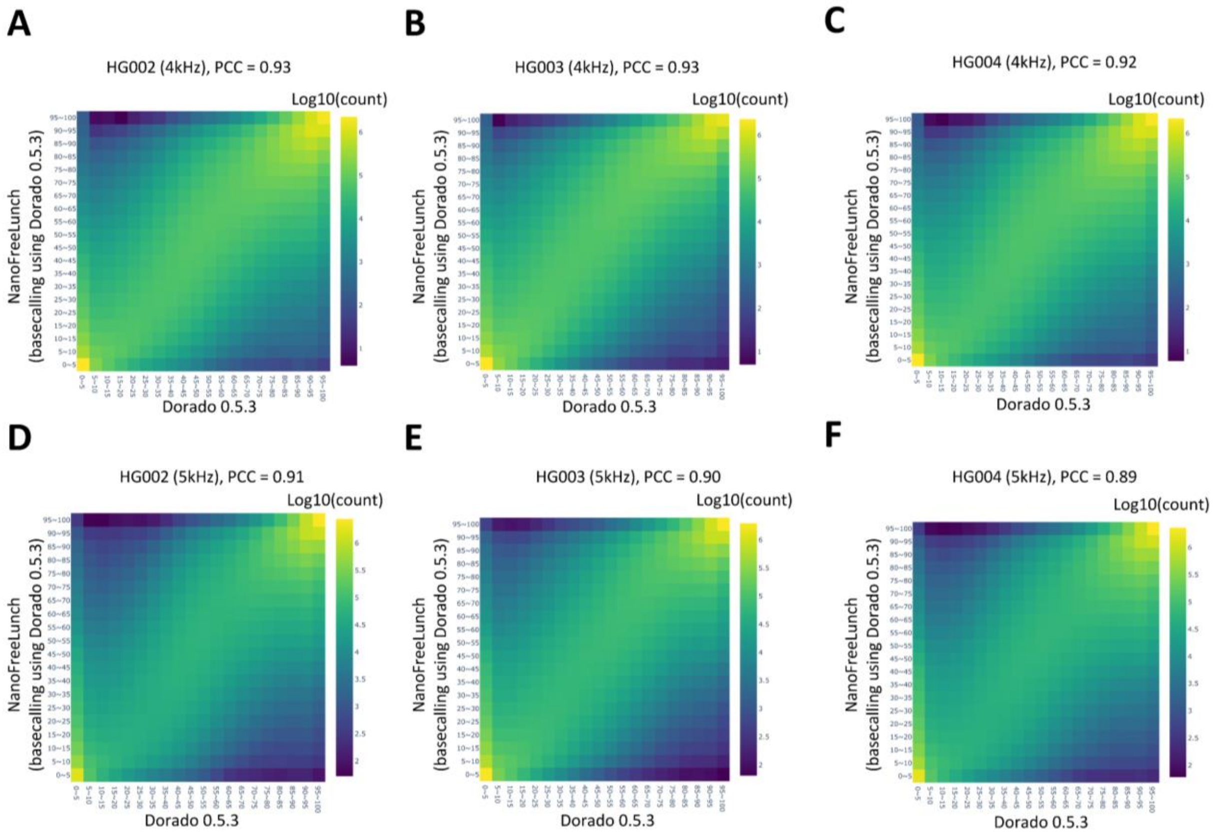

Supplementary Fig. S6. The accuracy of NanoFreeLunch using Dorado 0.5.3 for basecalling on the Ashkenazim Trio data. The x-axis and y-axis are the DNA methylation levels of each CpG site predicted by Dorado and NanoFreeLunch respectively. Predicted DNA methylation levels are segmented into 20 bins of equal size ranging from 0% to 100%. The color of each bin represents the base-10 logarithm transformation of the number of loci within the bin. PCC denotes Pearson Correlation Coefficient. **A-C**, The results for the 4kHz data. **D-F**, The results for the 5kHz data.

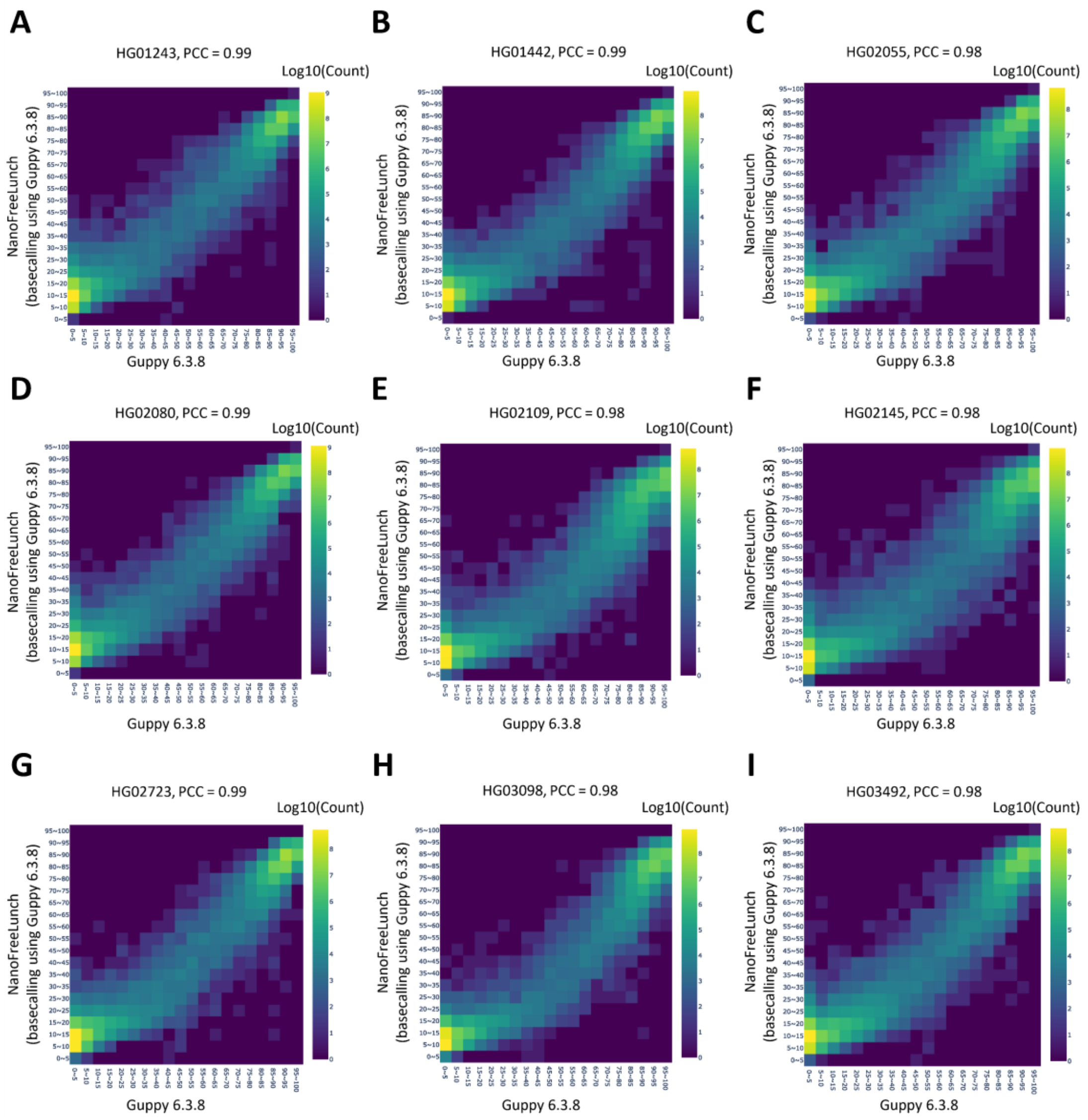

Supplementary Fig. S7. The region-level accuracy of NanoFreeLunch using Guppy 6.3.8 for basecalling on the human pangenome data. The x-axis and y-axis are the average DNA methylation levels of each CpG island predicted by Guppy and NanoFreeLunch respectively. Predicted DNA methylation levels are segmented into 20 bins of equal size ranging from 0% to 100%. The color of each bin represents the base-10 logarithm transformation of the number of CpG islands within the bin. PCC denotes Pearson Correlation Coefficient. **A-I,** The results for each sample.

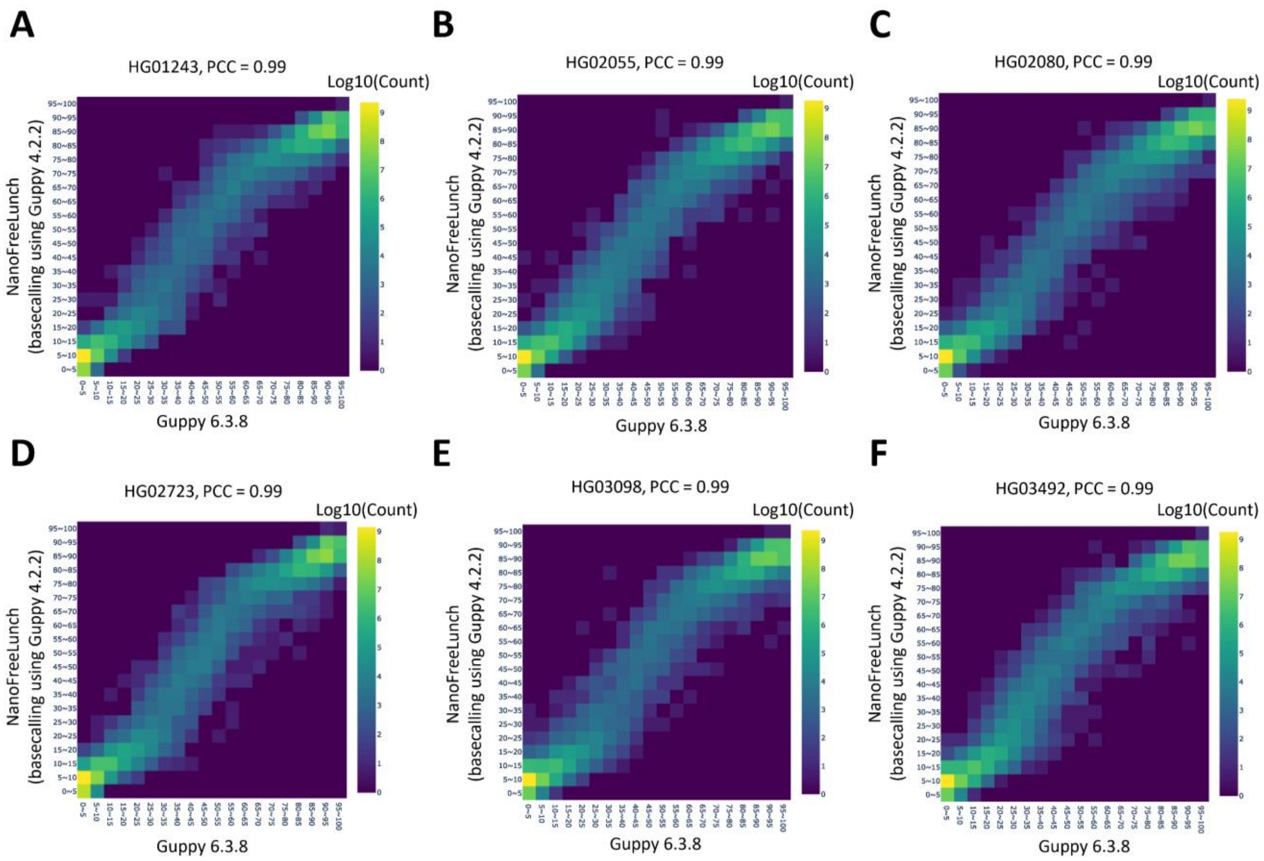

Supplementary Fig. S8. The region-level accuracy of NanoFreeLunch using Guppy 4.2.2 for basecalling on the human pangenome data. The x-axis and y-axis are the average DNA methylation levels of each CpG island predicted by Guppy and NanoFreeLunch respectively. Predicted DNA methylation levels are segmented into 20 bins of equal size ranging from 0% to 100%. The color of each bin represents the base-10 logarithm transformation of the number of CpG islands within the bin. PCC denotes Pearson Correlation Coefficient. **A-F,** The results for each sample.

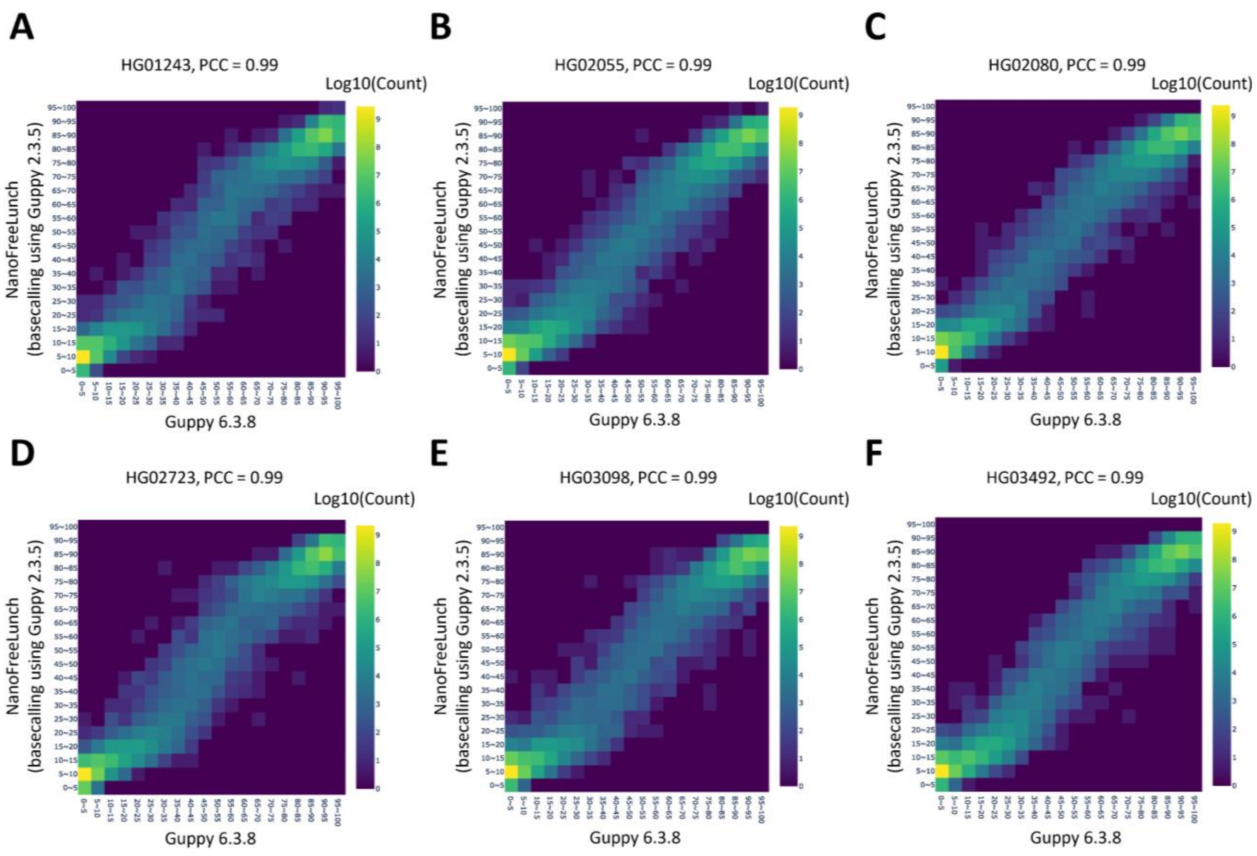

Supplementary Fig. S9. The region-level accuracy of NanoFreeLunch using Guppy 2.3.5 for basecalling on the human pangenome data. The x-axis and y-axis are the average DNA methylation levels of each CpG island predicted by Guppy and NanoFreeLunch respectively. Predicted DNA methylation levels are segmented into 20 bins of equal size ranging from 0% to 100%. The color of each bin represents the base-10 logarithm transformation of the number of CpG islands within the bin. PCC denotes Pearson Correlation Coefficient. **A-F,** The results for each sample.

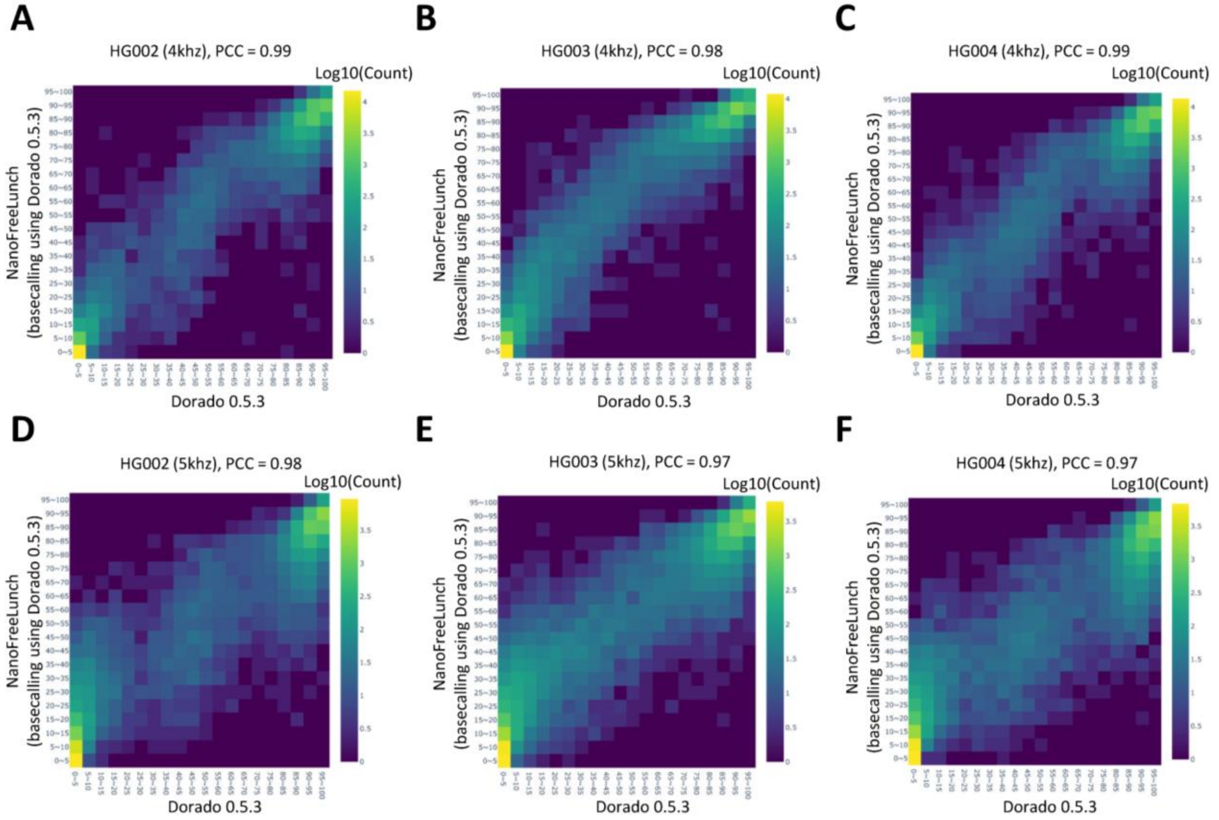

Supplementary Fig. S10. The region-level accuracy of NanoFreeLunch using Dorado 0.5.3 for basecalling on the Ashkenazim Trio data. The x-axis and y-axis are the average DNA methylation levels of each CpG island predicted by Guppy and NanoFreeLunch respectively. Predicted DNA methylation levels are segmented into 20 bins of equal size ranging from 0% to 100%. The color of each bin represents the base-10 logarithm transformation of the number of CpG islands within the bin. PCC denotes Pearson Correlation Coefficient. **A-C**, The results for the 4kHz data. **D-F**, The results for the 5kHz data.

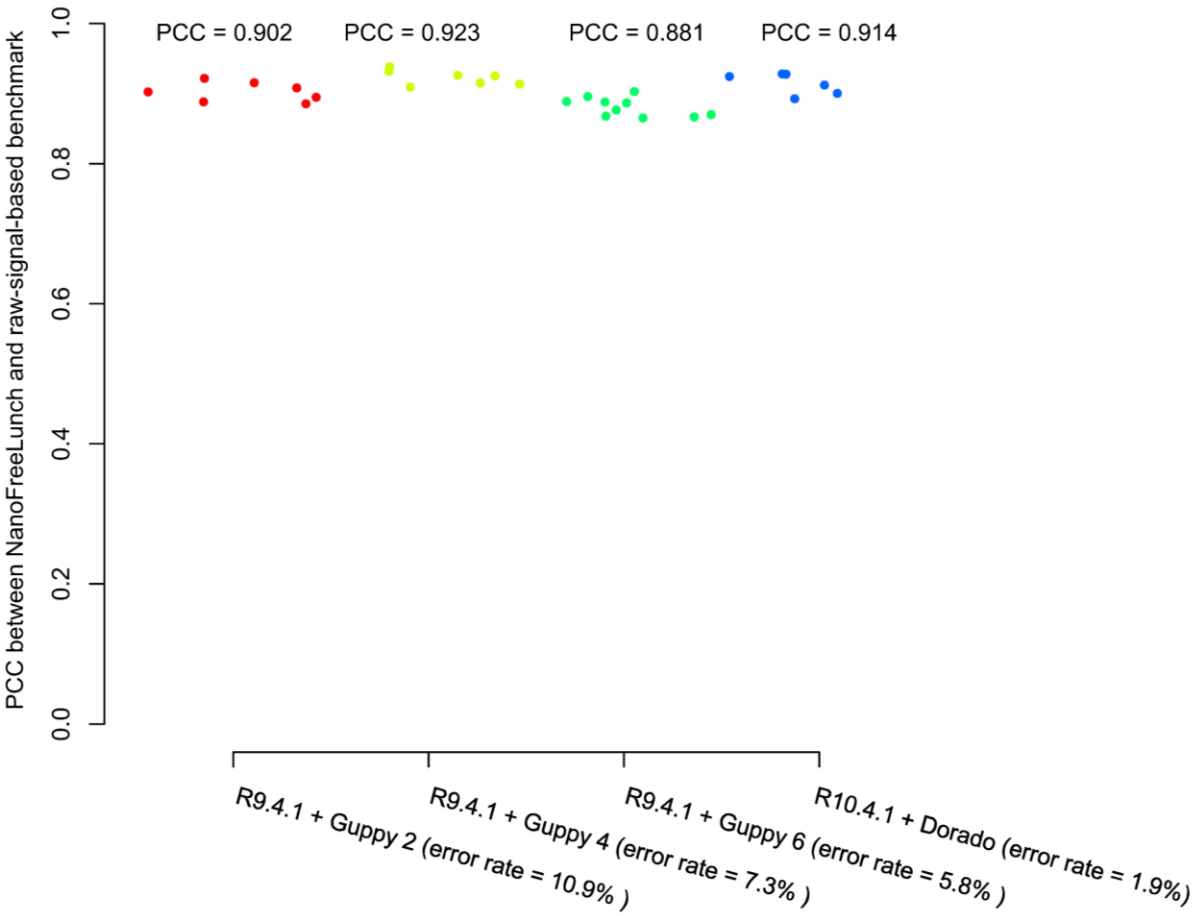

Supplementary Fig. S11. The accuracy of NanoFreeLunch using the data obtained by different flowcell types and basecallers. PCC represents Pearson Correlation Coefficient. The PCC in the figure is the average PCC of each category.

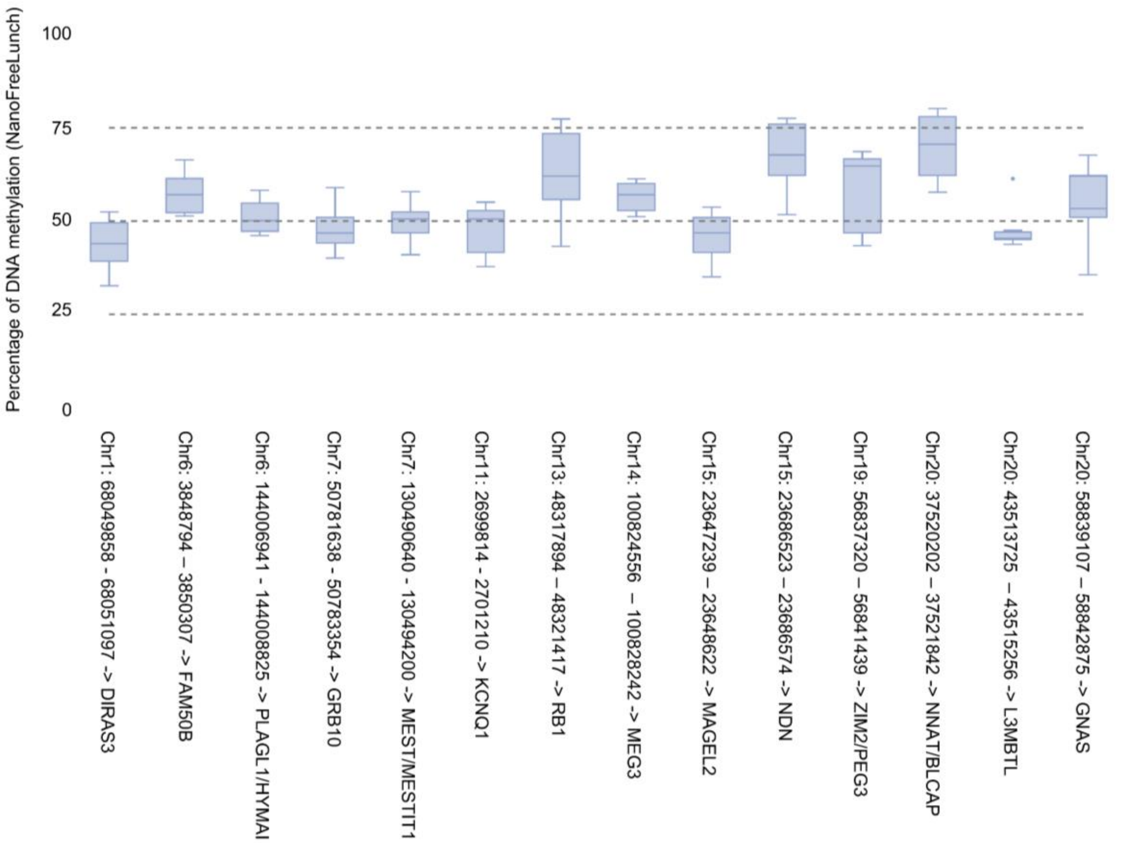

Supplementary Fig. S12. The average DNA methylation level of ICR predicted by NanoFreeLunch using Guppy 2.3.5 for basecalling. Each boxplot illustrates the distribution of the average DNA methylation levels of ICRs predicted by NanoFreeLunch using human pangenome data. The line in each box represents the median. The lower and upper bounds of the box correspond to the first (Q1) and third (Q3) quartiles, respectively. The lower fence is determined as the last sample point below 1.5 times the interquartile range (IQR), calculated as Q3 minus Q1. Similarly, the upper fence is identified as the last sample point above 1.5 times the IQR. In the x-axis, the region on the left of "->" is the genomic region of the ICR on GRCh38, and the gene symbol on the right is the putative gene associated with the ICR. The basecalling results of Guppy 2.3.5 are used as the input of NanoFreeLunch.

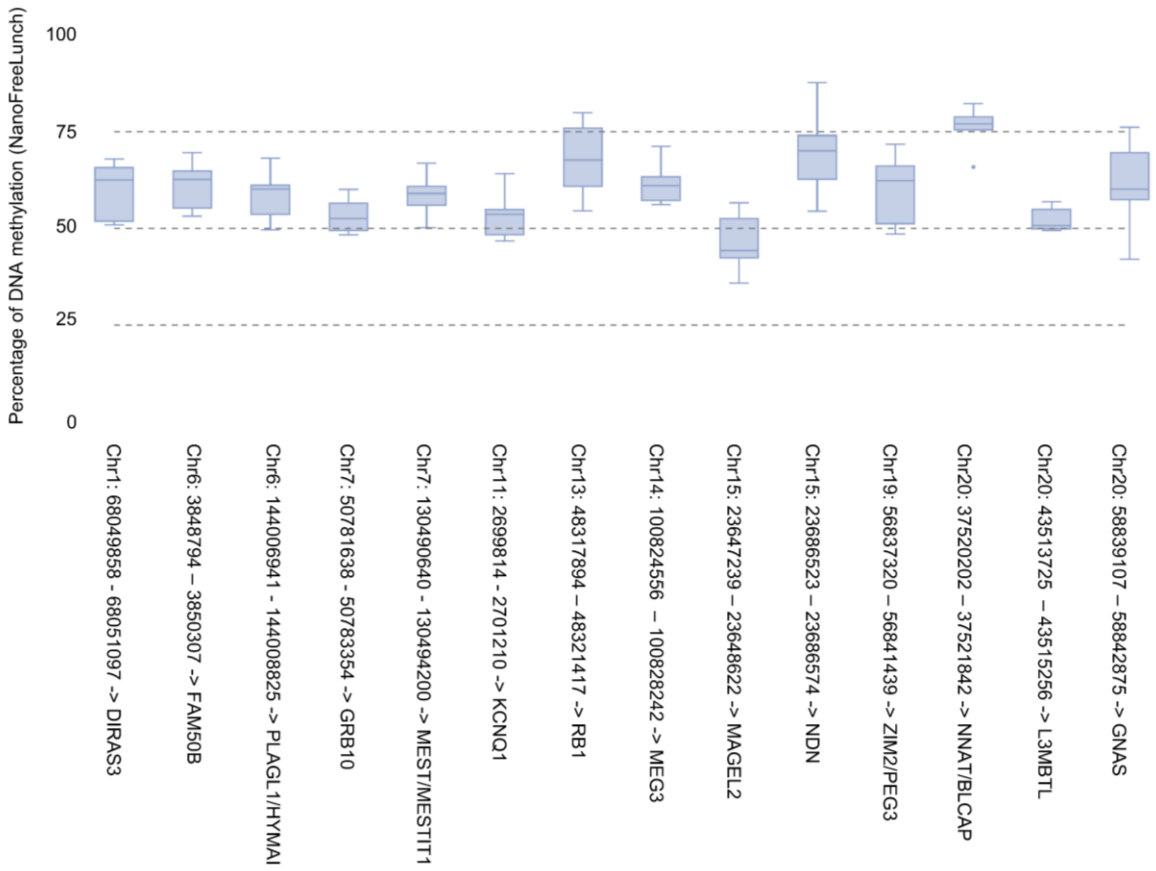

Supplementary Fig. S13. The average DNA methylation level of ICR predicted by NanoFreeLunch using Guppy 4.2.2 for basecalling. Each boxplot illustrates the distribution of the average DNA methylation levels of ICRs predicted by NanoFreeLunch using human pangenome data. The line in each box represents the median. The lower and upper bounds of the box correspond to the first (Q1) and third (Q3) quartiles, respectively. The lower fence is determined as the last sample point below 1.5 times the interquartile range (IQR), calculated as Q3 minus Q1. Similarly, the upper fence is identified as the last sample point above 1.5 times the IQR. In the x-axis, the region on the left of "->" is the genomic region of the ICR on GRCh38, and the gene symbol on the right is the putative gene associated with the ICR. The basecalling results of Guppy 4.2.2 are used as the input of NanoFreeLunch.

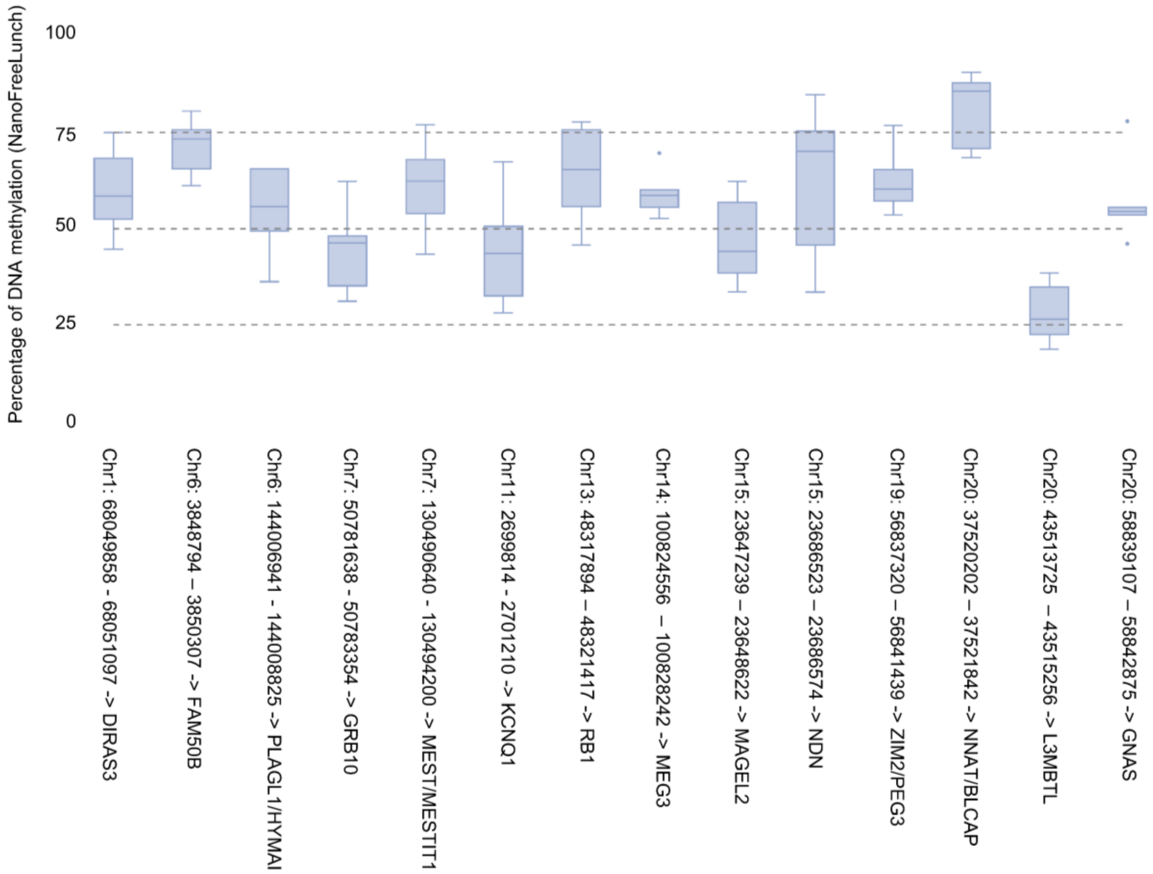

Supplementary Fig. S14. The average DNA methylation level of ICR predicted by NanoFreeLunch using Dorado 0.5.3 for basecalling. Each boxplot illustrates the distribution of the average DNA methylation levels of ICRs predicted by NanoFreeLunch using the R10 Ashkenazim trio data. The line in each box represents the median. The lower and upper bounds of the box correspond to the first (Q1) and third (Q3) quartiles, respectively. The lower fence is determined as the last sample point below 1.5 times the interquartile range (IQR), calculated as Q3 minus Q1. Similarly, the upper fence is identified as the last sample point above 1.5 times the IQR. In the x-axis, the region on the left of "->" is the genomic region of the ICR on GRCh38, and the gene symbol on the right is the putative gene associated with the ICR. The basecalling results of Dorado 0.5.3 are used as the input of NanoFreeLunch.

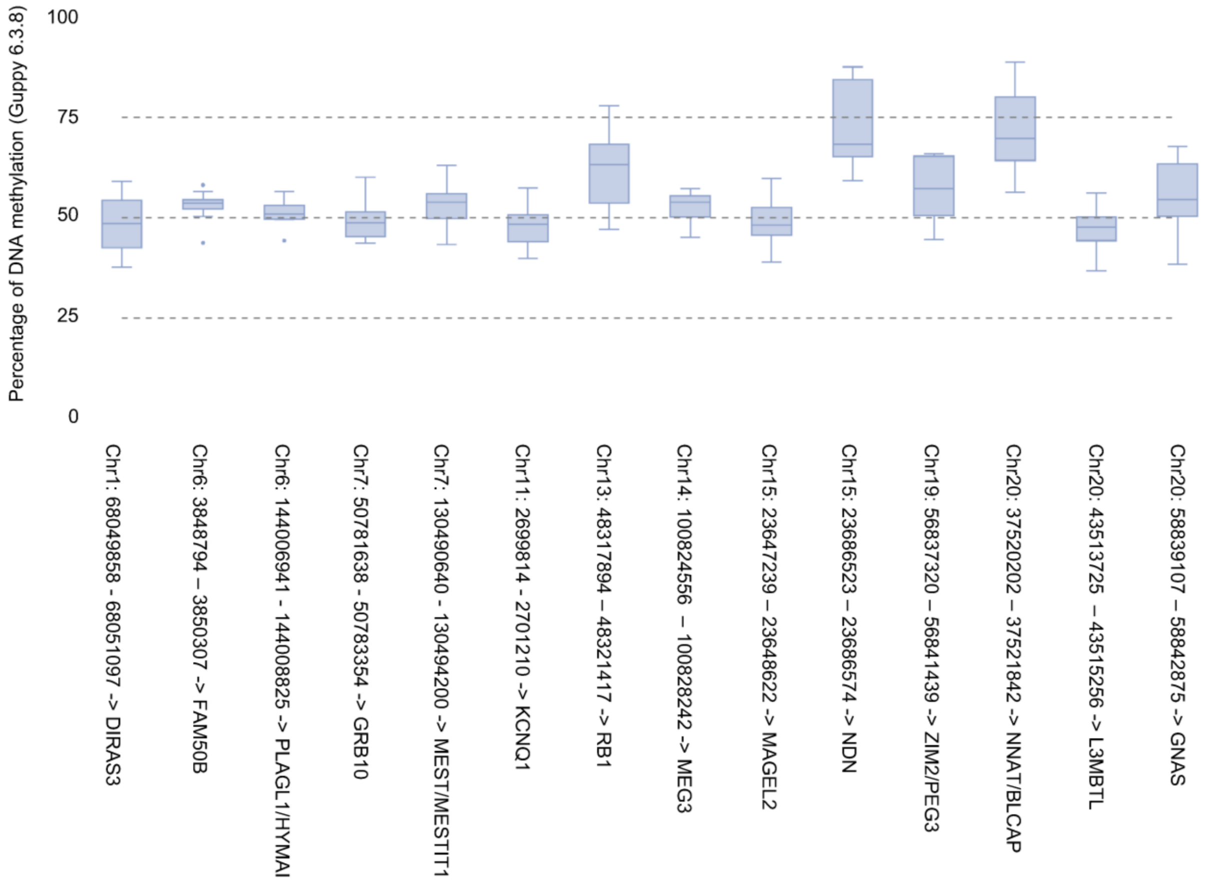

Supplementary Fig. S15. The average DNA methylation level of ICR predicted by Guppy 6.3.8. Each boxplot illustrates the distribution of the average DNA methylation levels of ICRs predicted by Guppy using human pangenome data. The line in each box represents the median. The lower and upper bounds of the box correspond to the first (Q1) and third (Q3) quartiles, respectively. The lower fence is determined as the last sample point below 1.5 times the interquartile range (IQR), calculated as Q3 minus Q1. Similarly, the upper fence is identified as the last sample point above 1.5 times the IQR. In the x-axis, the region on the left of “->” is the genomic region of the ICR on GRCh38, and the gene symbol on the right is the putative gene associated with the ICR.

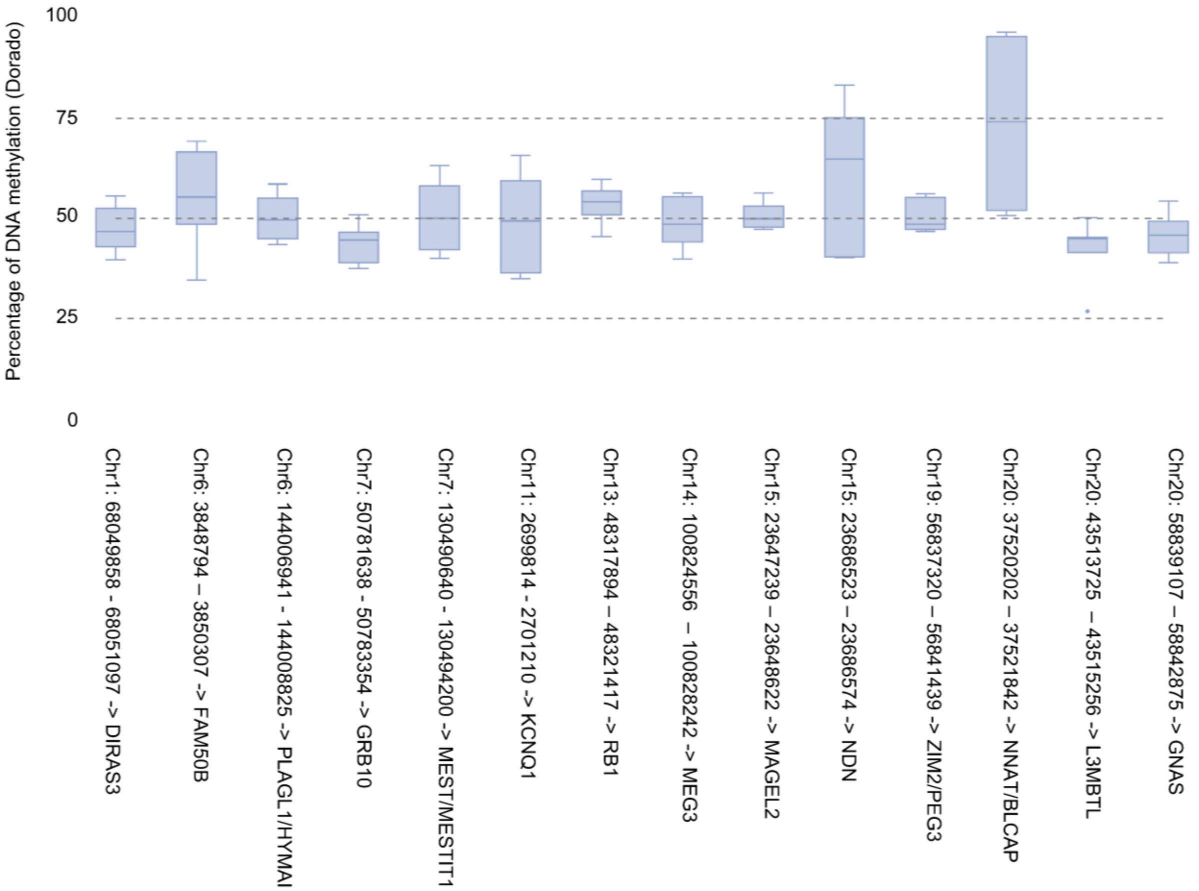

Supplementary Fig. S16. The average DNA methylation level of ICR predicted by Dorado 0.5.3. Each boxplot illustrates the distribution of the average DNA methylation levels of ICRs predicted by Dorado using the R10 Ashkenazim trio data. The line in each box represents the median. The lower and upper bounds of the box correspond to the first (Q1) and third (Q3) quartiles, respectively. The lower fence is determined as the last sample point below 1.5 times the interquartile range (IQR), calculated as Q3 minus Q1. Similarly, the upper fence is identified as the last sample point above 1.5 times the IQR. In the x-axis, the region on the left of "->" is the genomic region of the ICR on GRCh38, and the gene symbol on the right is the putative gene associated with the ICR.

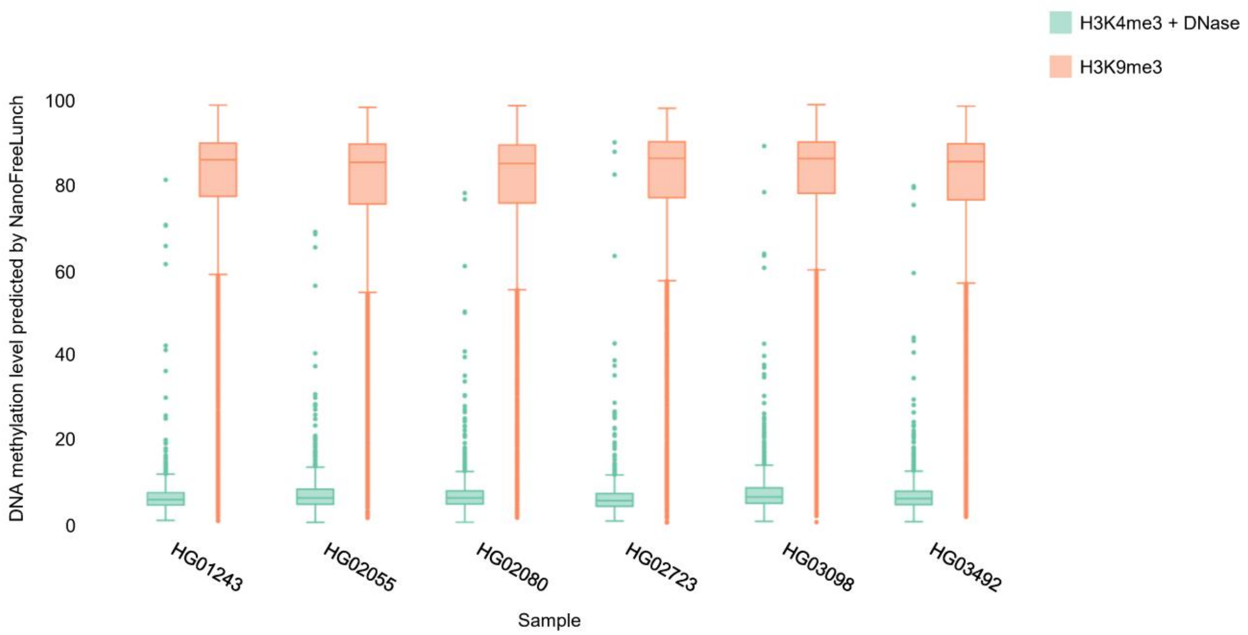

Supplementary Fig. S17. Comparing DNA methylation level predicted by NanoFreeLunch using Guppy 4.2.2 for basecalling with other epigenetic markers. Each boxplot depicts the distribution of average DNA methylation levels in H3K9me3 regions or DNase hypersensitive regions marked by H3K4me3, predicted by NanoFreeLunch using human pangenome data. Different colors represent distinct regions. The line in each box represents the median. The lower and upper bounds of the box correspond to the first (Q1) and third (Q3) quartiles, respectively. The lower fence is determined as the last sample point below 1.5 times the interquartile range (IQR), calculated as Q3 minus Q1. Similarly, the upper fence is identified as the last sample point above 1.5 times the IQR. The histone modification data and DNase data are obtained from the GM12878 cell line of ENCODE. The basecalling results of Guppy 4.2.2 are used as the input of NanoFreeLunch.

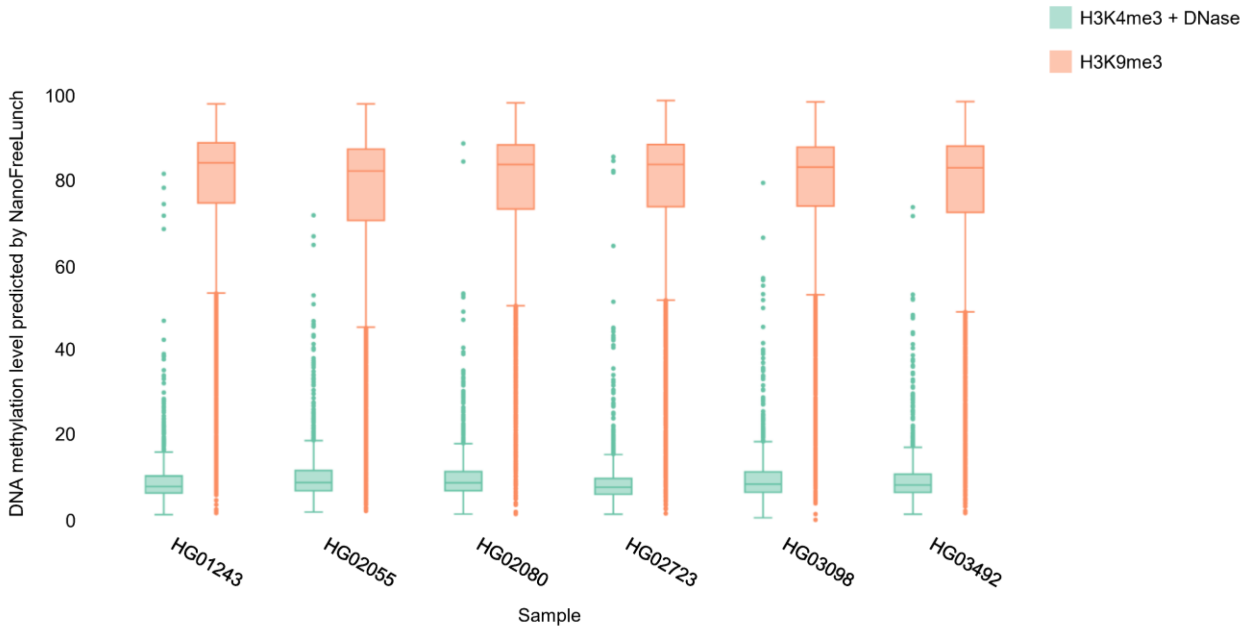

Supplementary Fig. S18. Comparing DNA methylation level predicted by NanoFreeLunch using Guppy 2.3.5 for basecalling with other epigenetic markers. Each boxplot depicts the distribution of average DNA methylation levels in H3K9me3 regions or DNase hypersensitive regions marked by H3K4me3, predicted by NanoFreeLunch using human pangenome data. Different colors represent distinct regions. The line in each box represents the median. The lower and upper bounds of the box correspond to the first (Q1) and third (Q3) quartiles, respectively. The lower fence is determined as the last sample point below 1.5 times the interquartile range (IQR), calculated as Q3 minus Q1. Similarly, the upper fence is identified as the last sample point above 1.5 times the IQR. The histone modification data and DNase data are obtained from the GM12878 cell line of ENCODE. The basecalling results of Guppy 2.3.5 are used as the input of NanoFreeLunch.

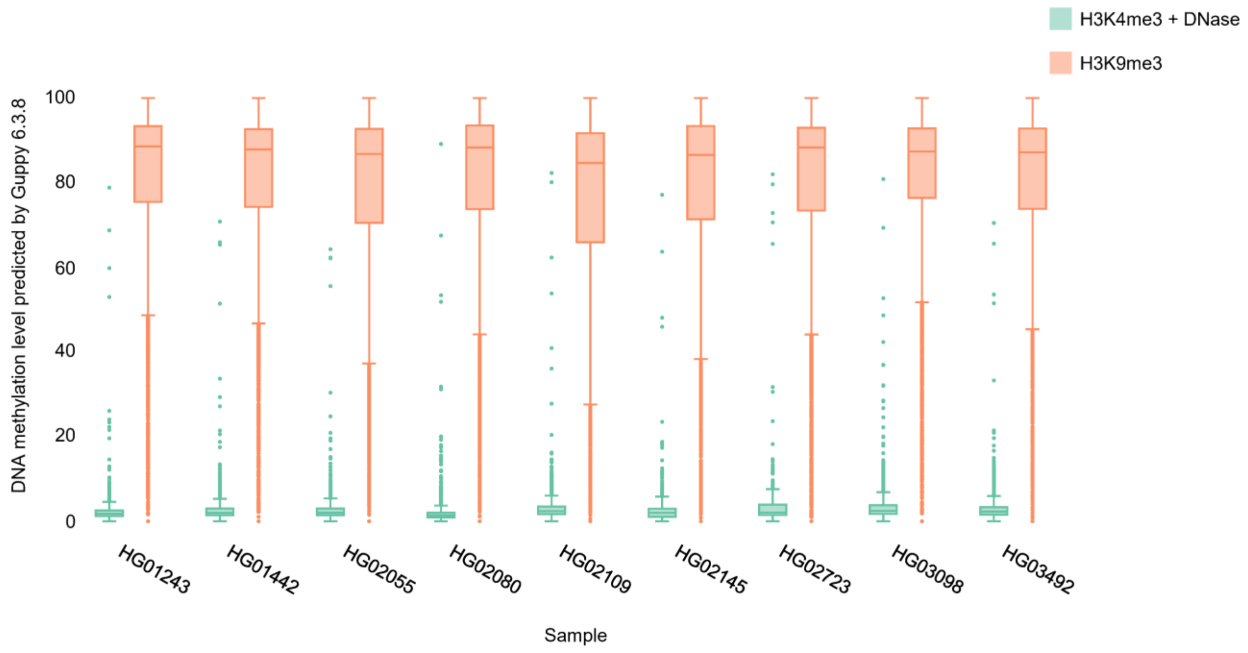

Supplementary Fig. S19. The average DNA methylation level of regions with different epigenetic markers predicted by Guppy 6.3.8. Each boxplot depicts the distribution of average DNA methylation levels in H3K9me3 regions or DNase hypersensitive regions marked by H3K4me3, predicted by Guppy using human pangenome data. Different colors represent distinct regions. The line in each box represents the median. The lower and upper bounds of the box correspond to the first (Q1) and third (Q3) quartiles, respectively. The lower fence is determined as the last sample point below 1.5 times the interquartile range (IQR), calculated as Q3 minus Q1. Similarly, the upper fence is identified as the last sample point above 1.5 times the IQR. The histone modification data and DNase data are obtained from the GM12878 cell line of ENCODE.

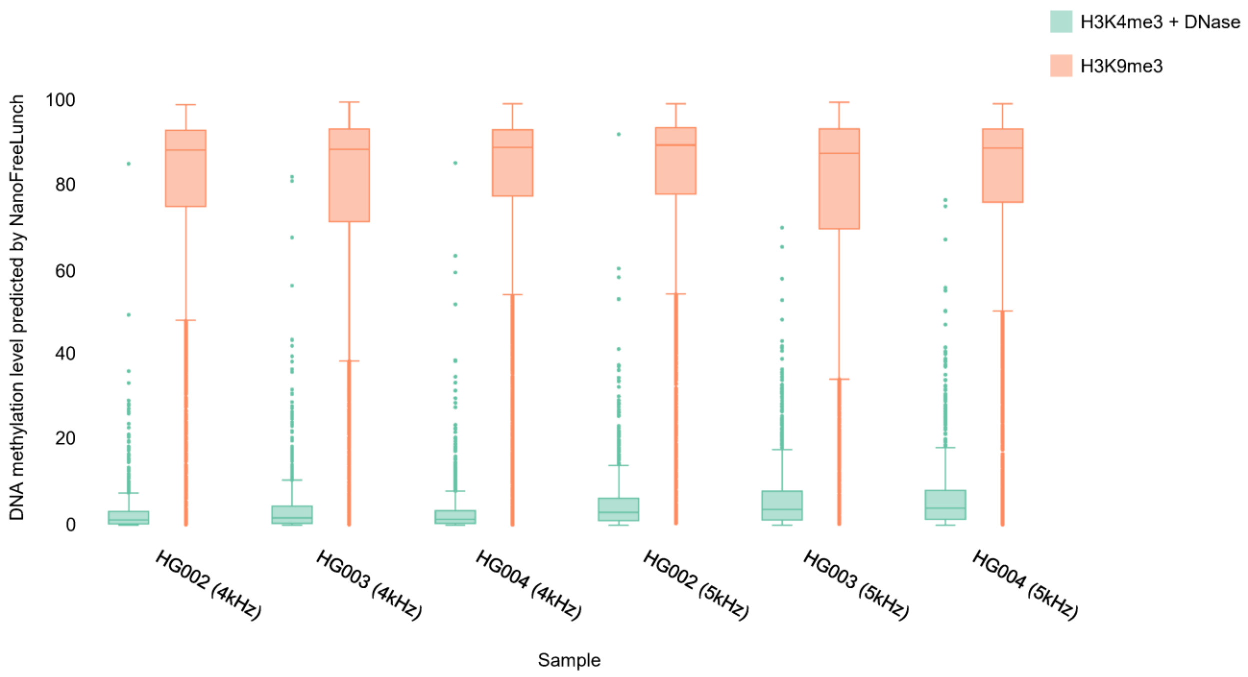

Supplementary Fig. S20. Comparing DNA methylation level predicted by NanoFreeLunch using Dorado 0.5.3 for basecalling with other epigenetic markers. Each boxplot depicts the distribution of average DNA methylation levels in H3K9me3 regions or DNase hypersensitive regions marked by H3K4me3, predicted by NanoFreeLunch using the R10 Ashkenazim trio data. Different colors represent distinct regions. The line in each box represents the median. The lower and upper bounds of the box correspond to the first (Q1) and third (Q3) quartiles, respectively. The lower fence is determined as the last sample point below 1.5 times the interquartile range (IQR), calculated as Q3 minus Q1. Similarly, the upper fence is identified as the last sample point above 1.5 times the IQR. The histone modification data and DNase data are obtained from the GM12878 cell line of ENCODE. The basecalling results of Dorado 0.5.3 are used as the input of NanoFreeLunch.

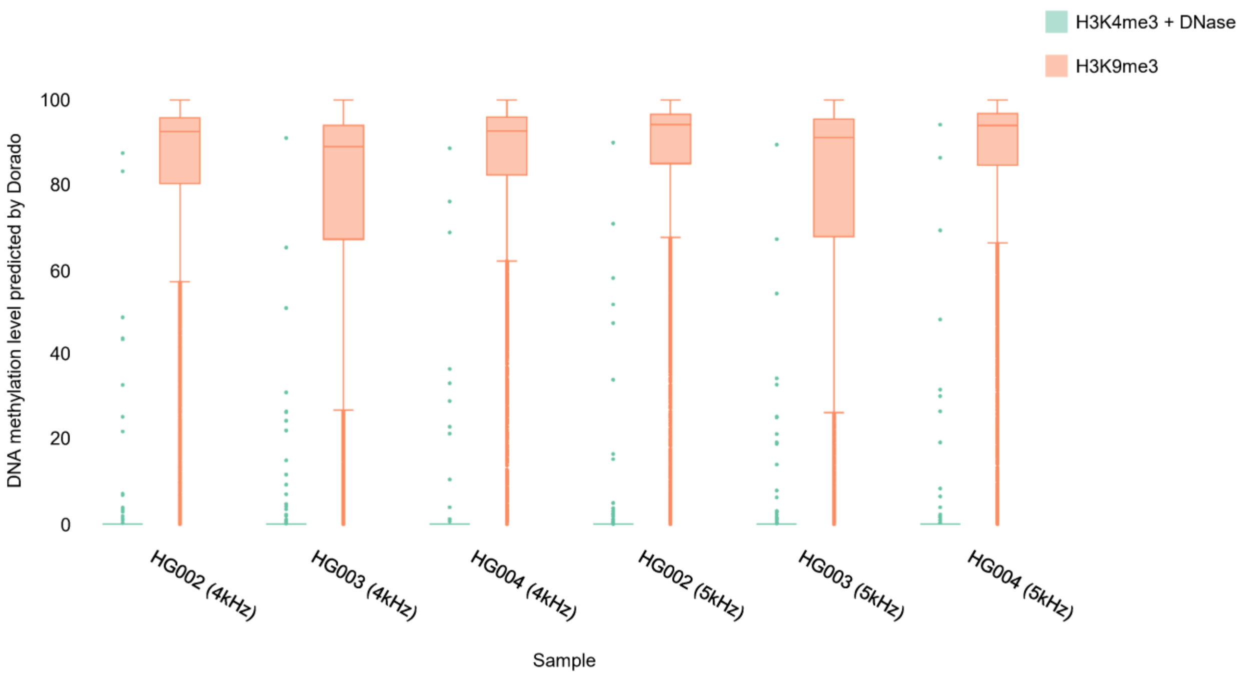

Supplementary Fig. S21. The average DNA methylation level of regions with different epigenetic markers predicted by Dorado 0.5.3. Each boxplot depicts the distribution of average DNA methylation levels in H3K9me3 regions or DNase hypersensitive regions marked by H3K4me3, predicted by Dorado using the R10 Ashkenazim trio data. Different colors represent distinct regions. The line in each box represents the median. The lower and upper bounds of the box correspond to the first (Q1) and third (Q3) quartiles, respectively. The lower fence is determined as the last sample point below 1.5 times the interquartile range (IQR), calculated as Q3 minus Q1. Similarly, the upper fence is identified as the last sample point above 1.5 times the IQR. The histone modification data and DNase data are obtained from the GM12878 cell line of ENCODE.

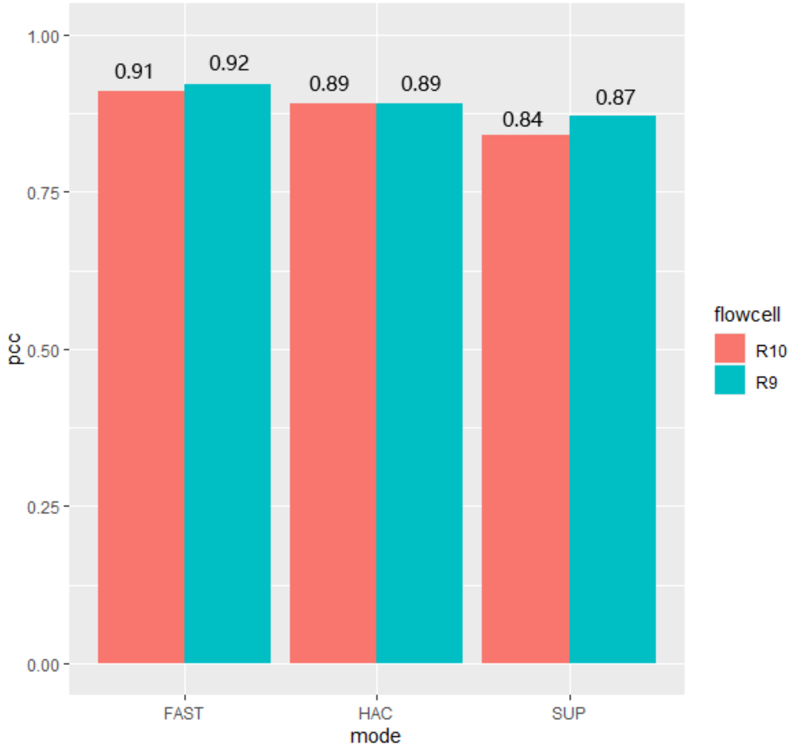

Supplementary Fig. 22. The impact of basecalling mode on the accuracy of NanoFreeLunch. Comparison of NanoFreeLunch performance using R9 (Guppy) and R10 (Dorado) data basecalled with FAST, HAC, and SUP modes. Bars show the Pearson correlation coefficient (PCC) between methylation levels predicted by NanoFreeLunch and those predicted by the respective basecaller.

Cover letter Click here to access/download;Personal Cover;coverletter-v7.docx

Zhixing Feng, Ph.D.
Assistant Professor
Department of Clinical Genetics
Xinhua Hospital Affiliated to Shanghai Jiao Tong University School of Medicine
Shanghai, China

Dear Dr Hongling Zhou,

We are pleased to submit our revised manuscript titled "**Quantitative Detection of DNA Methylation from Nanopore Sequencing Data without Raw Signals**". We would be grateful if you could consider it for formal publication as a *Technical Note* in GigaScience.

We have carefully addressed all comments raised by the reviewers and revised the manuscript accordingly. Details of our responses are provided in the rebuttal letter. Additionally, we have added the BioTools ID and RRID to the Code Availability section, as requested.

Thank you for considering our work.

Sincerely,

Zhixing Feng

Kongjiang Road 1655, Shanghai, 200092, China
Email: fengzhixing@shsmu.edu.cn

response to reviewers comments Click here to access/download;Personal Cover;response-to-reviewers.docx

We sincerely thank the reviewers for their constructive feedback. The comments are very helpful in improving the quality of this manuscript. Below, we provide a detailed point-by-point response to the reviewers' comments, and we highlight the changes in the revised manuscript. The page and line numbers cited in the responses below refer to the revised manuscript.

Reviewer reports:

Reviewer #1: In the manuscript "Quantitative Detection of DNA Methylation from Nanopore Sequencing Data without Raw Signals", the authors present a novel method to infer DNA methylation from sequence data without the need for the original signal data. This work describes an exciting new tool to utilize existing ONT data for epigenomics without the need for the massive POD5/FAST5 files.

We sincerely thank the reviewer for highlighting the significance of our study. We also appreciate the constructive comments and have addressed them point-by-point below.

Major comments

One of the major claims of this work is that it can remove the need to store POD5/FAST5 files which is expensive due to their size. However, they do not offer any indication of resource requirements for model training or training duration. This is particularly relevant to researchers who would like to leverage this tool but work on species other than human as they would likely need to train their own species specific model. Moreover, for other species, there is often far less data available to train such models. Can the authors offer a recommendation of the minimum training data requirements?

We agree with the reviewer that sample size for model training is important. To address this issue, we performed saturation analysis by downsampling the human training data (chr10 of HG01109) to 1%, 10%, 20%, 50%, 75%, and 90% respectively. The results (Fig. R1) show that accuracy plateaus at approximately 883,912 CpG sites (highlighted in red). While increasing sample size beyond this point yields no further gains, training with 117,855 CpG sites achieves an accuracy of 0.85 (PCC between NanoFreeLunch and Guppy), which represents 96% of the peak accuracy (PCC = 0.89) achieved with the full dataset (tested on chr6 of HG01243). This suggests that using roughly 118,000

CpG sites offers a favorable balance between data requirements and performance for the human data analyzed. While it is difficult to define an exact universal minimum training data requirement applicable to all species, these results provide valuable insights into the relationship between training sample size and accuracy. Researchers working on other species can adapt this saturation analysis to their own datasets to estimate suitable training sizes given their specific performance goals and data availability.

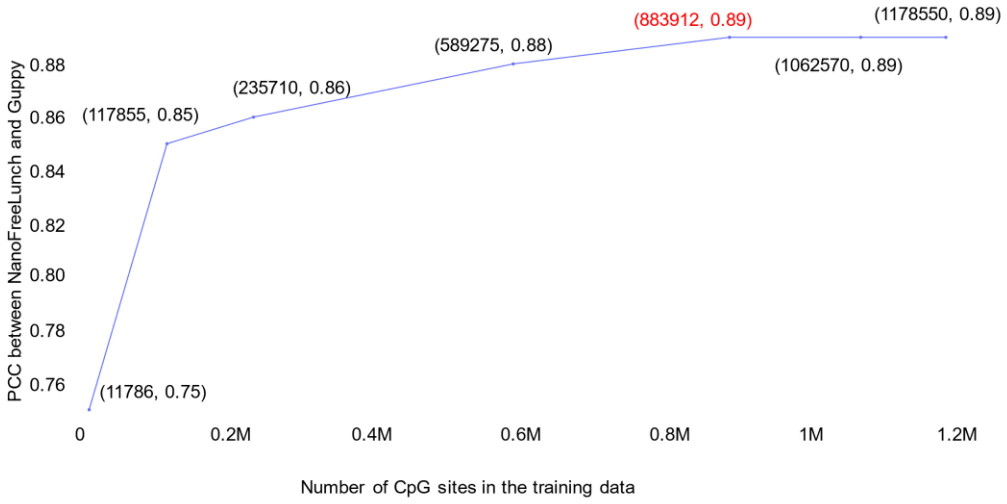

Fig. R1. Impact of training sample size on the accuracy of NanoFreeLunch

Line 289: The authors used the HAC option for basecalling. Did the authors test with different base calling models, I.e., SUP or FAST? Can the authors offer some discussion as to why they picked this model and whether or not they tested the other models? If they tested the other models was there a difference in the correlations?

Yes, we tested basecalling with FAST, HAC, and SUP modes. We found that the choice of basecalling mode has a minor, but measurable, impact on NanoFreeLunch's accuracy. The results show that FAST mode yielding slightly higher accuracy than HAC mode, and SUP mode yielding slightly lower accuracy for both R9 and R10 data (Fig. R2). In the previous version of the manuscript, we used HAC mode because it is commonly used and the default recommended mode in MinKNOW, the official sequencer software that wraps Guppy/Dorado for basecalling (https://nanoporetech.com/document/experiment-companion-minknow#starting-a-sequencing-run-on-promethion-p2i).

The exact reason for this discrepancy between basecalling modes is non-trivial to determine. Our results suggest that the relationship between basecalling accuracy and NanoFreeLunch accuracy is complex and non-monotonic. For instance, while R10 data has significantly higher basecalling accuracy than R9 data, NanoFreeLunch accuracy is similar for both flowcell types (Fig. R2). This indicates that the specific types of error/QV pattern introduced by different basecalling modes (FAST, HAC, SUP) may have complicated and hard-to-interpret effects on the underlying features used by NanoFreeLunch to predict methylation.

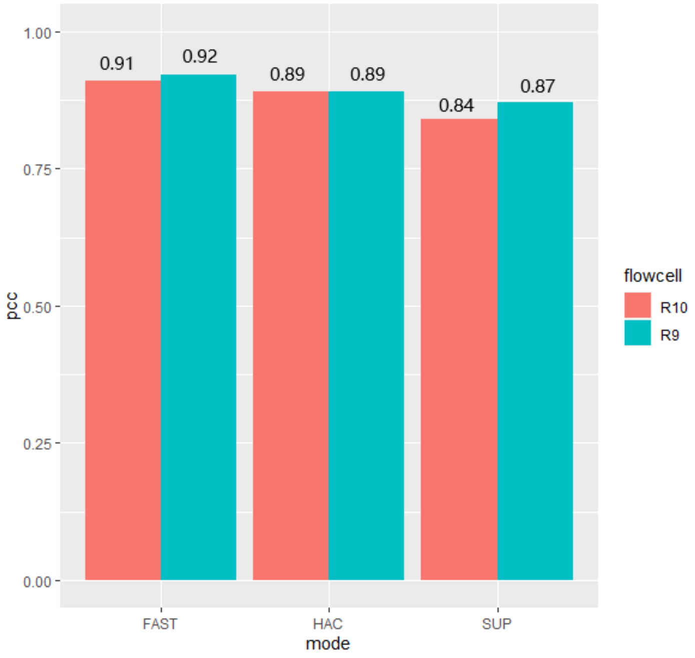

Fig. R2. PCC between NanoFreeLunch and Guppy/Dorado on chromosome 6 of human data.

We added a paragraph in Discussion (page 22, line 241-255) as following and added Fig.R2 as Supplementary Fig. S22 to revised manuscript.

"*In this study, we used HAC mode for basecalling, which is commonly used and recommended mode since it has a good balance between speed and accuracy. However, basecalling mode might affect methylation detection of NanoFreeLunch since different modes might have different error and QV patterns. Using models trained on chromosome 10 (HG01109 for R9, HG002 for R10) and tested on chromosome 6 (HG01243 for R9, HG003 for R10), we found that FAST mode yielded slightly higher accuracy than HAC mode, while SUP mode yielded slightly lower accuracy for both R9 and R10 data (Supplementary Fig. S22). This observed dependency, coupled with the complex and non-monotonic relationship between basecalling accuracy and NanoFreeLunch accuracy, indicates that*

the specific error and QV characteristics introduced by different basecalling modes may have complex and hard-to-interpret effects on the features used by NanoFreeLunch for methylation prediction.”

Minor comments

Line 12. I'm not sure it's completely correct to say it requires no sample preprocessing, as there is still the necessary library prep for Nanopore sequencing.

We thank the reviewer to pointing this out. To avoid confusion, we have changed it to “*Nanopore sequencing has revolutionized the field of epigenomics by enabling direct detection of DNA methylation* ***without additional sample preprocessing such as bisulfite treatment***” in the revised manuscript.

Line 54: Consider changing to "…impact on the raw electrical signals detected by the sequencer."

We thank the reviewer for this comment. This is more accurate. We have changed manuscript according to the reviewer’s suggestion.

Line 62: Change "modification" to "methylation".

We have changed the wording in the revised manuscript.

Line 281: I believe it should be written as "… files of the R10.4.1 flowcell (abbreviated as R10)…"

We have changed manuscript according to the reviewer’s suggestion. The revised part is now at line 293 of the revised manuscript.

Reviewer #2: This is an interesting and useful approach to calling DNA methylation from ONT data when only the sequence data are available. As the author's comment, the vast majority of ONT data present in the public archives do not have the raw data available, and few of the sequence only datasets have methylation calls included (for example as tags in the BAMs). For this reason, a method to accurately call methylation using just the base calls and quality values would be very valuable.

I found the article to be well written and the example datasets were well chosen, showing the performance of their method with datasets called using various different sequencing and analysis pipelines. This gives confidence that their approach will be usable for many of the available ONT datasets.

We thank the reviewer for the insightful comments and for emphasizing the importance of our work. Below, we provide a point-by-point response to the comments.

I have just a few general comments:

(1) The authors discuss the performance of their method on cytosines in CpG context only (as far as I could tell). Have the authors tested their approach with other contexts? This would require testing on datasets from tissues with high-levels of non-CpG methylation such as stem cells or neural tissue.

Yes, our current study focuses exclusively on CpG methylation. While non-CpG methylation detection is a fascinating area for future work, we limited our scope to CpG contexts for two key reasons:

1. ONT-based CpG detection is well validated: Raw-signal-based methylation detection has been rigorously validated only for CpG contexts in independent studies (e.g., SEQC2, large-scale nanopore sequencing in Icelandic populations).
2. Lack of Non-CpG Standards: Though Dorado’s latest update offers non-CpG detection, no third-party validation exists for non-CpG accuracy.

To support future extensions, NanoFreeLunch’s training CLI allows retraining for non-CpG contexts once the field matures. We appreciate this suggestion and will explore it in follow-up work.

(2) Have the authors tested the approach on non-human (or invertebrate/plant samples? It would be interesting to know how general the approach is.

We agree with the reviewer that it is interesting to test if NanoFreeLunch can be applied to other species. We tested the pre-trained human model used in the study (trained on human data) on zebrafish (Danio rerio, s3://genomeark/species/Danio_rerio/fDanRer18/genomic_data/ont/pod5/) and rice (Oryza sativa, SRA ID is SRR16080273) data. The results show that despite of minor accuracy loss compared to the results on human data, the pre-trained model achieved a good

accuracy on the zebrafish data (PCC = 0.87). However, we observed a substantial accuracy drop in the rice data (PCC = 0.65). We hypothesize this discrepancy occurs because rice exhibits substantial non-CpG methylation, whereas CpG methylation dominates in zebrafish and humans (i.e., the same primary methylation motif). Non-CpG methylation may affect local and global QV patterns and error profiles, thereby affecting CpG methylation detection. Since the model trained on human data explicitly accounts for sequence context effects and performs well on the zebrafish data, it is unlikely that the reduced accuracy on the rice data is due to genomic sequence differences between humans and rice.

To test this hypothesis, we trained a model on Arabidopsis thaliana data (SRA ID: SRR16149191) and predicted CpG methylation in rice. These species have highly divergent genome sequences but both exhibit substantial non-CpG methylation (CHG and CHH). Results demonstrate NanoFreeLunch achieves high accuracy (PCC = 0.91) for rice CpG methylation prediction using the Arabidopsis-trained model. This indicates that for species with CpG-dominated methylation contexts, the pre-trained model is directly applicable. For species with substantial non-CpG methylation, retraining on species with identical methylation motifs is required.

(3) It would be useful to have some indication of the computational requirements. What would be the requirements (cpu, time, memory etc.) to call a 30x human genome for example?

We tested the model on a server equipped with 2× Intel Xeon Scalable Cascade Lake 6248 (2.5 GHz, 20 cores, 40 threads) CPUs and 12× Samsung 16 GB DDR4 ECC REG 2666 (totaling 192 GB RAM). The computational time for a single human genome is approximately 16 hours using the default chunk size of NanoFreeLunch. The chunk size impacts memory usage: larger chunks require more memory but improve runtime by reducing file I/O overhead. With the default setting, memory usage remains under 32 GB, which is within the capabilities of standard workstations or HPC clusters.

(4) As far as I can tell each cytosine is called independently of its neighbours. If this is correct, and given that methylation values are highly correlated between neighboring sites, have the authors considered using information from nearby cytosines?

We agree with the reviewer that neighboring CpG sites may offer valuable information for detecting DNA methylation. We explored this idea by incorporating the maximum error/QV correlation with neighboring CpG sites as an additional feature in an earlier version of NanoFreeLunch. However, we observed no improvement in accuracy. A possible explanation is that the current model already implicitly captures the correlation between neighboring CpG sites, as it includes joint error rates and QV correlations across all adjacent sites (including CpG sites, but without giving them special treatment).
