# Supplementary material for: Quantitative detection of DNA methylation from nanopore sequencing data without raw signals
Source: Gigascience. 2025 Oct 31;14:giaf113. doi: 10.1093/gigascience/giaf113 (PMC12576052; doi:10.1093/gigascience/giaf113)
Supplement: giaf113_Authors_Response_To_Reviewer_Comments_Original_Submission [file giaf113_authors_response_to_reviewer_comments_original_submission.pdf]

Author's Response To Reviewer Comments

(The complete response to the reviewers is provided in the file 'response-to-reviewers.docx', which may be found at the end of the system-generated PDF of the revised manuscript. Please note that the response included here is text-only and incomplete, as figures could not be uploaded through the submission system.)

We sincerely thank the reviewers for their constructive feedback. The comments are very helpful in improving the quality of this manuscript. Below, we provide a detailed point-by-point response to the reviewers' comments, and we highlight the changes in the revised manuscript. The page and line numbers cited in the responses below refer to the revised manuscript.

Reviewer reports:
Reviewer #1: In the manuscript "Quantitative Detection of DNA Methylation from Nanopore Sequencing Data without Raw Signals", the authors present a novel method to infer DNA methylation from sequence data without the need for the original signal data. This work describes an exciting new tool to utilize existing ONT data for epigenomics without the need for the massive POD5/FAST5 files.

We sincerely thank the reviewer for highlighting the significance of our study. We also appreciate the constructive comments and have addressed them point-by-point below.
Major comments

One of the major claims of this work is that it can remove the need to store POD5/FAST5 files which is expensive due to their size. However, they do not offer any indication of resource requirements for model training or training duration. This is particularly relevant to researchers who would like to leverage this tool but work on species other than human as they would likely need to train their own species specific model. Moreover, for other species, there is often far less data available to train such models. Can the authors offer a recommendation of the minimum training data requirements?

We agree with the reviewer that sample size for model training is important. To address this issue, we performed saturation analysis by downsampling the human training data (chr10 of HG01109) to 1%, 10%, 20%, 50%, 75%, and 90% respectively. The results (Fig. R1) show that accuracy plateaus at approximately 883,912 CpG sites (highlighted in red). While increasing sample size beyond this point yields no further gains, training with 117,855 CpG sites achieves an accuracy of 0.85 (PCC between NanoFreeLunch and Guppy), which represents 96% of the peak accuracy (PCC = 0.89) achieved with the full dataset (tested on chr6 of HG01243). This suggests that using roughly 118,000 CpG sites offers a favorable balance between data requirements and performance for the human data analyzed. While it is difficult to define an exact universal minimum training data requirement applicable to all species, these results provide valuable insights into the relationship between training sample size and accuracy. Researchers working on other species can adapt this saturation analysis to their own datasets to estimate suitable training sizes given their specific performance goals and data availability.

Line 289: The authors used the HAC option for basecalling. Did the authors test with different base calling models, I.e., SUP or FAST? Can the authors offer some discussion as to why they picked this model and whether or not they tested the other models? If they tested the other models was there a difference in the correlations?

Yes, we tested basecalling with FAST, HAC, and SUP modes. We found that the choice of basecalling mode has a minor, but measurable, impact on NanoFreeLunch's accuracy. The results show that FAST mode yielding slightly higher accuracy than HAC mode, and SUP mode yielding slightly lower accuracy for both R9 and R10 data (Fig. R2). In the previous version of the manuscript, we used HAC mode because it is commonly used and the default recommended mode in MinKNOW, the official sequencer software that wraps Guppy/Dorado for basecalling (https://nanoporetech.com/document/experiment-companion-minknow#starting-a-sequencing-run-on-promethion-p2i).

The exact reason for this discrepancy between basecalling modes is non-trivial to determine. Our results suggest that the relationship between basecalling accuracy and NanoFreeLunch accuracy is complex and non-monotonic. For instance, while R10 data has significantly higher basecalling accuracy than R9 data, NanoFreeLunch accuracy is similar for both flowcell types (Fig. R2). This indicates that the specific types of error/QV pattern introduced by different basecalling modes (FAST, HAC, SUP) may have complicated and hard-to-interpret effects on the underlying features used by NanoFreeLunch to predict methylation.

We added a paragraph in Discussion (page 22, line 241-255) as following and added Fig.R2 as Supplementary Fig. S22 to revised manuscript.
"In this study, we used HAC mode for basecalling, which is commonly used and recommended mode since it has a good balance between speed and accuracy. However, basecalling mode might affect methylation detection of NanoFreeLunch since different modes might have different error and QV patterns. Using models trained on chromosome 10 (HG01109 for R9, HG002 for R10) and tested on chromosome 6 (HG01243 for R9, HG003 for R10), we found that FAST mode yielded slightly higher accuracy than HAC mode, while SUP mode yielded slightly lower accuracy for both R9 and R10 data (Supplementary Fig. S22). This observed dependency, coupled with the complex and non-monotonic relationship between basecalling accuracy and NanoFreeLunch accuracy, indicates that the specific error and QV characteristics introduced by different basecalling modes may have complex and hard-to-interpret effects on the features used by NanoFreeLunch for methylation prediction."

Minor comments

Line 12. I'm not sure it's completely correct to say it requires no sample preprocessing, as there is still the necessary library prep for Nanopore sequencing.

We thank the reviewer to pointing this out. To avoid confusion, we have changed it to "Nanopore sequencing has revolutionized the field of epigenomics by enabling direct detection of DNA methylation without additional sample preprocessing such as bisulfite treatment" in the revised manuscript.

Line 54: Consider changing to "…impact on the raw electrical signals detected by the sequencer."

We thank the reviewer for this comment. This is more accurate. We have changed manuscript according to the reviewer's suggestion.

Line 62: Change "modification" to "methylation".

We have changed the wording in the revised manuscript.

Line 281: I believe it should be written as "… files of the R10.4.1 flowcell (abbreviated as R10)…"

We have changed manuscript according to the reviewer's suggestion. The revised part is now at line 293 of the revised manuscript.

Reviewer #2: This is an interesting and useful approach to calling DNA methylation from ONT data when only the sequence data are available. As the author's comment, the vast majority of ONT data present in the public archives do not have the raw data available, and few of the sequence only datasets have methylation calls included (for example as tags in the BAMs). For this reason, a method to accurately call methylation using just the base calls and quality values would be very valuable.
I found the article to be well written and the example datasets were well chosen, showing the performance of their method with datasets called using various different sequencing and analysis pipelines. This gives confidence that their approach will be usable for many of the available ONT datasets.

We thank the reviewer for the insightful comments and for emphasizing the importance of our work. Below, we provide a point-by-point response to the comments.

I have just a few general comments:
(1) The authors discuss the performance of their method on cytosines in CpG context only (as far as I

could tell). Have the authors tested their approach with other contexts? This would require testing on datasets from tissues with high-levels of non-CpG methylation such as stem cells or neural tissue.

Yes, our current study focuses exclusively on CpG methylation. While non-CpG methylation detection is a fascinating area for future work, we limited our scope to CpG contexts for two key reasons:
1. ONT-based CpG detection is well validated: Raw-signal-based methylation detection has been rigorously validated only for CpG contexts in independent studies (e.g., SEQC2, large-scale nanopore sequencing in Icelandic populations).
2. Lack of Non-CpG Standards: Though Dorado's latest update offers non-CpG detection, no third-party validation exists for non-CpG accuracy.
To support future extensions, NanoFreeLunch's training CLI allows retraining for non-CpG contexts once the field matures. We appreciate this suggestion and will explore it in follow-up work.

(2) Have the authors tested the approach on non-human (or invertebrate/plant samples? It would be interesting to know how general the approach is.

We agree with the reviewer that it is interesting to test if NanoFreeLunch can be applied to other species. We tested the pre-trained human model used in the study (trained on human data) on zebrafish (Danio rerio, s3://genomeark/species/Danio_rerio/fDanRer18/genomic_data/ont/pod5/) and rice (Oryza sativa, SRA ID is SRR16080273) data. The results show that despite of minor accuracy loss compared to the results on human data, the pre-trained model achieved a good accuracy on the zebrafish data (PCC = 0.87). However, we observed a substantial accuracy drop in the rice data (PCC = 0.65). We hypothesize this discrepancy occurs because rice exhibits substantial non-CpG methylation, whereas CpG methylation dominates in zebrafish and humans (i.e., the same primary methylation motif). Non-CpG methylation may affect local and global QV patterns and error profiles, thereby affecting CpG methylation detection. Since the model trained on human data explicitly accounts for sequence context effects and performs well on the zebrafish data, it is unlikely that the reduced accuracy on the rice data is due to genomic sequence differences between humans and rice.

To test this hypothesis, we trained a model on Arabidopsis thaliana data (SRA ID: SRR16149191) and predicted CpG methylation in rice. These species have highly divergent genome sequences but both exhibit substantial non-CpG methylation (CHG and CHH). Results demonstrate NanoFreeLunch achieves high accuracy (PCC = 0.91) for rice CpG methylation prediction using the Arabidopsis-trained model. This indicates that for species with CpG-dominated methylation contexts, the pre-trained model is directly applicable. For species with substantial non-CpG methylation, retraining on species with identical methylation motifs is required.

(3) It would be useful to have some indication of the computational requirements. What would be the requirements (cpu, time, memory etc.) to call a 30x human genome for example?

We tested the model on a server equipped with 2× Intel Xeon Scalable Cascade Lake 6248 (2.5 GHz, 20 cores, 40 threads) CPUs and 12× Samsung 16 GB DDR4 ECC REG 2666 (totaling 192 GB RAM). The computational time for a single human genome is approximately 16 hours using the default chunk size of NanoFreeLunch. The chunk size impacts memory usage: larger chunks require more memory but improve runtime by reducing file I/O overhead. With the default setting, memory usage remains under 32 GB, which is within the capabilities of standard workstations or HPC clusters.

(4) As far as I can tell each cytosine is called independently of its neighbours. If this is correct, and given that methylation values are highly correlated between neighboring sites, have the authors considered using information from nearby cytosines?

We agree with the reviewer that neighboring CpG sites may offer valuable information for detecting DNA methylation. We explored this idea by incorporating the maximum error/QV correlation with neighboring CpG sites as an additional feature in an earlier version of NanoFreeLunch. However, we observed no improvement in accuracy. A possible explanation is that the current model already implicitly captures the correlation between neighboring CpG sites, as it includes joint error rates and QV correlations across all adjacent sites (including CpG sites, but without giving them special treatment).

Editorial Manager®

Cookie Preference Center

We use cookies which are necessary to make our site work. We may also use additional cookies to analyze, improve and personalize our content and your digital experience. For more information, see our Cookie Policy.

You may choose not to allow some types of cookies. However, blocking some types may impact your experience of our site and the services we are able to offer. See the different category headings below to find out more or change your settings.

Allow all

Manage Consent Preferences

Strictly Necessary Cookies

Always active

These cookies are necessary for the website to function and cannot be switched off in our systems. They are usually only set in response to actions made by you which amount to a request for services, such as setting your privacy preferences, logging in or filling in forms. You can set your browser to block or alert you about these cookies, but some parts of the site will not then work.

Functional Cookies

These cookies enable the website to provide enhanced functionality and personalisation. They may be set by us or by third party providers whose services we have added to our pages. If you do not allow these cookies then some or all of these services may not function properly.

Performance Cookies

These cookies allow us to count visits and traffic sources so we can measure and improve the performance of our site. They help us to know which pages are the most and least popular and see how visitors move around the site.

Targeting Cookies

These cookies may be set through our site by our advertising partners. They may be used by those companies to build a profile of your interests and show you relevant adverts on other sites. If you do not allow these cookies, you will experience less targeted advertising.

Cookie List

Clear

Apply Cancel
Consent Leg.Interest

Confirm my choices

Powered by onetrust
