# Supplementary material for: Quantitative detection of DNA methylation from nanopore sequencing data without raw signals
Source: Gigascience. 2025 Oct 31;14:giaf113. doi: 10.1093/gigascience/giaf113 (PMC12576052; doi:10.1093/gigascience/giaf113)
Supplement: giaf113_Reviewer_1_Report_Original_Submission — Callum MacPhillamy, Ph.D. -- 2/9/2025 [file giaf113_reviewer_1_report_original_submission.pdf]

Reviewer Report

Title: Quantitative Detection of DNA Methylation from Nanopore Sequencing Data without Raw Signals

Version: Original Submission **Date:** 2/9/2025

Reviewer name: Callum MacPhillamy, Ph.D.

Reviewer Comments to Author:

In the manuscript "Quantitative Detection of DNA Methylation from Nanopore Sequencing Data without Raw Signals", the authors present a novel method to infer DNA methylation from sequence data without the need for the original signal data. This work describes an exciting new tool to utilize existing ONT data for epigenomics without the need for the massive POD5/FAST5 files.## Major commentsOne of the major claims of this work is that it can remove the need to store POD5/FAST5 files which is expensive due to their size. However, they do not offer any indication of resource requirements for model training or training duration. This is particularly relevant to researchers who would like to leverage this tool but work on species other than human as they would likely need to train their own species specific model. Moreover, for other species, there is often far less data available to train such models. Can the authors offer a recommendation of the minimum training data requirements?Line 289: The authors used the HAC option for basecalling. Did the authors test with different base calling models, I.e., SUP or FAST? Can the authors offer some discussion as to why they picked this model and whether or not they tested the other models? If they tested the other models was there a difference in the correlations?## Minor commentsLine 12. I'm not sure it's completely correct to say it requires no sample preprocessing, as there is still the necessary library prep for Nanopore sequencing.Line 54: Consider changing to "…impact on the raw electrical signals detected by the sequencer."Line 62: Change "modification" to "methylation".Line 281: I believe it should be written as "… files of the R10.4.1 flowcell (abbreviated as R10)…"

Level of Interest

Please indicate how interesting you found the manuscript: Choose an item.

Quality of Written English

Please indicate the quality of language in the manuscript: Choose an item.

Declaration of Competing Interests

Please complete a declaration of competing interests, considering the following questions:

- Have you in the past five years received reimbursements, fees, funding, or salary from an organisation that may in any way gain or lose financially from the publication of this manuscript, either now or in the future?
- Do you hold any stocks or shares in an organisation that may in any way gain or lose financially from the publication of this manuscript, either now or in the future?
- Do you hold or are you currently applying for any patents relating to the content of the manuscript?
- Have you received reimbursements, fees, funding, or salary from an organization that holds or has applied for patents relating to the content of the manuscript?
- Do you have any other financial competing interests?
- Do you have any non-financial competing interests in relation to this paper?

If you can answer no to all of the above, write 'I declare that I have no competing interests' below. If your reply is yes to any, please give details below.

I declare that I have no competing interests

I agree to the open peer review policy of the journal. I understand that my name will be included on my report to the authors and, if the manuscript is accepted for publication, my named report including any attachments I upload will be posted on the website along with the authors' responses. I agree for my report to be made available under an Open Access Creative Commons CC-BY license (http://creativecommons.org/licenses/by/4.0/). I understand that any comments which I do not wish to be included in my named report can be included as confidential comments to the editors, which will not be published.

Choose an item.

To further support our reviewers, we have joined with Publons, where you can gain additional credit to further highlight your hard work (see: https://publons.com/journal/530/gigascience). On publication of this paper, your review will be automatically added to Publons, you can then choose whether or not to claim your Publons credit. I understand this statement.

Yes Choose an item.
